# Ethnic-specific associations between dietary consumption and gestational diabetes mellitus incidence: A meta-analysis

**DOI:** 10.1371/journal.pgph.0000250

**Published:** 2022-05-11

**Authors:** Harriett Fuller, J. Bernadette Moore, Mark M. Iles, Michael A. Zulyniak

**Affiliations:** 1 Nutritional Epidemiology Group, School of Food Science and Nutrition, University of Leeds, Leeds, United Kingdom; 2 Leeds Institute of Medical Research, University of Leeds, Leeds, United Kingdom; 3 Leeds Institute for Data Analytics, University of Leeds, Leeds, United Kingdom; University of the Witwatersrand, SOUTH AFRICA

## Abstract

Globally, one in seven pregnant women are diagnosed with gestational diabetes mellitus (GDM), conferring short- and long-term health risks to both mother and child. While dietary prevention strategies are common in clinical practice, their effectiveness in different ethnicities is uncertain. To better inform prevention strategies, here the effects of unhealthy and healthy diets on GDM risk within distinct ethnic or cultural populations and geographic regions were evaluated and summarised. Pubmed, Scopus, Cochrane and OVID were systematically searched to identify randomised controlled trials (RCTs) and observational studies that investigated diet and GDM. A grouped analysis of common ‘healthy’ and ‘unhealthy’ diets was performed first, before analysing individual dietary patterns (e.g., prudent, Mediterranean). Random effect models and dose response analyses were performed where possible. PROSPERO (CRD42019140873). Thirty-eight publications provided information on 5 population groups: white European (WE), Asian, Iranian, Mediterranean and Australian. No associations were identified between healthy diets and GDM incidence in RCTs in any population. However, when synthesizing observational studies, healthy diets reduced odds of GDM by 23% (95% CI: 0.70–0.89, p<0.001, I^2^ = 75%), while unhealthy diets increased odds of GDM by 61% (95% CI: 1.41–1.81, p<0.0001, I^2^ = 0%) in WE women. No evidence of consistent effects in other populations were observed, even when adequately powered. Diet consistently associated with GDM risk in WEs but not in other populations. Heterogenous use and reporting of ethnically and culturally appropriate diets and dietary assessment tools, particularly in RCTs, raises uncertainty regarding the lack of association found in non-WE populations. Future studies require the use of culturally appropriate tools to confidently evaluate dietary and metabolic mediators of GDM and inform culturally-specific dietary prevention strategies.

## Introduction

Gestational diabetes mellitus (GDM), hyperglycaemia that develops during pregnancy, is diagnosed in 14% of pregnancies globally and is associated with numerous health risks [1]. Pregnancy and long-term complications for the mother include antepartum and postpartum haemorrhage, post-natal depression, and risk of type 2 diabetes (T2D; 7-fold) and cardiovascular disease (CVD; 2.3 fold) [2–6]. For offspring of GDM-mothers, risks include adulthood obesity and T2D [3] and future risk of GDM (~ 9 fold) [7]. These morbidities underline the need for effective GDM prevention [8]. Women from ethnic minority groups (e.g., African, Asian) disproportionally suffer from GDM compared to white European (WE) women (15% vs 5% prevalence) [9], largely independent of country of residence or general health. This suggests that underlying features and characteristics unique to ethnic minority groups and their cultures [10] are driving GDM risk disparity, and positions ethnicity as a major disease determinant. However, despite evidence of ethnic-specific associations between diet and maternal health during pregnancy [11, 12], and the benefit of ethnic-specific diets versus standard care [13], the majority of evidence on dietary prevention of GDM is from WE populations [14].

Indeed, in a systematic review of 18 randomised controlled trials (RCTs), fewer than 50% reported population ethnicity (of which all were primarily of WE decent); and only 1 study investigated an ethnic-specific intervention [13]. While one meta-analysis of 11 RCTs across 7 countries (n = 2,838) noted ethnicity as a moderator of diet-GDM associations (RR: 0.75, 95% CI: 0.60,0.95), it did not report ethnic-specific effects [15]. Evidence of ethnic-specific associations are more often reported in observational studies but evidence between observational studies are rarely compared. For example, in a WE cohort, meat-based and plant-based diets increased and decreased odds of GDM (odds ratio(OR)_meat_: 1.38; 95% CI: 1.14, 1.68; and OR_plant_ = 0.84; 95% CI: 0.68, 1.04) [16], whereas in a Chinese cohort comparable diet patterns showed the opposide (OR_meat_ = 0.89; 95% CI: 0.58, 1.36; OR_plant_ = 1.04: 95% CI; 0.90, 1.20) [17]. In spite of the urgent need to inform global health strategies, no meta-analysis has yet evaluated the effect of diet on GDM development in an ethnic-specific manner. Therefore, national GDM prevention strategies are often biased towards evidence from WE studies [18–22].

To address this and better inform prevention stratergies we have evaluated and summarised the effects of healthy and unhealthy dietary patterns on GDM incidence within distinct populations.

## Methods

### Search strategy and selection criteria

Searches were structured using PICO and MESH indexing with key terms (and synonyms thereof) for pregnancy (P), diet (I), ethnicity (C) and gestational diabetes (O); and study designs (PROSPERO:CRD42019140873) (*Appendix A in S1 Text*). Ovid, Cochrane (including trial registries), Scopus, and PubMed databases were searched from inception until January 2021. Citation lists of included studies were searched for additional relevant studies until no further articles were identified.

Eligible studies at title-abstract screening were human randomised control trials (RCTs) and observational studies (with the exception of non-nested case-control) that explored the association of diet and GDM (ORs, RRs, or raw data) published in English. Studies were excluded if they: (i) follow-up was < 1 trimester or started in the third trimester [23]; (ii) included unhealthy participants; (iii) combined diet with other lifestyle interventions; (iv) did not report participant ethnicity or nationality. Abstracts were screened in duplicate, with disagreements mediated by a third reviewer. Where effect estimates were adjusted for ethnicity without stating the ethnicity, it was assumed to be the ethnic majority (≥60% of population). Where this was not possible to confirm, corresponding authors were contacted. If no additional data were obtained the study was excluded.

### Data analysis

Data extraction was performed in duplicate for all variables (*Tables A and B in S1 Text)*. RCTs and observational studies were analysed separately. Healthy and unhealthy diet categories were defined based on: (i) study authors’ definition, or (ii) common definitions according to major health bodies (e.g., WHO, WCRF, NHS UK, ADA) [19, 21, 22, 24]. In general, ‘healthy’ diets were characterised by fruit and vegetables, wholegrains, fish, lean meats, and unsaturated fats; while ‘unhealthy’ diets were characterised by red/ processed meats, fried foods, confectionary, sugar sweetened beverages (SSBs), saturated fats, and added sugars. Where it was difficult to group with confidence, diets were unclassified. Unclassified diets required ≥2 studies to be considered.

Where exposure data were presented categorically, highest consumers were compared to lowest consumers. Where multiple models were detailed, effect estimates from fully-adjusted were extracted. To allow for a qualitative comparison between RCTs and observational evidence, risk ratios (RRs) were converted to odds ratios (Ors) with 95% confidence intervals (CI). All meta-analyses used generic inverse variance weighted random-effects (DerSimonian-Laird;DL) in Review Manager 5.3 (Cochrane) [25]. In meta-analyses with < 5 studies, the Hartung-Knapp-Sidik-Jonkman (HKSJ) random effects model [*meta* package (V4.9–6); R(v.1.2.5019)] was also used [26–28]. Bonferroni correction was applied as necessary. Uncertainty intervals for I^2^ values were also calculated [29]. Sensitivity analyses were performed where I^2^≥40% [30] and for GDM confounders: (i) timing of diet recall/intervention, (ii) maternal age, and (iii) overweight/obese. To account for differences in lean and overweight/obese classification between ethnic groups, a cut-off of BMI≥25 kg/m^2^ was used to classify overweight/obese in non-Asian populations, while in Asian populations a BMI cut-off of ≥23 kg/m^2^ was used [31].Where possible, dose response relationships were examined using the *dosresmeta* package in R (v.1.2.5019). See *Supplementary Methods in S1 Text*. Figures were produced within R studio through the use of the rworldmap and ggplot2 packages [32, 33].

### Risk of Bias (ROB) assessment

ROB was assessed using a modified 2016 Academy of Nutrition and Dietetics tool [34]. Comprised of 10 validity questions, it is designed for nutrition research and is translatable to RCTs and observational studies. Full methodological details in supplementay material (*Appendix B in S1 Text*).

### Power analysis

Post-hoc power analyses were undertaken using fixed-effects (*τ*^2^ = 0) or random-effects (*τ*^2^>0) methods [29]. Power ≥80% was considered adequate and based on recent meta-analyses investigating the association between common healthy and unhealthy diets on GDM in Europe, US and Asian populations [12, 35, 36], we considered a meaningful change in effect size as a difference in OR ≥ |0.20| between the high and low exposure groups. Exposed and unexposed were calculated as an average of the exposed/ unexposed sizes of all studies for the exposure.

## Results

**Fig 1:**
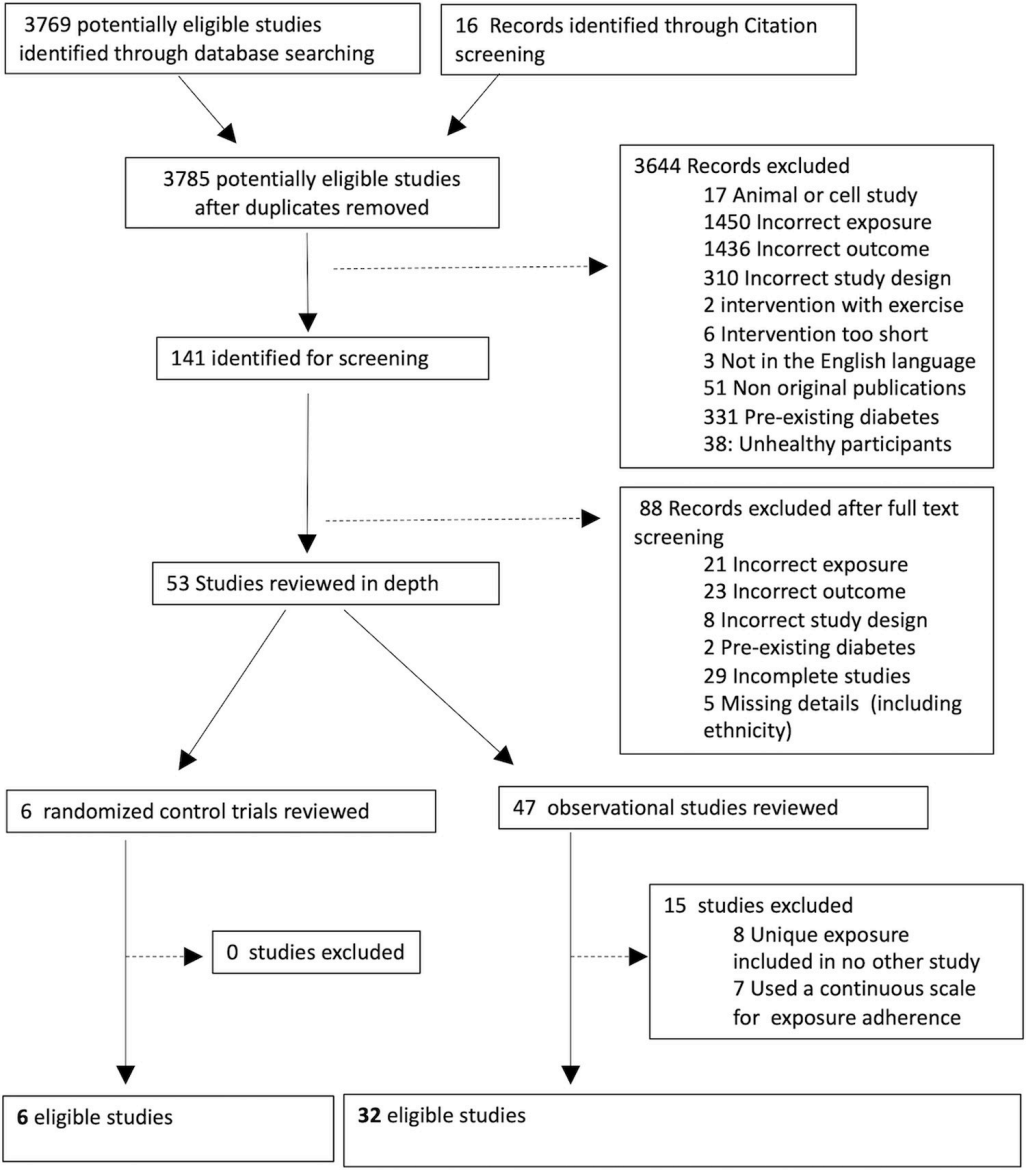
Prisma diagram. Outline of identification of studies in Ovid (AMID, CAB abstracts, EBM, EMBASE, Global Health, Health Care Management Information Consortium, MIDRIS, OVID Medline R), Cochrane (including trial registries), Scopus, and PubMed and record of screening process of articles.

After the removal of duplicates, 3393 studies were identified, from which 53 studies progressed to full text screening (*Fig 1*). Of these, 25 studies reported heathy diets [‘healthy recommendations’ (n = 13), ‘Mediterranean diet’ (n = 6), ‘prudent diet’ (n = 4), ‘plant-based’ (n = 6) or ‘healthy snack’ (n = 1)], and 13 reported unhealthy diets [‘western diet‘ (n = 6), ‘fried/fast food’ (n = 4), ‘sweet and seafood’ (n = 2), and ‘unhealthy diet score’ (n = 1)]. Additional diets considered neither healthy or unhealthy were grouped as ‘unclassified’ [meat pattern, high protein diet, ‘traditional Asian’, ‘high-fat’, ‘high-carbohydrate’, ‘high-animal protein’, ‘high-vegetable protein’ and ‘high-fish’ diets] when ≥2 studies were identified (*Appendix C in S1 Text*). This provided a total of 38 studies (6 RCTs and 32 observational;n = 251,778 participants) for review and meta-analyses (*Fig A in S1 Text*), 28 of which included more than one exposure, across 4 distinct ethnic and geographic groups: white European (WE; 83%), Asian (9%; East and South Asian), Mediterranean (i.e. southern European populations) [37] (5%), and Australian Nationals (3%;Australian residents with an almost equal proportion of Asians and WEs). (*Fig B in S1 Text)* Maternal age of study participants ranged 24–36 years and 14/38 studies reported an average BMI of overweight or obese. All included RCTs used a 75g oral glucose tolerance test (OGTT) for GDM diagnoses, while in observational studies, the majority of studies (20/32; 62.5%) used OGTT with clearly defined criteria to diagnose GDM. A chi-square test reported no significant difference (P > 0.05) in the use of OGTT between ethnic groups in observational studies.

### RCTs

**Fig 2:**
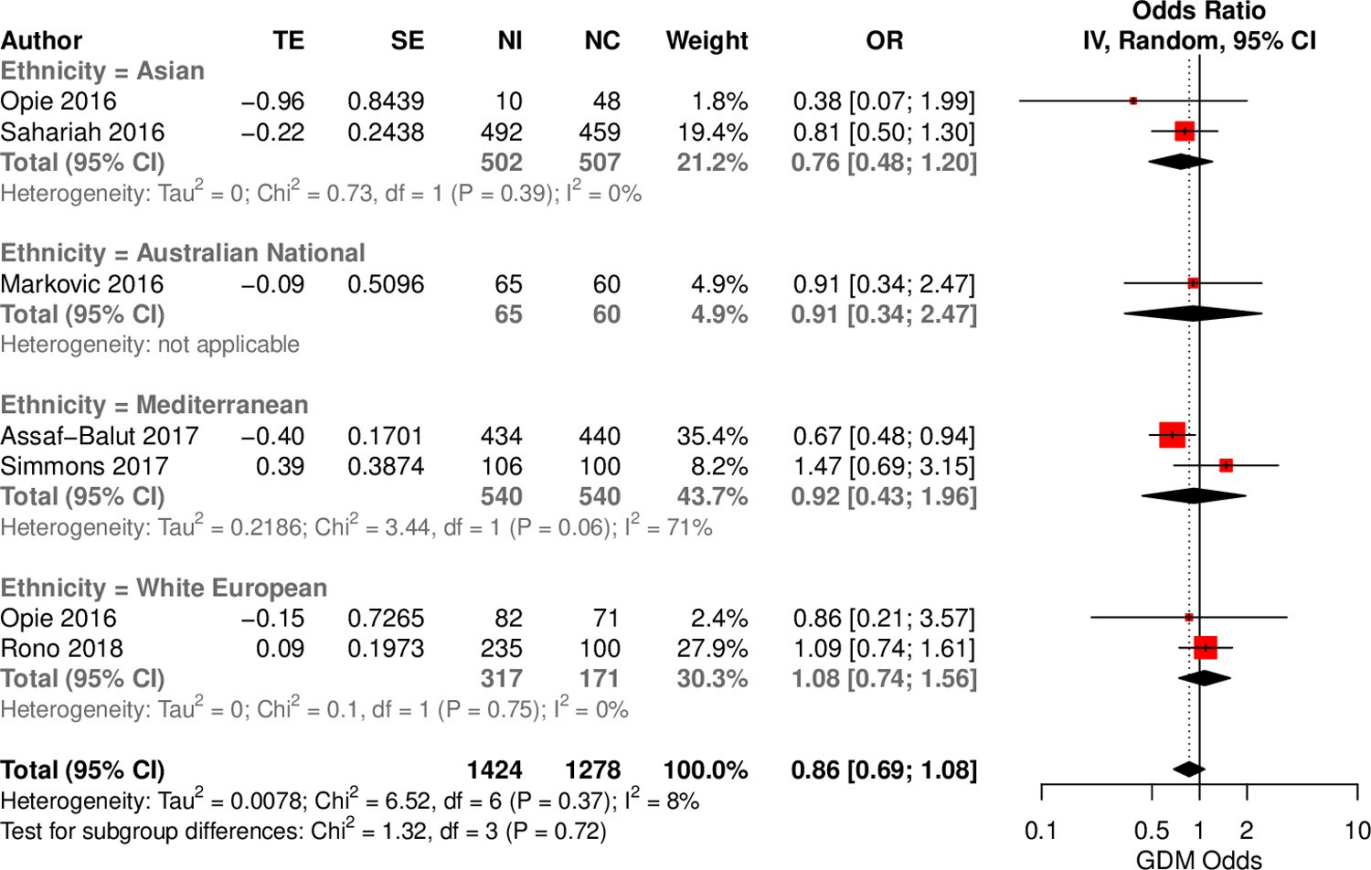
Forest plot of reported effect sizes of healthy dietary interventions on GDM in ethnic–specific RCTs. Forest plot to investigate the association between heathy diets and GDM, investigated within distinct ethnic groups. TE: treatment effect, SE: standard error, IV: inverse variance method, CI: Confidence interval.

Six RCTs (n = 3,041), including 4 population groups (Asian, Australian, Mediterranean, WE) evaluated the impact of a healthy diet compared to control (*Table A in S1 Text*). No effect of healthy diets on GDM was found across across ethnic groups (*Fig 2*). For individual diets, stratified by ethnicity, no associations were identified for healthy recommendations (n = 4) or healthy snacks (n = 1). However, one study reported a protective effect of the Mediterranean diet against GDM (OR = 0.67; 95%CI: 0.48, 0.94) in a Mediterranean population (*Fig C in S1 Text*). No differences were observed with the HKSJ model. No RCTs reported on the effect of an unhealthy diet (*Table C in S1 Text*). Only 3 of the 5 studies in non-white Europeans used a culturally validated questionnaire (*Table I in S1 Text)*. The analysis was adequately powered to detect a 4% change in odds (*Table D in S1 Text*).

### Observational studies

In total 32 observational studies (n = 248,737), reported on 5 ethnic groups (Asian, Australian Nationals, Iranian, Mediterranean and WE) across 20 countries (*Table B in S1 Text*). Within these studies, 17 dietary patterns were reported and grouped as healthy, unhealthy, or unclassified. The majority of observational studies in non-white Europeans (14/16) reported the use of a validated dietary assessment tool (*Table I in S1 Text)*.

#### Healthy diets

**Fig 3:**
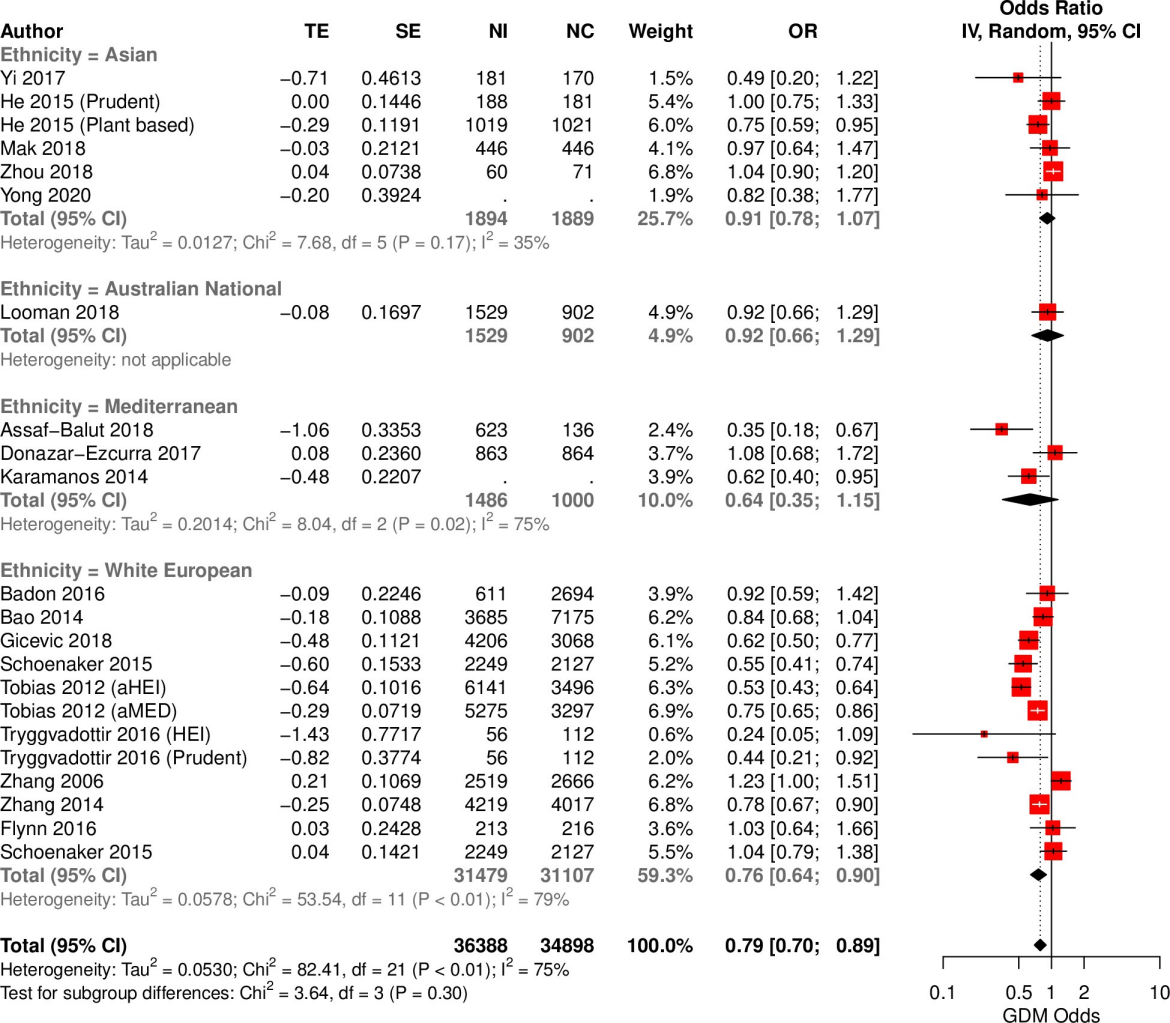
Forest plot of reported associations between healthy dietary interventions and odds of GDM in ethnically–defined observational studies. Forest plot illustrating the association between habitual healthy dietary intake and GDM in distinct ethnicities. TE: treatment effect, SE: standard error, IV: inverse variance method, CI: Confidence interval.

20 studies reported the consumption of healthy diets (healthy recommendations, Mediterranean, prudent, and plant-based diets) in 4 ethnic groups. When assessed as a single multi-ethnic group, high adherence to a healthy diet associated with a reduction in odds of GDM (OR = 0.78; 95%CI: 0.70,0.88; I^2^ = 74%), compared to lowest adherence (*Fig 3*). An inspection by ethnicity showed that healthy diets are protective in WEs (OR = 0.75; 95%CI: 0.65 0.88; I^2^ = 79%) but not other ethnic groups. A subsequent analysis of distinct healthy diets, found healthy recommendation and Mediterranean diet patterns to be protective against GDM (*Fig D in S1 Text*), with both associations driven by WEs (OR = 0.70; 95%CI: 0.56,0.86; I^2^ = 70% and OR = 0.66; 95% CI: 0.50, 0.85; I^2^ = 65%). When the more stringent HKSJ model was applied, only healthy recommendations retained significance (*Table C in S1 Text)*. No associations were observed in Asian populations even after stratification by East Asian (Chinese and Japanese) and South/South-East Asian regions (Indian subcontinent and Malaysian) (*Table E in S1 Text*). All analyses were adequately powered to detect a change in odds ≥ 6% (*Table C in S1 Text*).

#### Unhealthy diets

**Fig 4:**
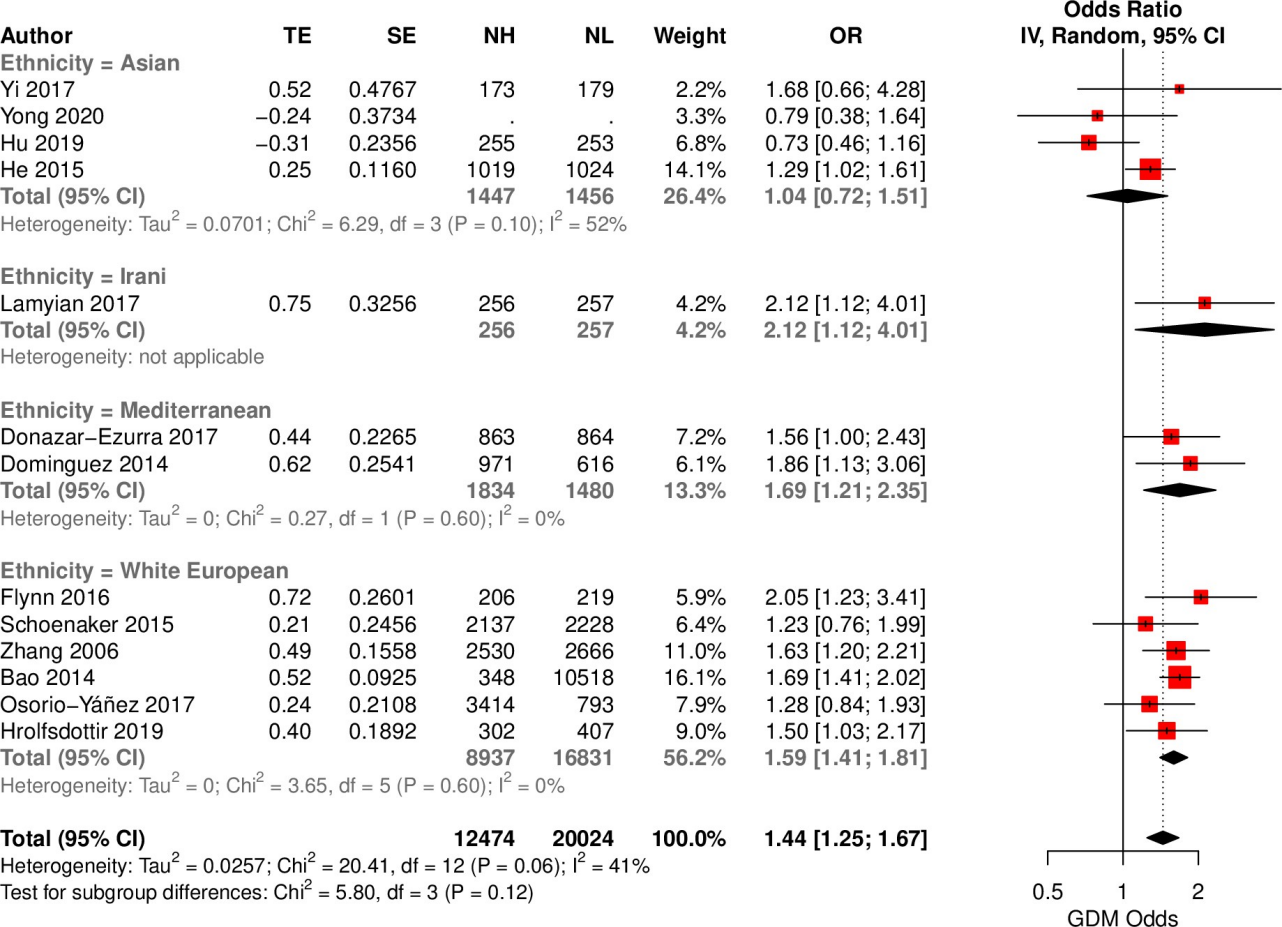
Forest plot of reported associations between unhealthy dietary interventions an odds of GDM in ethnically–defined observational studies. Forest plot illustrating the association between habitual unhealthy dietary intake and GDM in distinct ethnicities. TE: treatment effect, SE: standard error, IV: inverse variance method, CI: Confidence interval.

Thirteen studies reported the association between an unhealthy diet (Western, fried/ fast food, unhealthy diet score, sweet and seafood pattern) and GDM across 4 ethnic groups. In a large multi-ethnic cohort, high adherence to an unhealthy diet associated with increased odds of GDM (OR = 1.44; 95%CI: 1.25,1.67, I^2^ = 12%), compared to low adherence (*Fig 4*). This association was also observed in distint ethnic group [WEs (OR = 1.59; 95%CI: 1.41, 1.81, I^2^ = 0), Mediterranean (OR = 1.69, 95% CI: 1.21, 2.35, I^2^ = 0), and one Iranian (OR = 2.12; 95%CI: 1.12, 4.01, I^2^ = NA)] aside from Asians. When considering individual unhealthy diets, the Western diet increased odds of GDM (OR = 1.51; 95%CI: 1.23,1.86; I^2^ = 7%) in the overall population, primarily driven by WEs (OR = 1.60; 95%CI: 1.26,2.02; I^2^ = 3%) and one Mediterranean study (OR = 1.56; 95% CI: 1.00,2.43; I^2^ = NA) ‒ using HKSJ, WEs achieved P = 0.07 (*Fig E and Table C in S1 Text).* The fried/fast food diet pattern associated with increased odds of GDM (OR = 1.66; 95%CI: 1.42,1.92; I^2^ = 0%), with comparable effect sizes across ethnic groups. All analyses were adequately powered to detect a change in odds ≥ 9% (*Table D in S1 Text*).

#### Unclassified diets

Four dietary patterns (meat-based, high-protein, traditional Asian, and high-fish) were unclassified.(*Fig F in S1 Text*) The meat-based pattern (n = 7) associated with increased odds of GDM when evaluated in a multi-ethnci cohort (OR = 1.41, 95% CI:1.22,1.63, I^2^ = 0), with only small deviations of effect sizes between ethnicities. A high-fish diet (n = 5) was protective against GDM in WEs (OR = 0.85, 95% CI: 0.75, 0.98, I^2^ = 0) but not in Asians. No associations were observed between (i) high-protein diet and GDM or (ii) a traditional Asian (n = 2) diet and GDM in Asians. All analyses were adequately powered (80%) with varied thresholds for each analysis (0.5–18% change in odds) (*Table D in S1 Text*).

#### Macronutrients

Four diets classified diet exposure based on a dominant macronutrient (% total energy): animal protein, vegetable protein, fat, and carbohydrate. (*Fig G in S1 Text*) Animal protein (OR = 1.49, 95%CI:1.25,1.77,I^2^ = 0), carbohydrate (OR = 0.49, 95% CI:0.38,0.63; I^2^ = 0), and fat diet patterns (OR = 1.50, 95%CI: 1.22,1.83, I^2^ = 0) all associated with odds of GDM. Within ethnic groups, comparable effect sizes were observed for Asians and WEs. All associations remained significant after Bonferroni correction following the HKSJ approach, with the exception of the animal protein diet in Asians. No associations were observed for the vegetable protein exposure. All analyses were adequately powered (80%) with varied thresholds for each analysis (0.5–13% change in odds) (*Table D in S1 Text*).

#### Dose response analyses

When evaluated as a multi-ethnic population, no associations were observed. When ethnic groups were investigated independently, a positive non-linear dose-response relationship was found for WEs consuming a fried food diet but it did not pass Bonferroni correction.

### Post-hoc analysis—Combined RCT & observational analysis

Effect estimates from RCTs and observational studies were of similar magnitude with overlapping confidence intervals, comparable I^2^ values, and ROB scores (*Fig H in S1 Text*). This suggests that the primary source of heterogeneity may not be study design. Therefore, a post-hoc analysis with combined RCTs and observational studies was undertaken. Power improved in all ethnic subgroups. This analysis uncovered a novel association between healthy diets and GDM in Australian nationals (OR = 0.92, 95% CI: 0.88,0.97; I^2^ = 0) with only negligible changes in effect sizes and no other new associations identified (*Table C in S1 Text).*

### Sensitivity analyses–Confounders

Sensitivity analyses were undertaken on all exposures with I^2^≥ 40%. Sensitivity analyses were performed based upon: (i)diet during pregnancy; (ii)adjustment for obstetric risk factors (parity, gravidity or multiple pregnancy); and (iii) pre-pregnancy BMI; and (iv) maternal age. When only considering dietary intake during pregnancy, no association was found between healthy diets and GDM in any ethnic group. In addition, when considering the overall unhealthy or the western diets, no association was found with GDM in overweight/obese WEs. Interestingly, a high protein diet increased odds of GDM in older women that was driven by WEs (OR = 1.28; 95%CI: 1.09–1.52; I^2^ = 0). (*Tables D–H in S1 Text*). All sensitivity analyses were well powered (0.80%) to detect an effect size ≥10% with the majority suitably powered to detect an effect size ≥ 5%. Two exceptions were the assessments of the plant-based diet in overweight women and healthy diets in overweight/obese Asian women (*Table D in S1 Text*). A single sensitivity analysis of the effect of the western diet during pregnancy had inadequate power to detect a change in odds of 20%.

### Risk of Bias (ROB)

Evidence of moderate ROB was found but no study exceeded the Academy of Nutriton and Dietetics’ exclusion threshold. Within RCTs 45% and 35% scored low or neutral ROB, with 21% at risk of bias. Within observational studies, 55% and 22% scored a low or neutral risk of bias, with 24% at risk of bias. Areas of concern were participant selection, management of withdrawals, and study group comparability. Carbohydrate, fat, and Mediterranean diets were at highest risk of bias while the prudent and fast food diets were at low risk. No evidence of publication bias was identified. (*Figs I–L in S1 Text).*

## Discussion

The aim of this work was to offer clarity regarding the ability of diet to mitigate GDM in different ethnic groups. Evidence from RCTs and observational studies reporting healthy, unhealthy, and unclassified dietary patterns were systematically reviewed and meta-analysed. The results confirm a protective association between healthy diets and an adverse association between unhealthy diets and GDM in WEs and Ausrlian nationals, with evidence in ethnic minority groups hindered by fewer studies (<20% of studies), and limited use of ethnically informed methods. In minority populations where ≥2 studies were available, only carbohydrate rich diets were associated (protectively) with GDM in Asians.

The majority of RCTs we collected (5/6 studies) commenced during pregnancy. A meta-analysis of 5 RCTs (n = 1,155) agree with our findings and report no significant effect of dietary interventions on GDM risk in WEs. Interestingly, a recent meta-analysis with 37 RCTs reported that diets designed to manage gestational weight gain (GWG), reduced GDM incidence in Asian countries but not WE-majority countries [38]. This is contrary to our results but may explained by the aims of the interventions, with GDM diets focussed on reducing glycaemic loads rather than total caloric intake. Therefore, it may be that (as a mediator of dysglycemia) controlling GWG is key and that future dietary interventions to reduce GDM risk in Asian countries require greater emphasis on weight management.

Observational studies often focus on pre-pregnancy diet, making them crucial to understand how pre-conception dietary habits influence GDM. A systematic review of 34 observational cohort and case-control studies, reported that high consumption of cholesterol, heme iron, and processed meat increased risk of GDM, while patterns rich in fruit, wholegrains and vegetables reduced risk of GDM [39]. However, a high heterogeneity between ethnic groups was observed by the authors—likely due to confounding from ethnic-specific food preferences, cooking methods, and meal times. To address this, we performed our meta-analyses in ethnic-specific subgroups; thereby, minimsing confounding within each ethnic analysis while permitting a comparison of effect sizes between them. Fifteen dietary exposures were identified in multiple studies, and classified as either ‘healthy’, ‘unhealthy’ or ‘unclassified’ (i.e., neither healthy nor unhealthy) diets. Following stratification by ethnicity, the protective effect of healthy diets against GDM was confirmed in WEs and the hazardous effect of unhealthy and meat-based diets in WEs; however, consistent evidence of an association within non-WE groups was not found. Interestingly, all associations were unaffected by mother’s age and BMI, suggesting that modified guidelines for WE women at high-risk of GDM due to age or BMI may not be required. The presence of an association in WEs within observational studies but not RCTs could be a result of increased power or it could highlight the importance of a healthy diet prior to conception. However, future RCTs investigating dietary interventions during ‘family planning’ are required to test this hypothesis.

Interestingly, examining macronutrient-specific diets, those characterised by animal protein or fat increased odds of GDM by up to 50% in WE and Asians, whereas carbohydrate-rich diets reduced odds of GDM by ≈50%. Unfortunately, because all exposures were quantified as % energy intake, it was not possible to tease apart whether the protective effect on GDM was driven by reductions in protein and fat or increased consumptionof carbohydrate, or a combination thereof. Interestingly, while animal protein associated with GDM risk in WEs and Asians, no association was observed with the meat-based dietary pattern in Asians. While the animal-protein diet may have been carbohydrate-rich and negated the effects of high-animal protein, an alternative explanation may be that ethnic-specific foods and cooking methods are difficult to capture with some dietary recall tools. With limited associations identified in non-WE studies and recognising the similarities in effect sizes in this analysis of RCTs and observational studies, study types were combined [40]. This uncovered an association between healthy diets and GDM in Australian nationals, a heterogenous group comprised of WE and Asian mothers but no other additional associations were reported.

Despite numerous significant associations between dietary patterns and GDM in WEs, no consistent evidence was found in non-WE populations. The reason for this is unclear but inconsistent reporting (and use) of ethnically and culturally-informed assessment tools may have contributed to this [41]. Ethnically sensitive interventions consider dietary habits, food preparation, and cultural beliefs that are relevant to the study population to improve accuracy of dietary assessment [42, 43], rather than a single FFQ used across multiple ethnic groups that can introduce bias and ethnic-specific differences under/over-reporting [44, 45]. While many studies reported ethnically-modified approaches, some of the details regarding their modification and validity were unclear, particularly in randomised controlled trials where only 3 of 5 (60%) of studies in non-white European populations reported using a culturally validated questionnaire. This agrees with a systematic review (n = 42 studies) that reported a lack of validation of dietary assessment tools (only 17%) in studies undertaken in minority ethnic groups [46]. Interestingly, the majority of non-white observational studies included in this review (14/16; 87.5%) did use culturally appropriate dietary assessment tools, validated within a relevant population.

The use of metabolomics-based strategies may offer a method to characterise diet, metabolism, and the role of bioavailability across ethnic groups and expose underlying ethnic-specific requirements. Previous work in the Born in Bradford cohort has demonstrated that pregnant WEs and SAs have significantly different metabolic profiles (i.e., namely, lipoproteins, lipids, glycolysis metabolites, and amino acids) [47]. Work by the multi-ethnic NUTRIGEN consortium also suggests that a healthy plant-based diet consumed during pregnancy effects infant birth weight differently in SAs (increased birthweight) and WEs (reduced birth weight) [48]. This evidence suggests that ethnically-distinct dietary and underlying metabolic qualities exist between ethnicities and further highlights the need for ethnically-tailored interventions.

This is the first study to meta-analyse the effect of numerous dietary patterns and interventions consumed before or during pregnancy on GDM within distinct ethnic groups and geographic regions, using both RCTs and observational study data. Strengths included the fact that all data were examined using both standard methods (DL) as well as supplementary and more conservative analyses (HKSJ) when ≤5 studies were available [26–28]; along with robust sensitivity analyses for confounding factors. Moreover, a single ROB assessment that is translatable for both RCTs and observational studies permitted comparison of bias between study design. Finally, power analyses confirmed adequate power in WE and Asian analyses with limited power in other minority ethnic groups. However, this study did have limitations. First, only studies written in English were included, which many have limited the scope. Second, all observational studies are limited by confounding; however, the similar effect sizes between RCTs and observational studies that we observed may suggest that the adjustment for confounding in the included observational studies limited confounding reasonably well. Finally, there is a risk of type 2 errors due to the widespread use of the Nurses Cohort Study (NCS); however, sensitivity analysis demonstrated no impact on results when NCS studies were removed.

Through the use of both RCTs and observational studies, this meta-analysis confirms the presence of a protective effect of healthy diets against GDM in WE women and an increase in risk in mothers consuming unhealthy diets. Current evidence in ethnic minority groups is less certain because of fewer studies and, with limited evidence of an assocoaion in non-white ethnic groups. However, inconsistent reporting of ethnically appropriate diets or assessment tools, challenges the certainty of evidence within studies of minority ethnic groups. In summary, our work highlights that future studies in ethnic minority groups, using ethnically informed diets and tools, particularly RCTs, are urgently needed to accurately evaluate the effect of diets on GDM so that appropriate strategies for these high-risk populations can be confidently assessed and defined.

## Supporting information

S1 Text (DOCX)

S1 Graphical Abstract Visual abstract of a meta-analysis to investigate the effects of healthy and unhealthy diets on gestational diabetes risk across ethnic groups. (TIF)
