# Supplementary material for: Ethnic-specific associations between dietary consumption and gestational diabetes mellitus incidence: A meta-analysis
Source: PLOS Glob Public Health. 2022 May 11;2(5):e0000250. doi: 10.1371/journal.pgph.0000250 (PMC10021780; doi:10.1371/journal.pgph.0000250)
Supplement: S1 Text — (DOCX) [file pgph.0000250.s001.docx]

SUPPLEMENTARY INFORMATION

**Ethnic-specific associations between dietary consumption and gestational diabetes mellitus incidence: a meta-analysis.**

Authors: **Harriett Fuller, MSc, J. Bernadette Moore, PhD, Mark M. Iles, PhD, Michael A. Zulyniak, PhD**

Corresponding: **Michael Zulyniak , Nutritional Epidemiology Group, School of Food Science and Nutrition, University of Leeds, Woodhouse Lane, Leeds, LS2 9JT. Email. m.a.zulyniak@leeds.ac.uk.**

# SUPPLEMENTARY METHODS

## Sensitivity Analyses

The average age of mothers in each study was classed as young or old if the average age of mothers within the study was below or above the average age of a women during her first pregnancy within that country/region. Cut-off values for this analysis were obtained from the Office of National Statistics (UK), CIA (USA, Spain, Australia, Iceland, Finland, Japan, France, Italy, Singapore), OECD and EU average (Malaysia and multinational studies), China (Pulitzer Center), Iran (Worldbank) and India (Times of India). No sensitivity analysis was performed if all studies within the meta-analyses classified similarly for the same confounder (e.g., all studies of young mothers or of the same BMI category). No analysis was run for the obstetric adjustment sensitivity analyses if all studies within that exposure had accounted for obstetric risk factors.

## Dose Response Analysis

Where possible, the scales for an exposure were transformed onto the same scale. Studies using binary classification for exposure adherence (exposed and unexposed) were excluded prior to the development of cubic spline models due to the intractability of the algorithm when these studies were included. To minimise this limitation for quadratic models, an exponential curve was plotted using the OR’s for the exposed and unexposed groups as the maximum and minimum effect sizes. From this, the median dose was calculated which could be included in a new quadratic model (hereafter referred to as quadratic*). The sample size for the calculated OR data point was assumed to be the same as the ‘n’ exposed. When an inflection of the confidence interval was visible within these cubic spline models (shown by confidence intervals converging at the smallest point on a value other than 0) the graph was replotted using this value as the reference variable for easier interpretation of the model. 3k models (splines at 0.1, 0.5, 0.9) and 4k models (splines at 0.05,0.35, 0.65, 0.95) were run when possible - the limiting factor being lowest number of categories found within the studies included for a given one exposure. Wald test estimates were used to determine the significance of cubic spline relationship. Log likelihood (LogL) values were used to select the best fitting model.

## Risk of Bias (ROB) assessment

ROB was assessed using a modified version of the 2016 Academy of Nutrition and Dietetics tool ‘Evidence Analysis Manual: Steps in the Academy Evidence Analysis Process’. The tool contains 10 validity questions which can be assigned a positive score, a neutral score, or a negative score when the source of bias is likely to influence study conclusions. To aid the user in assigning a score, each validity question is composed of weighted ‘important’ sub-questions. To ensure a systematic approach, study authors developed a protocol in order to translate the scores of the sub-questions to the scores of the validity questions. When an ‘important’ sub-question and/or ≥ 50% of all sub-questions was assessed to be negative, the overall validity question was assigned as negative. When all sub-questions were assigned to be positive, the overall validity question was assigned to be positive. In situations meeting neither of the above scenarios, the validity question was assigned to be neutral.

SUPPLEMENTARY FIGURES

## Fig A. Schematic highlighting exposure characteristics.

Schematic of the number of studies and characterises of each exposure. 2 overarching exposures, healthy (n=25, 6 subsets) and unhealthy (n=13, 4 subsets) were included within this review. Exposures which did not match to either the healthy or unhealthy pattern were classed as unclassified and were analysed independently. TE: Total Energy


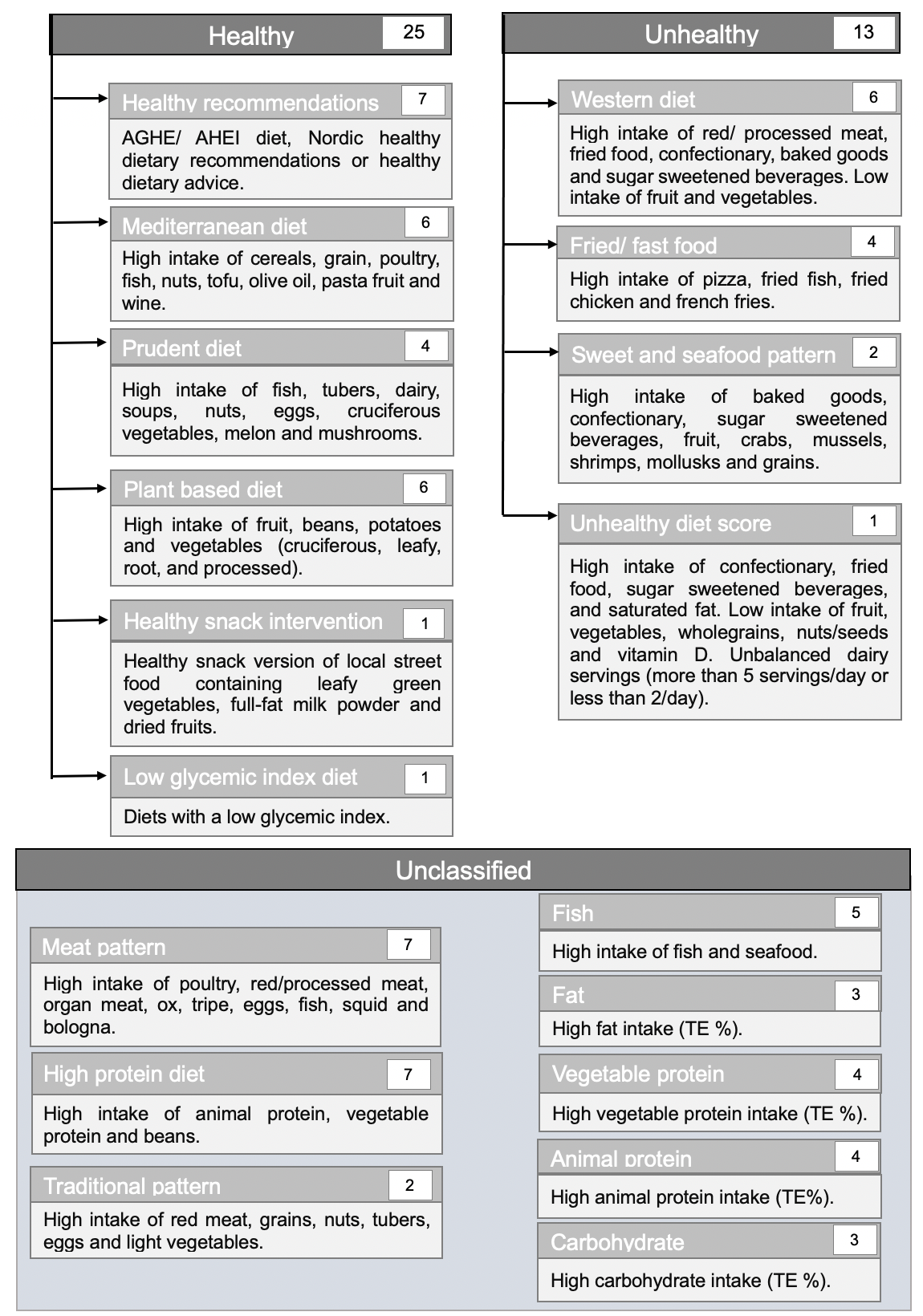


## Fig B: Map of included studies.


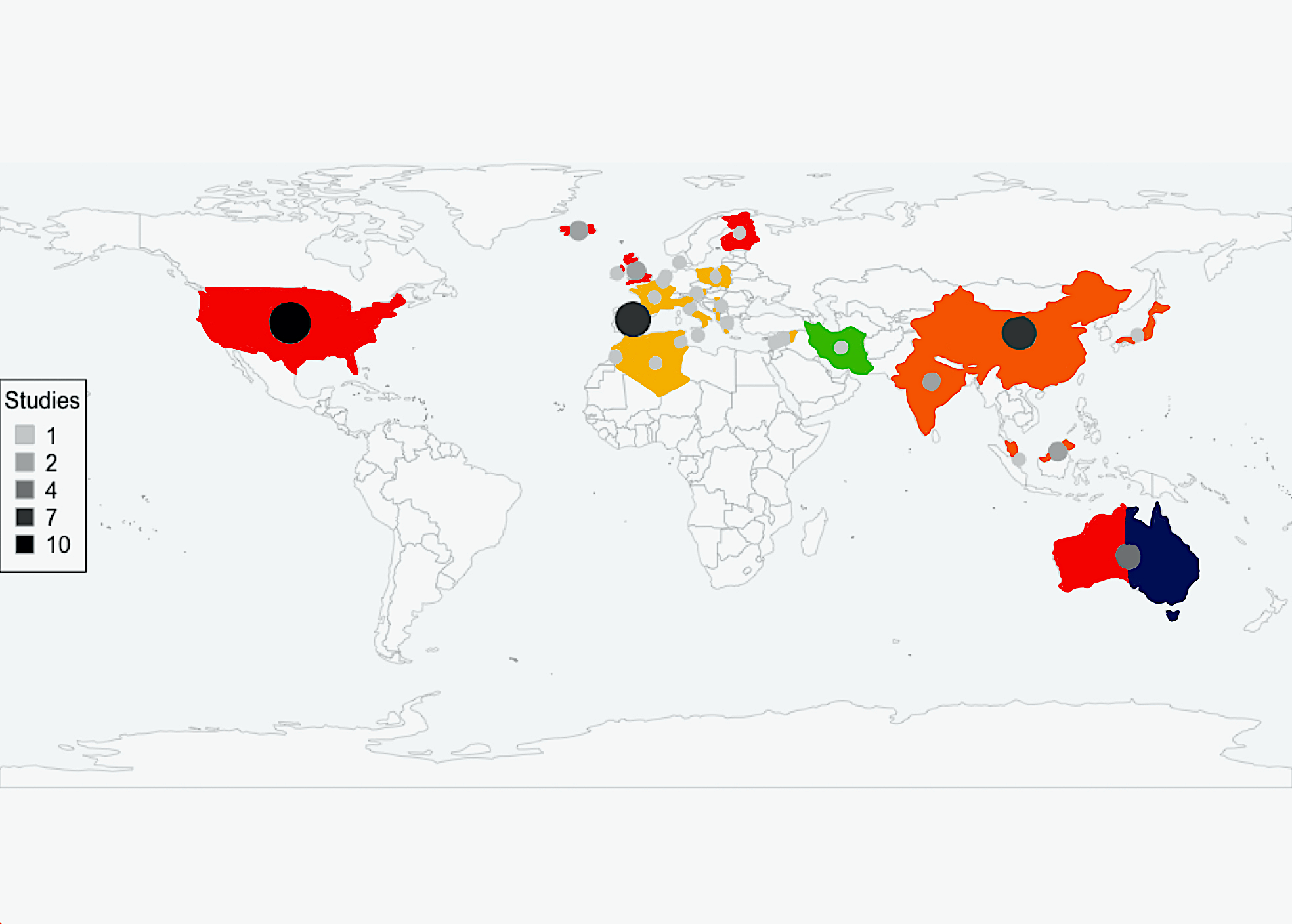
Global distribution of identified studies. Studies represented on the map is greater than the number of identified studies following exposure classification as some studies were multinational. The countries shading represents the ethnic subgroup that the country was linked to: red = white European, yellow = Mediterranean, green = Iranian, orange = Asian, navy=Australian National. (~60% WC and ~40% Asian). The baselined map was obtained from the *rworldmap* package within R studio^1^.

1South A. rworldmap: A New R package for Mapping Global Data. The R Journal. 2011;1(3):35-43

Fig C. Forest plots of RCTs included within the ‘Healthy diet’ exposure**.**

Associations between ‘healthy’ dietary interventions and GDM in RCTs stratified by ethnicity subgroups using a DerSimonian and Laird (DL) random effects meta-analysis: (A) Healthy recommendations, (B) Mediterranean diet, and (C) Healthy snacks. TE: treatment effect, SE: standard error, IV: inverse variance method, CI: Confidence interval.


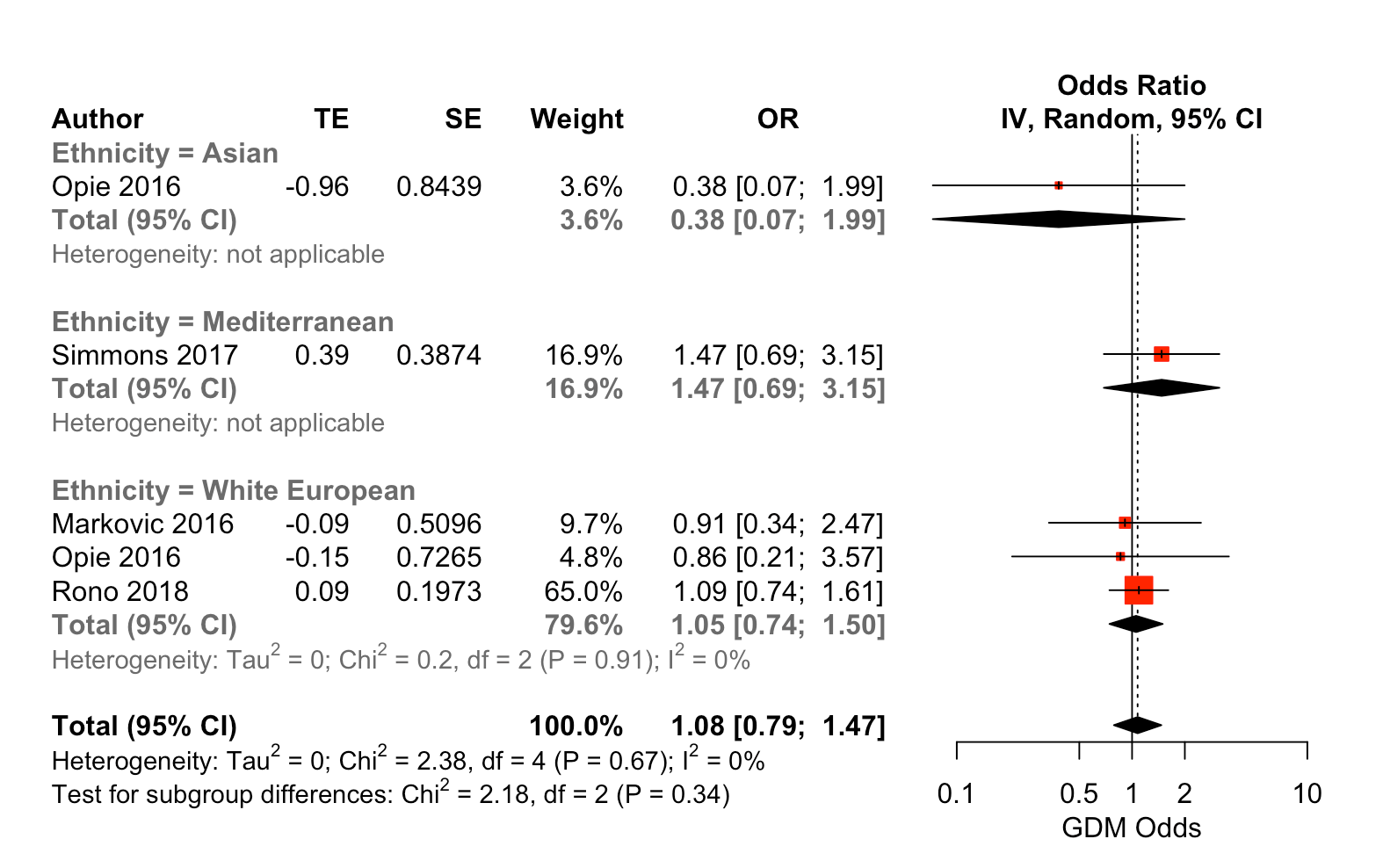

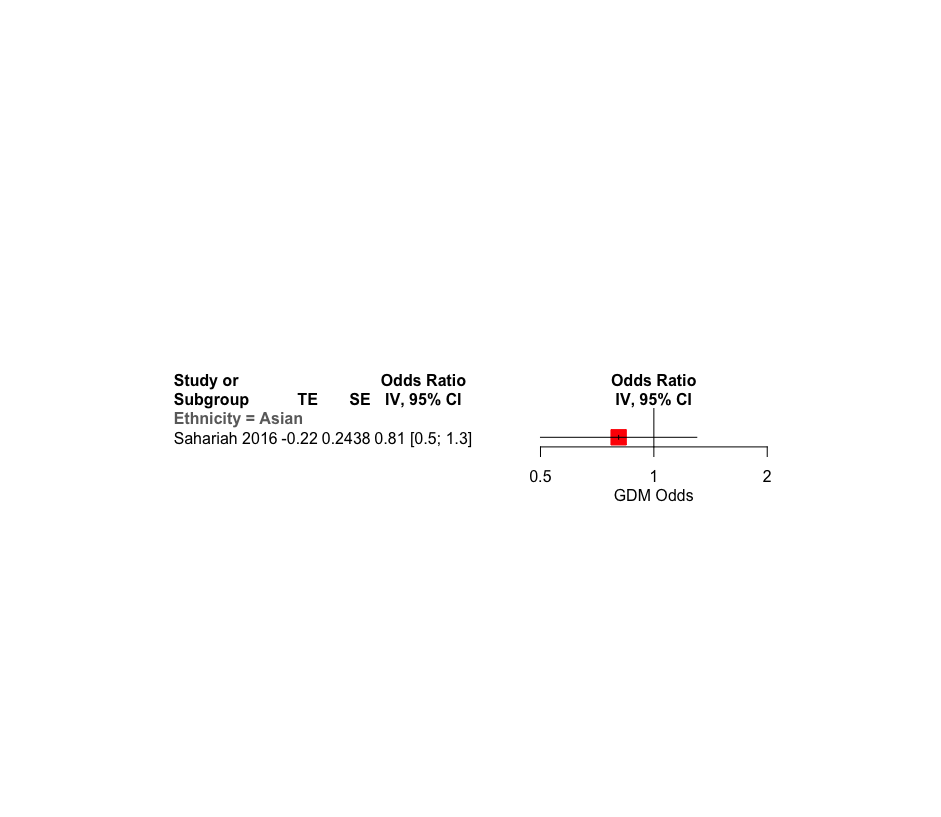

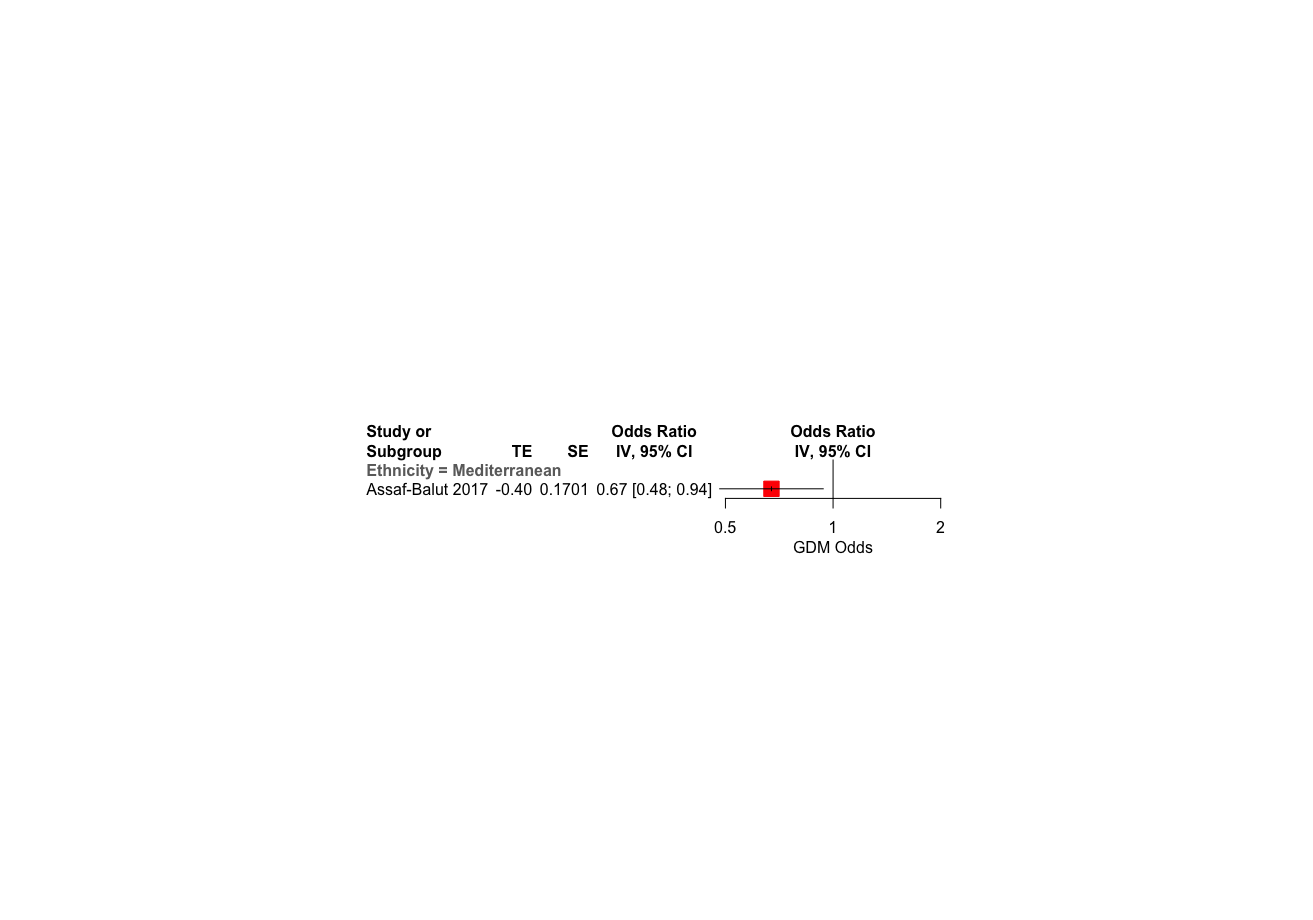


**C**

**B**

**A**

Fig D: Forest plots of Observational studies included within the ‘Healthy diet’ exposure***.***

Association*s* between *‘*healthy*’* dietary patterns and GDM in observational studies stratified by type of ‘healthy’ diet using a DerSimonian and Laird (DL) random effects meta-analysis: (A) Healthy recommendations. (B) Mediterranean diet, (C): Prudent diet, and (D) Plant-based pattern. TE: treatment effect, SE: standard error, IV: inverse variance method, CI: Confidence interval.**
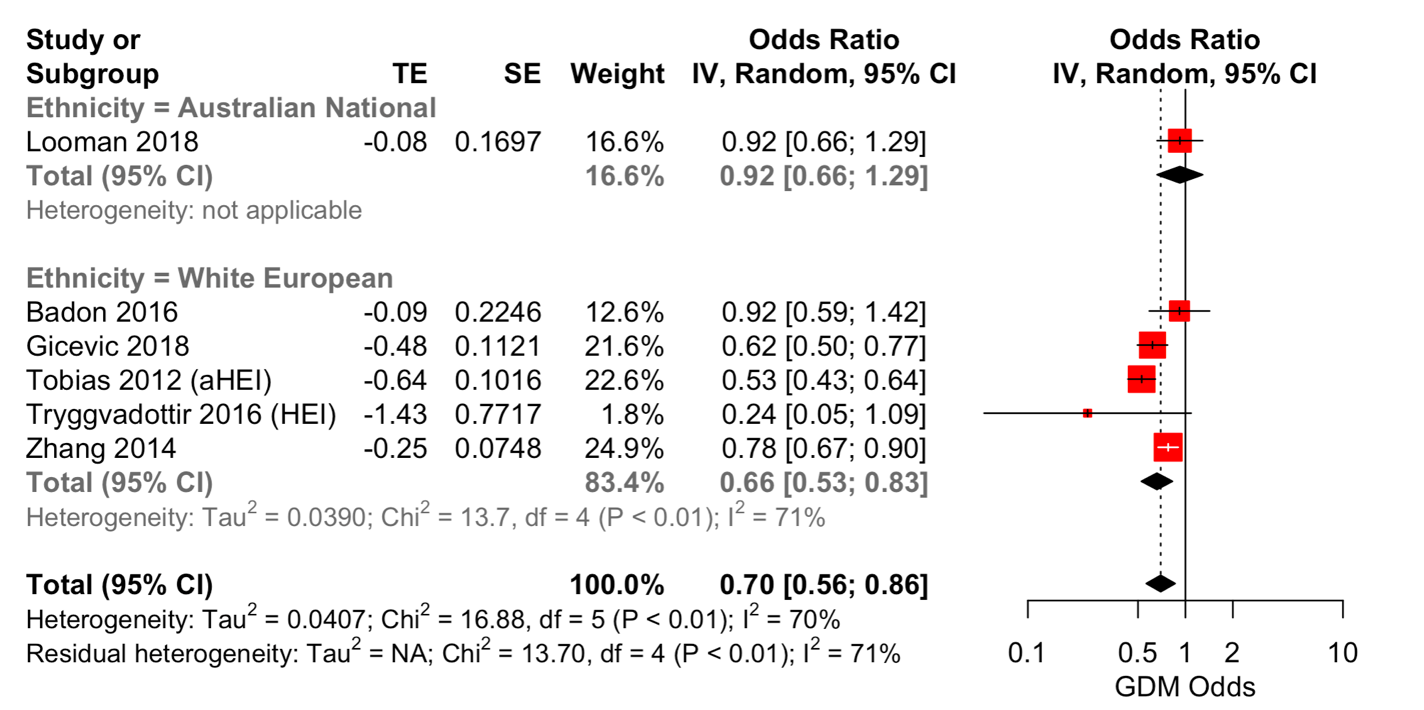
**
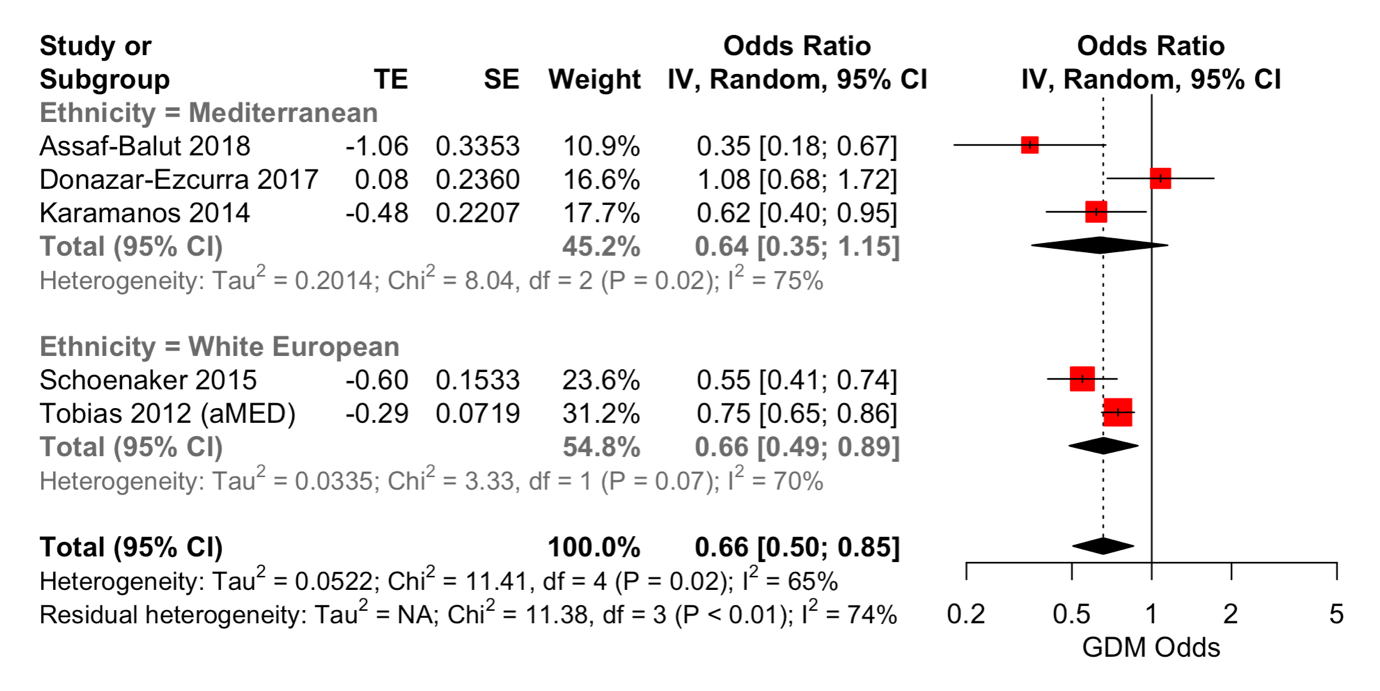


**B**

**A**

B

A

**C**

C


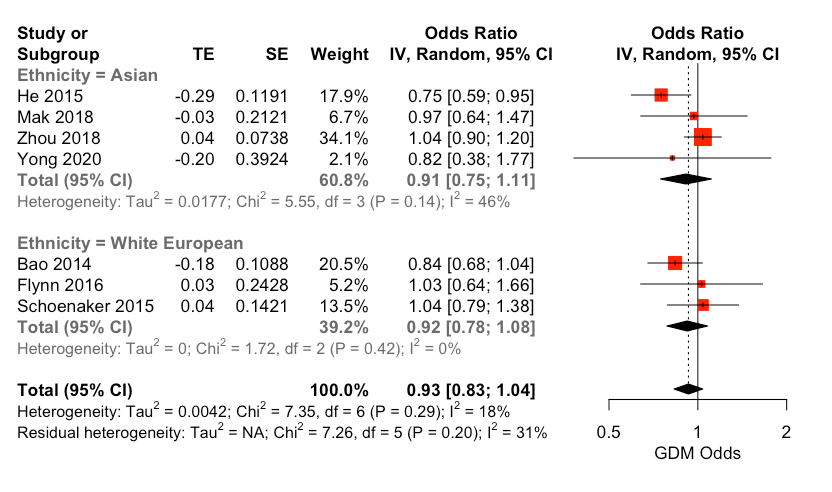

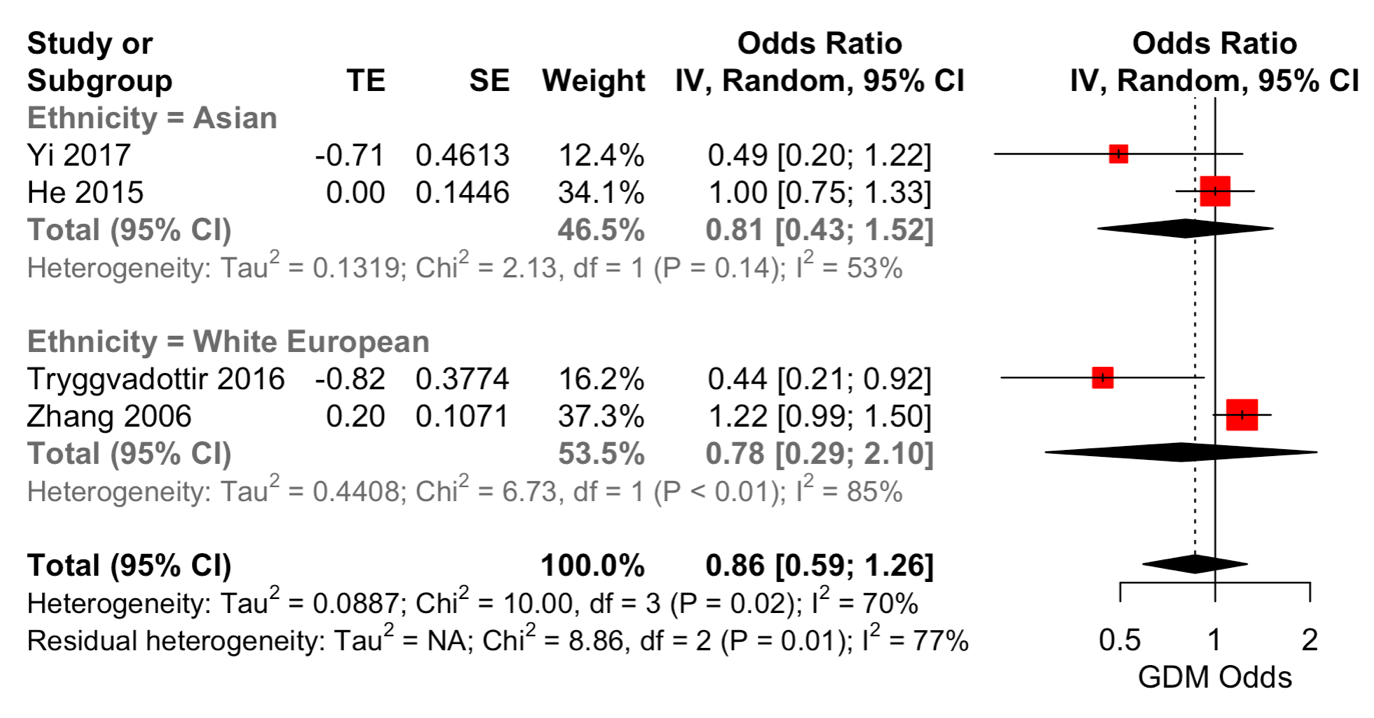


**D**

D

## Fig E: Forest plots of Observational studies included within the ‘Unhealthy diet’ exposure*.*

Association*s* between *‘*unhealthy*’* dietary patterns and GDM in observational studies stratified by ethnicity subgroups using a DerSimonian and Laird (DL) random effects meta-analysis: **A**: Western diet. **B** Fried/fast food. **C**: Sweets and Seafood pattern **D**: Unhealthy diets score


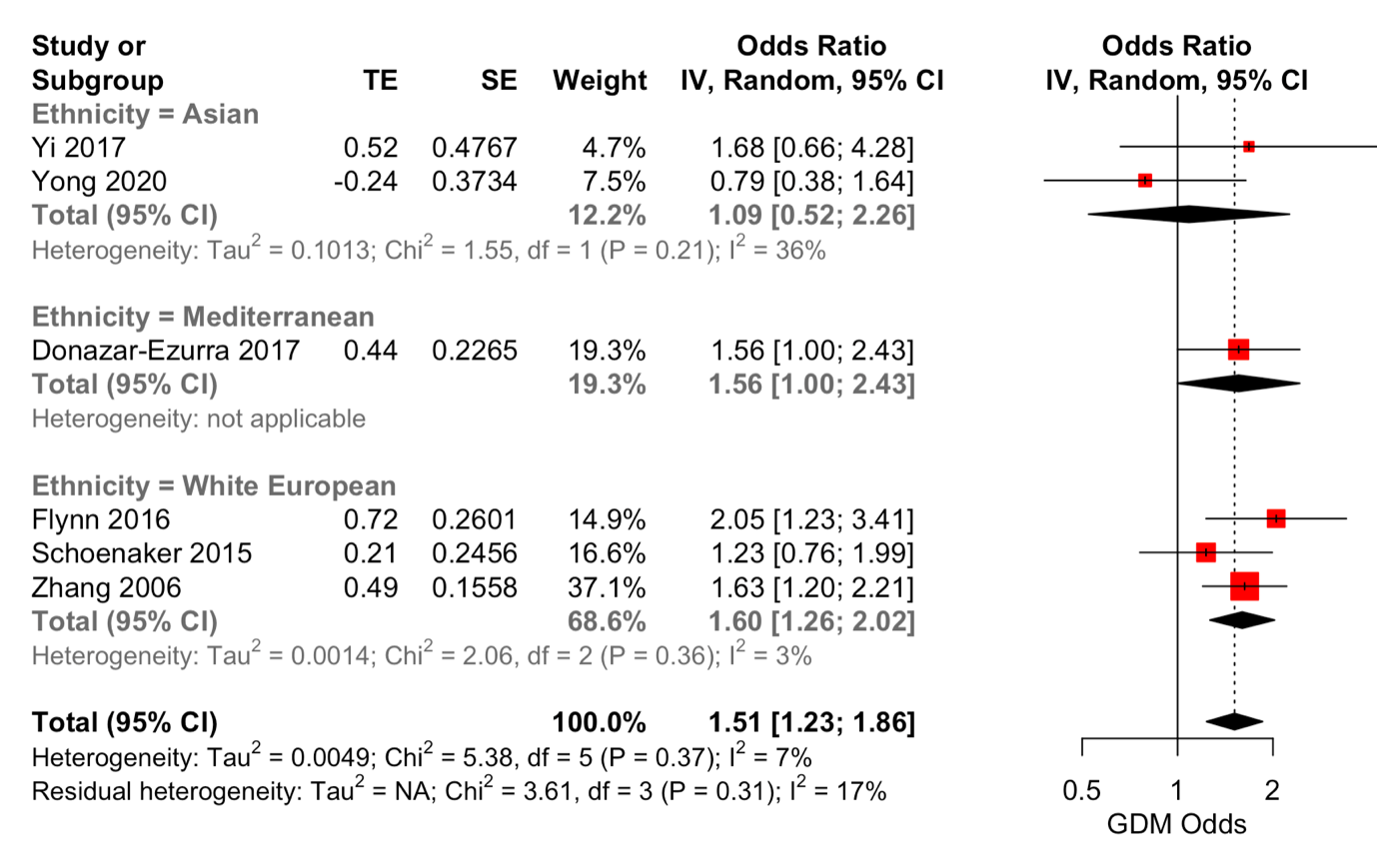


**B**

**A**

B

A


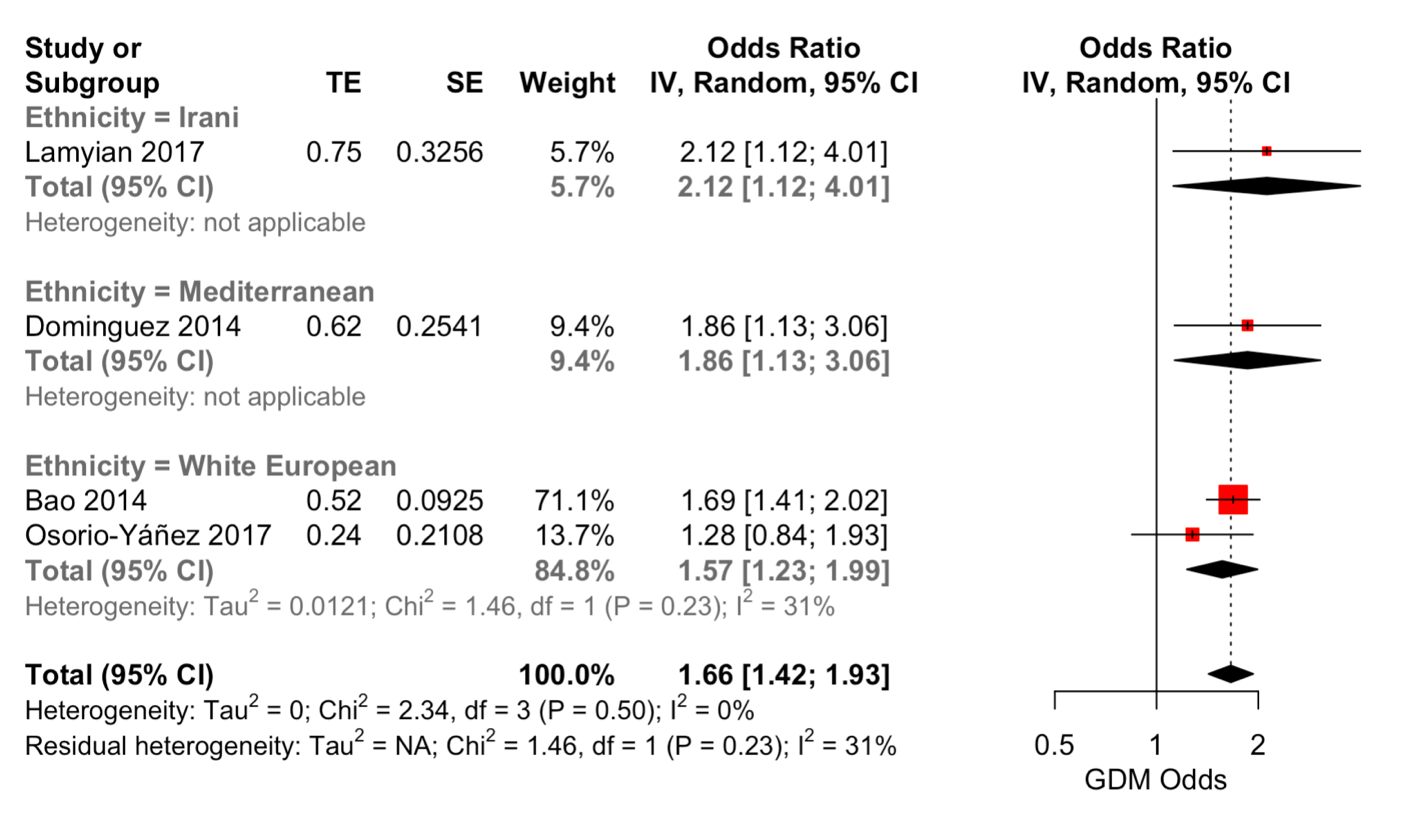


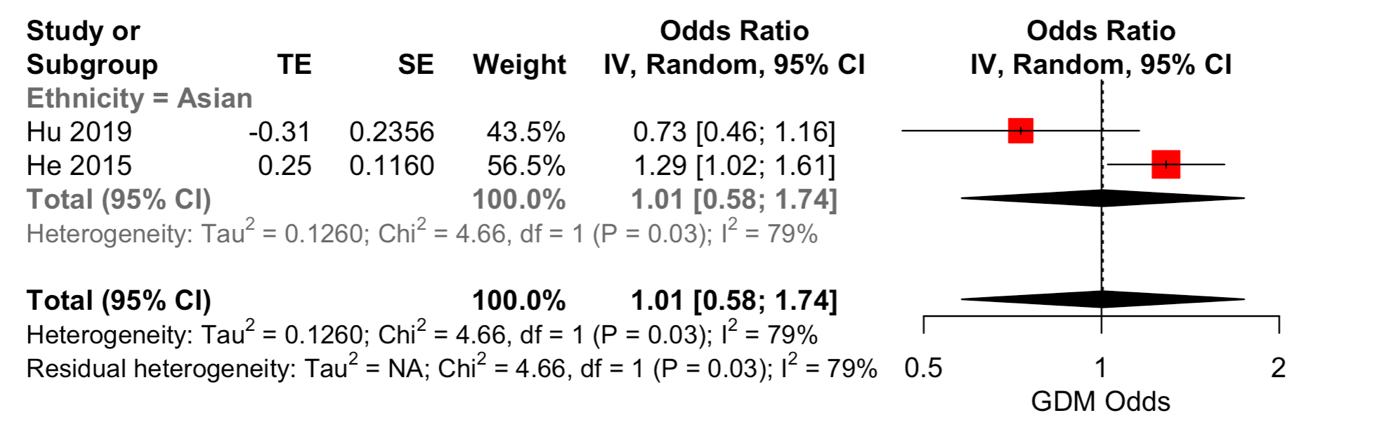


**D**

**C**

D

C


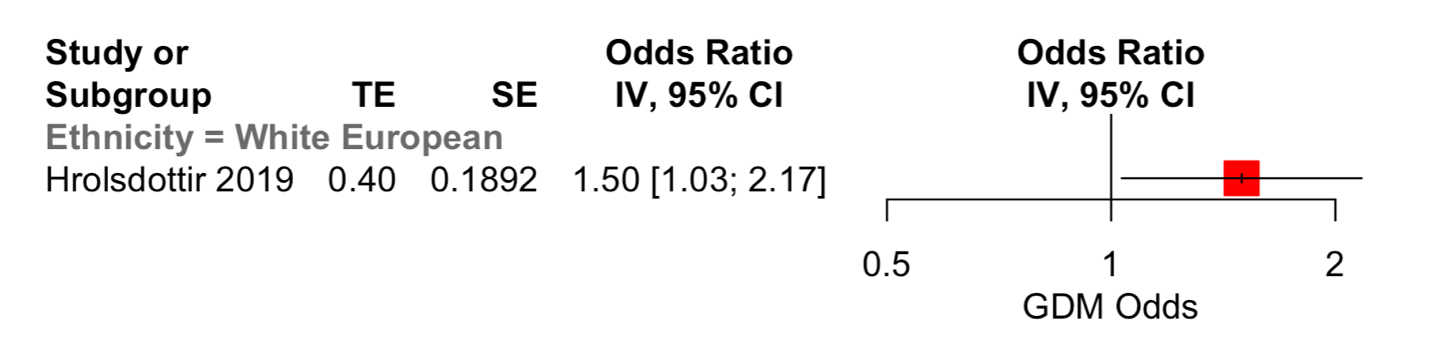


Fig F: Forest plots of Observational studies included within the ‘Unclassified diet’ exposures***.***


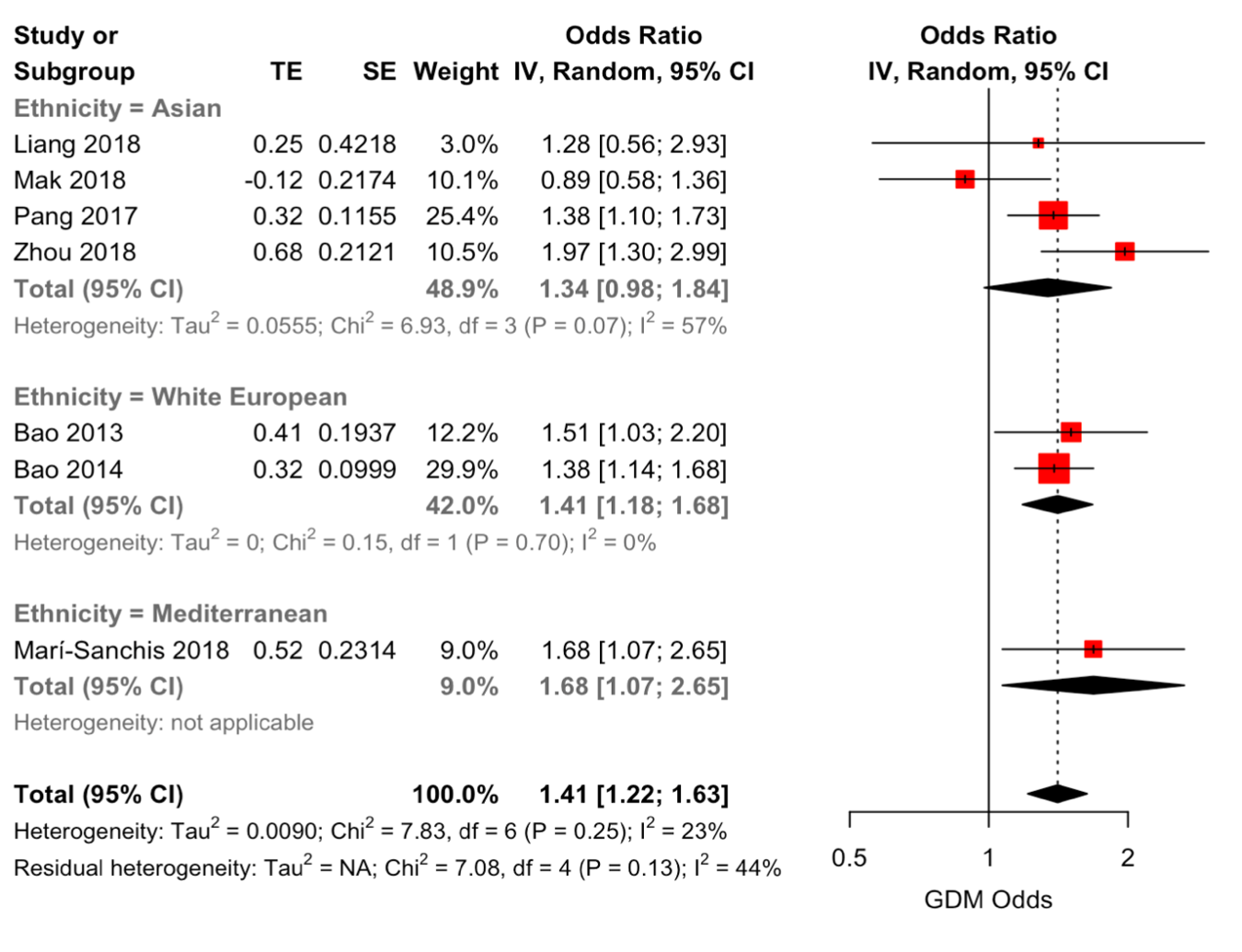
Association*s* between *‘*unclassified*’* dietary patterns and GDM in observational studies stratified ethnicity subgroups using a DerSimonian and Laird (DL) random effects meta-analysis: **A**: Meat pattern. **B** High-protein pattern. **C**: Fish **D**: Traditional Asian.

**A**

A


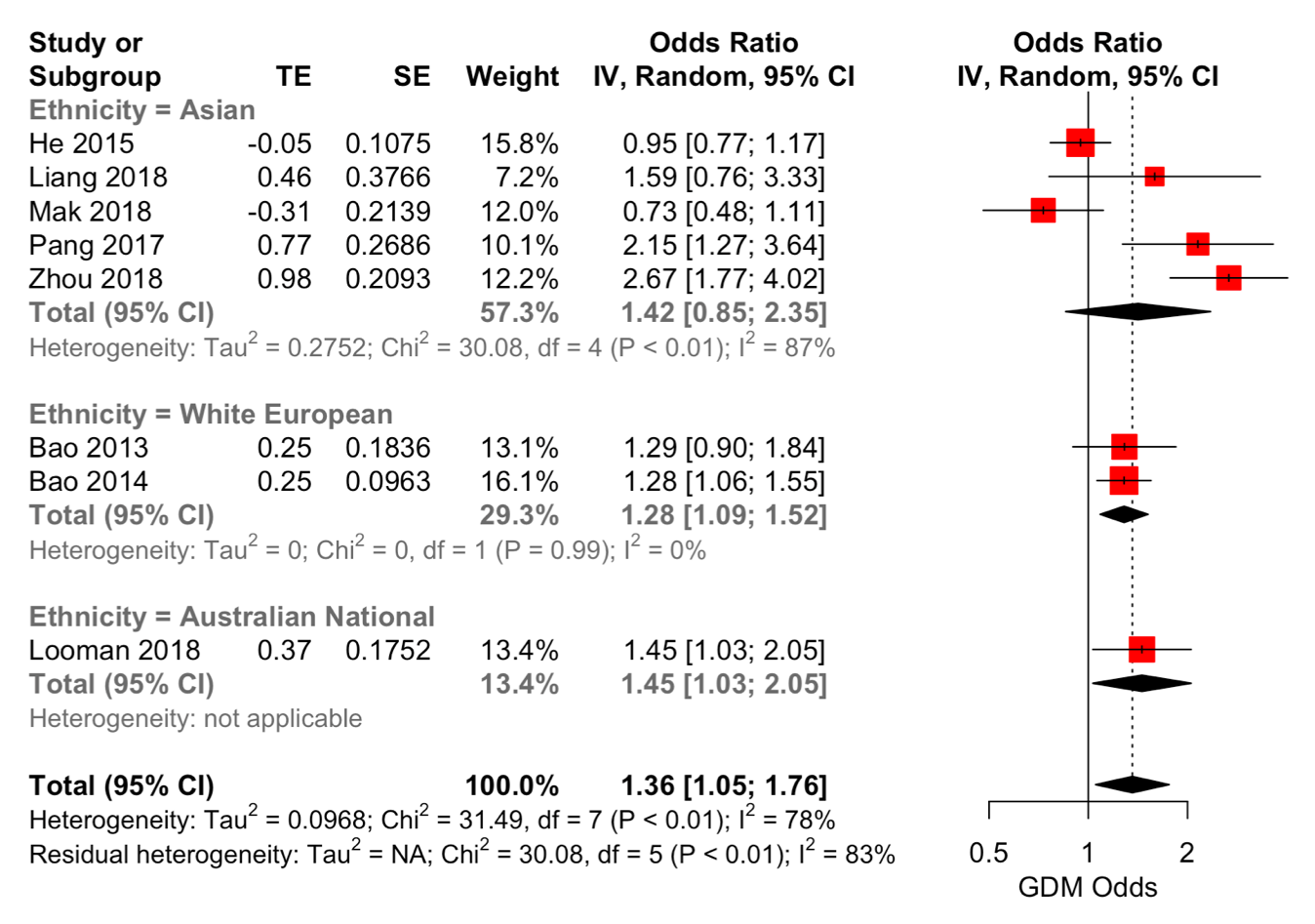


**B**

B


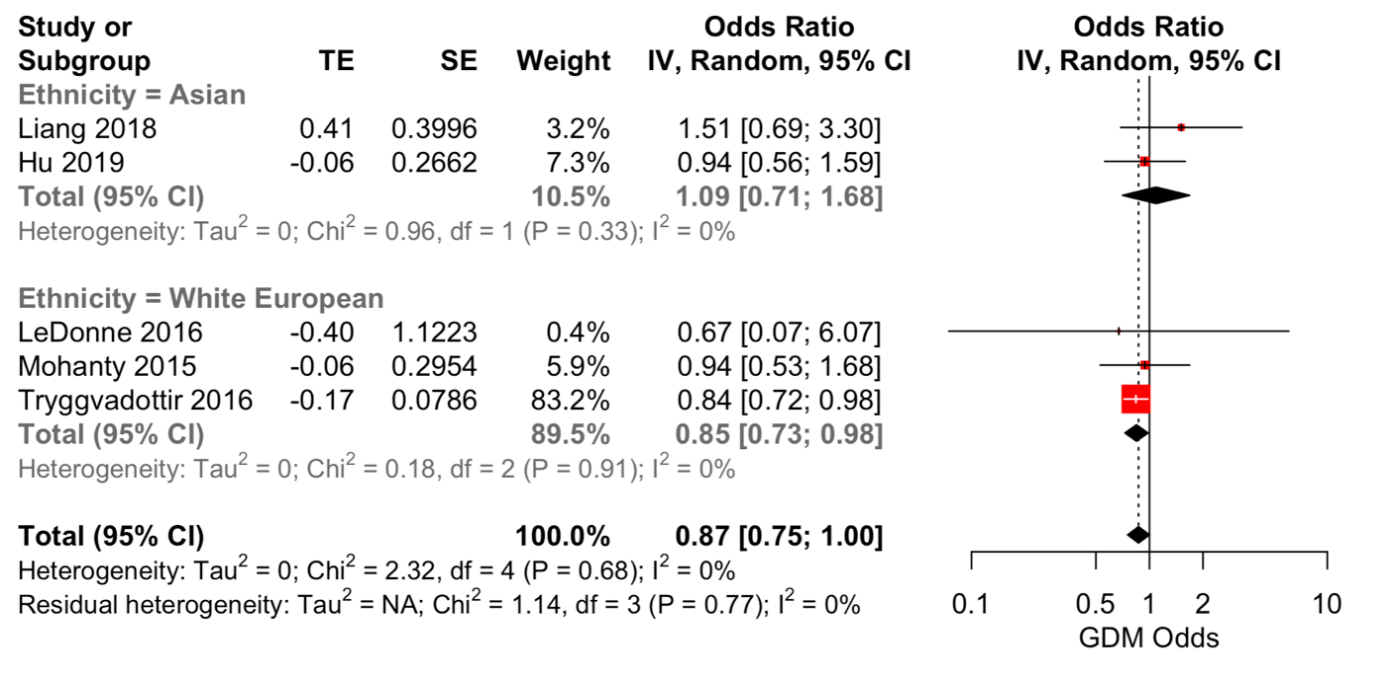


**C**

C

**D**

D


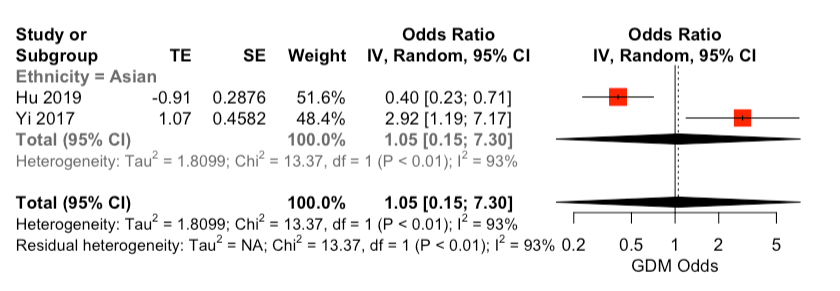


Fig G: Forest plots of Observational studies reporting a macronutrient-focussed diet***.***

Associations between macronutrient-defined diets and GDM in observational studies stratified by ethnicity subgroups using a DerSimonian and Laird (DL) random effects meta-analysis: **A**: Animal protein. **B** Vegetable protein. **C**: Carbohydrate **D**: Fat.


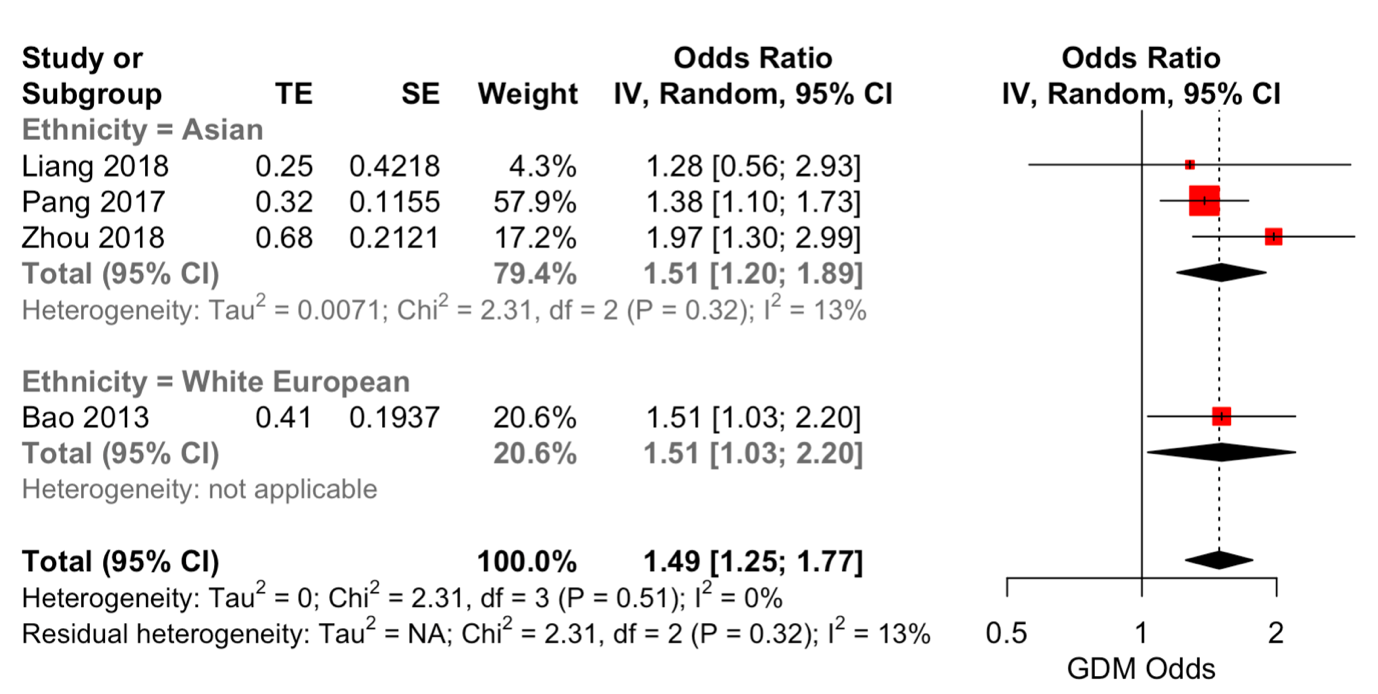


**A**

A

**B**

B


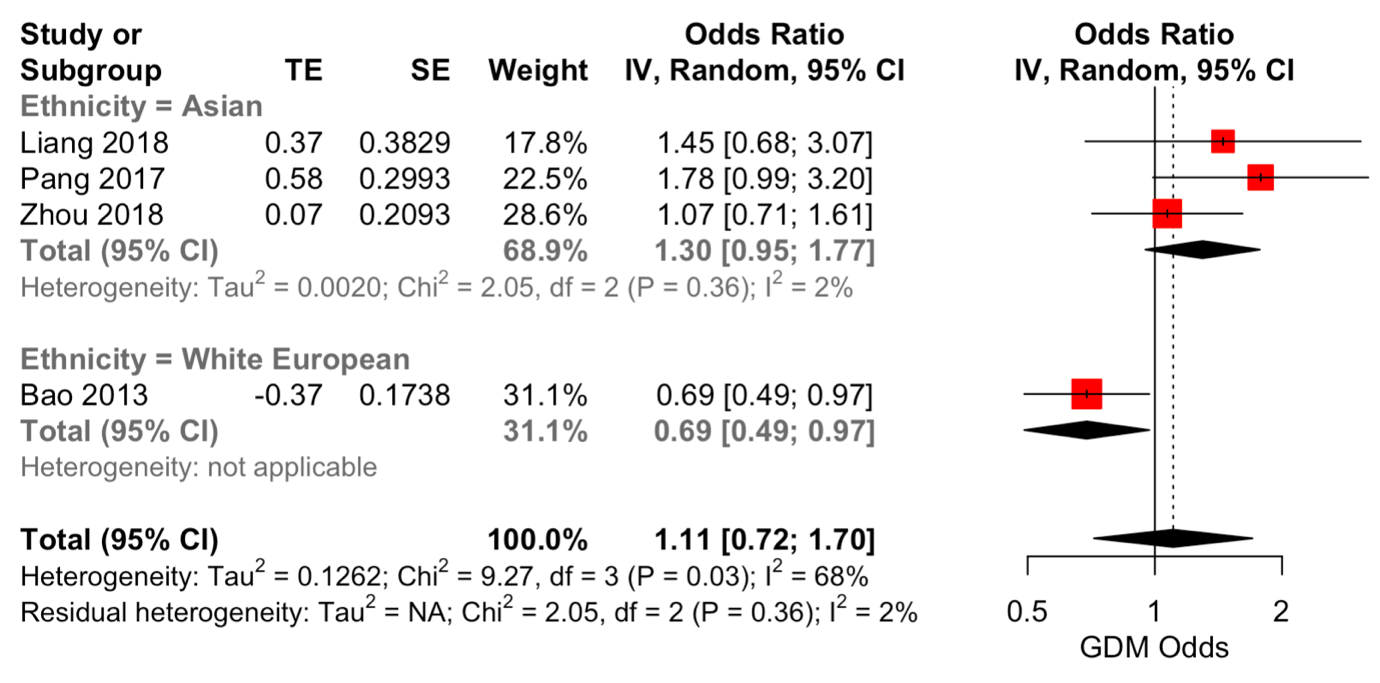


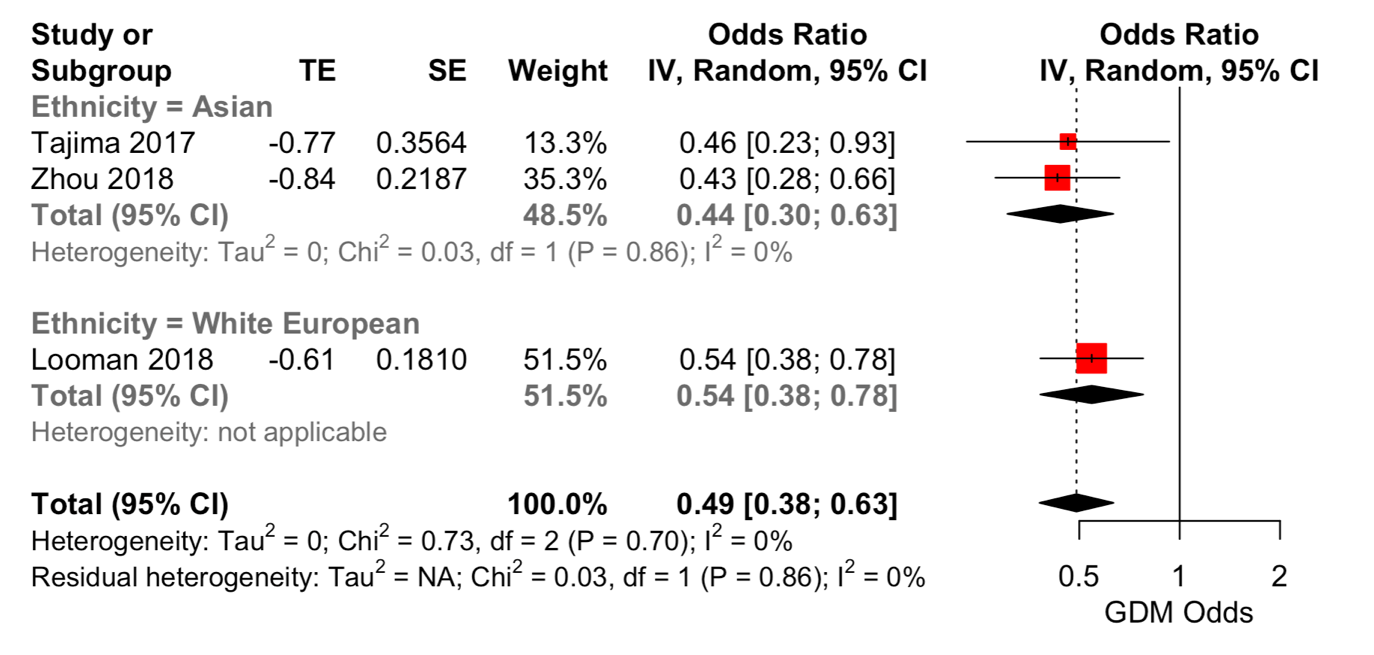


**C**

C


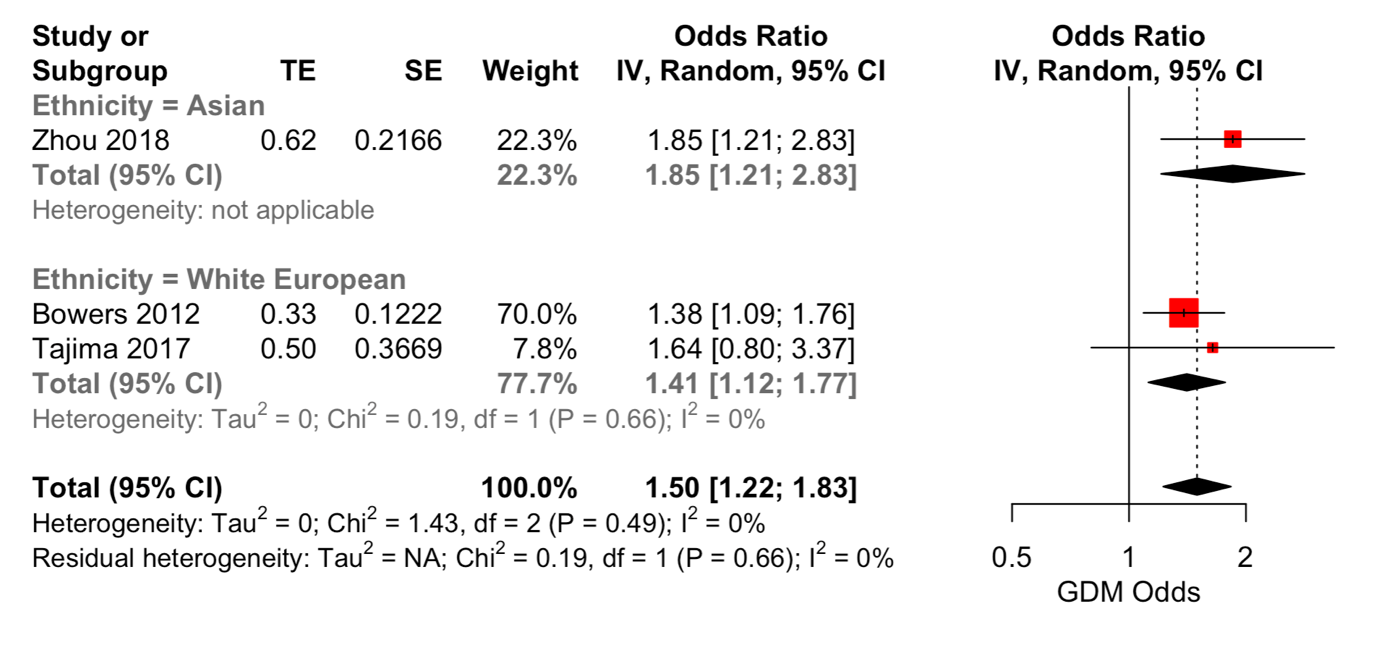


**D**

D

Fig H: Forest plots of combined analysis of RCTs and Observational studies**.**

Association*s* between ‘healthy’ diets and GDM stratified by type and ethnicity using a DerSimonian and Laird (DL) random effects meta-analysis: **A**: Overall ‘Healthy diet’ **B**: Healthy recommendations **C**: Mediterranean diet.


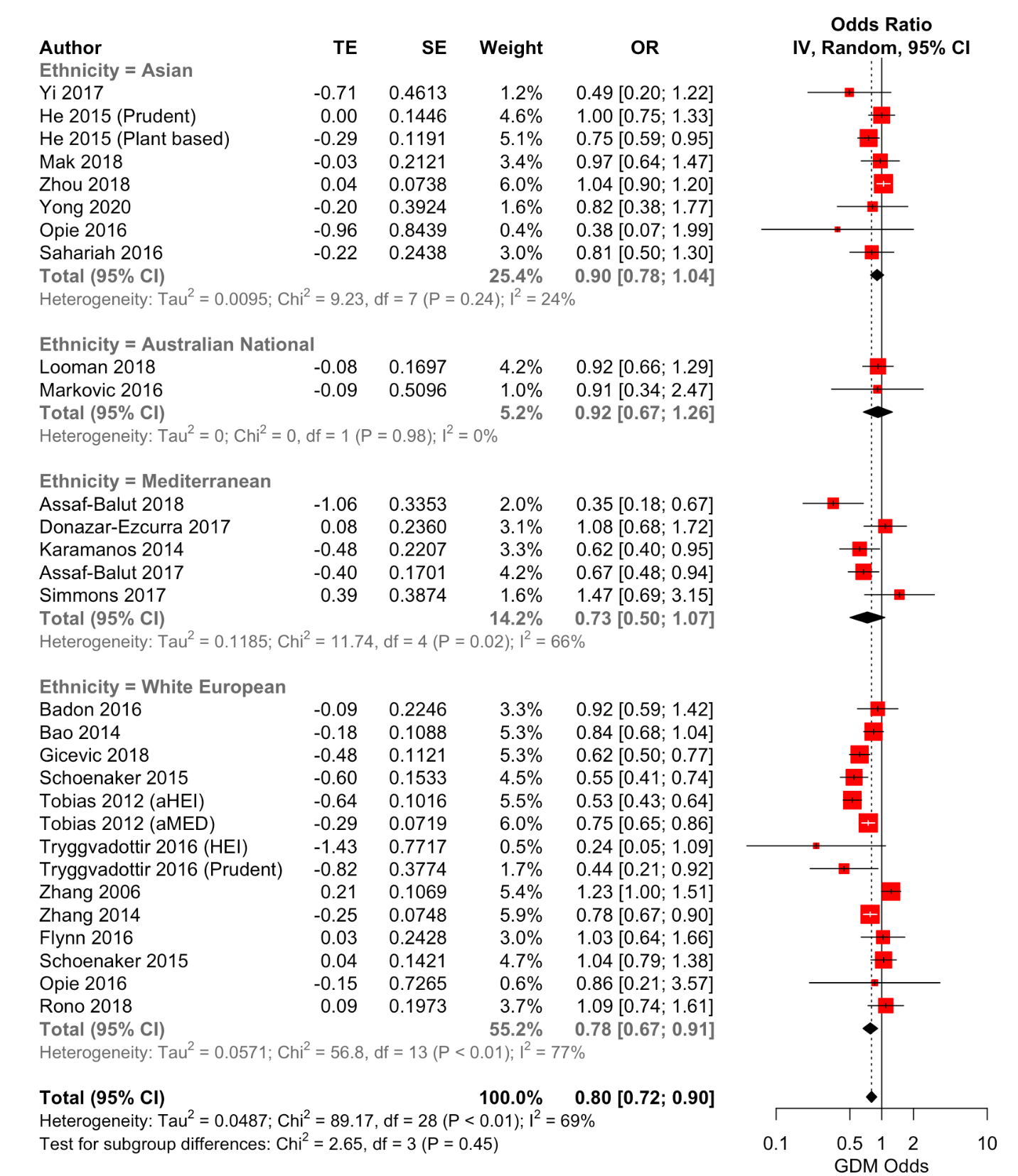


**A**

A


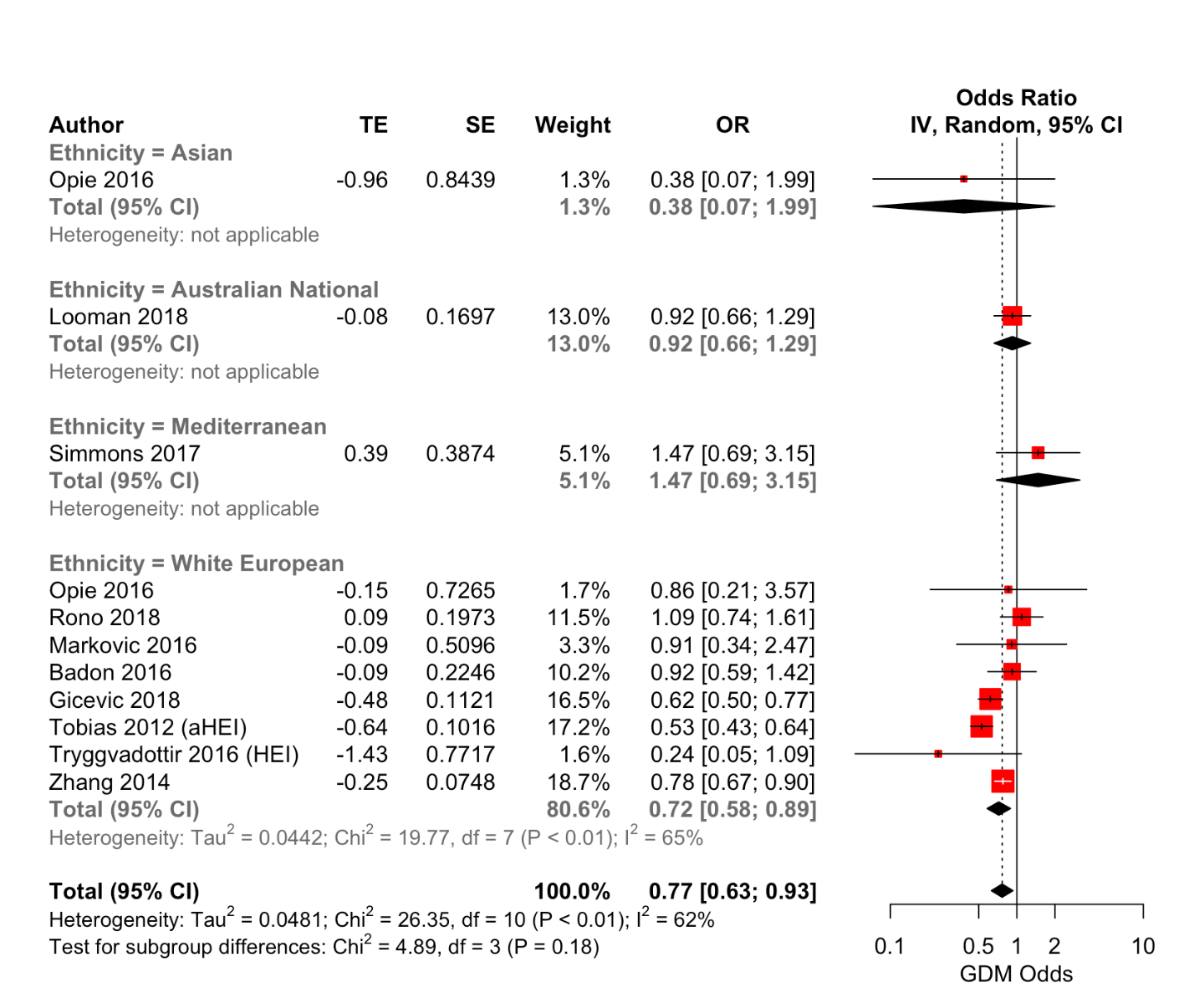

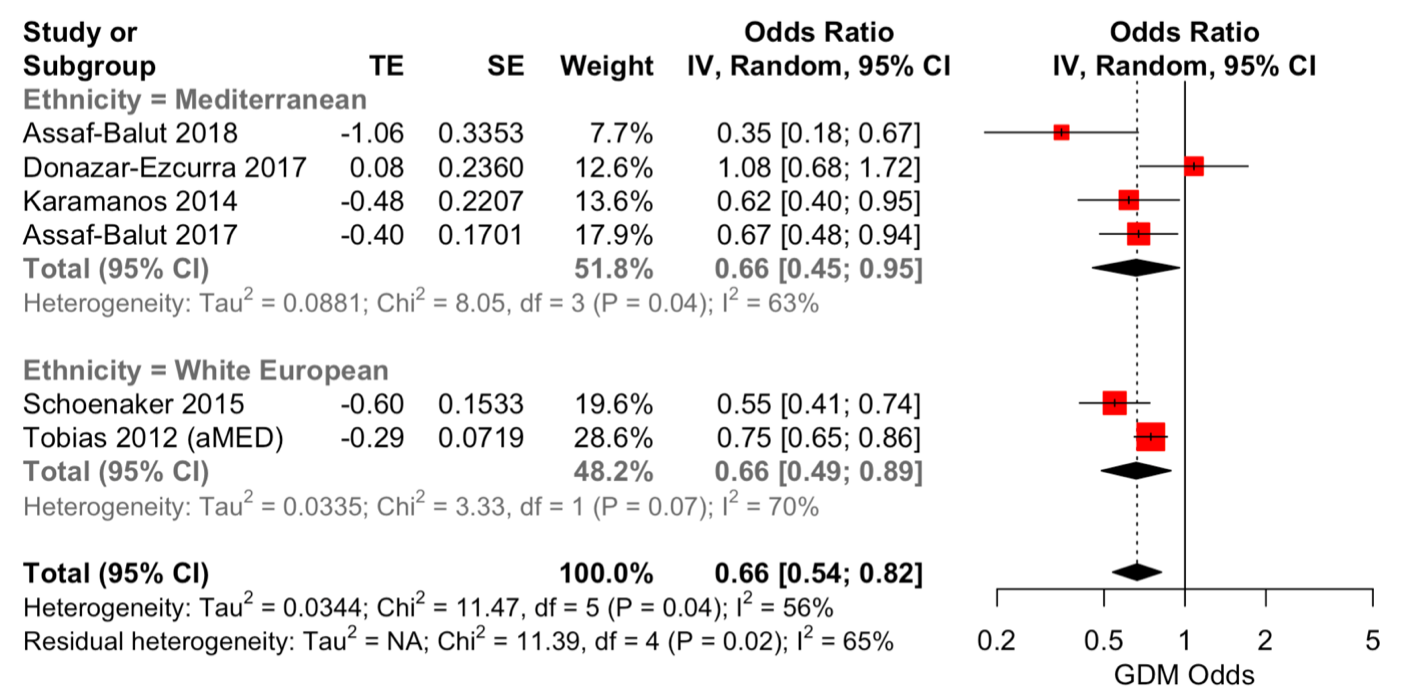


**C**

**B**

## Fig I: Risk of Bias scores for RCTs.

1-V10 correspond to the questions represented on the ADA tool. In brief, questions can be summarised as follows: V1- Clear research question, V2- Participant selection, V3- Comparable study groups, V4-Managing withdrawals, V5- Blinding, V6- Comparisons and exposures, V7- Outcome measurement, V8- Statistical analyse, V9- Conclusions and limitations, V10- Funding and sponsorship. **A**: Individual score for RCTs. **B**: Individual score for observational studies.


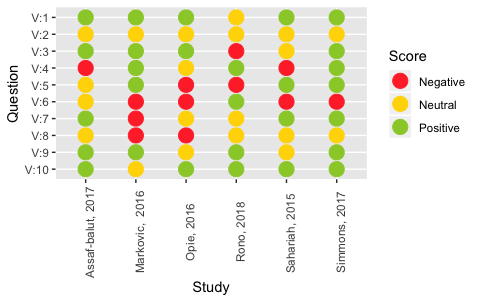


## Fig J: Risk of Bias scores for Observational studies.

1-V10 correspond to the questions represented on the ADA tool. In brief, questions can be summarised as follows: V1- Clear research question, V2- Participant selection, V3- Comparable study groups, V4-Managing withdrawals, V5- Blinding, V6- Comparisons and exposures, V7- Outcome measurement, V8- Statistical analyse, V9- Conclusions and limitations, V10- Funding and sponsorship. **A**: Individual score for RCTs. **B**: Individual score for observational studies.


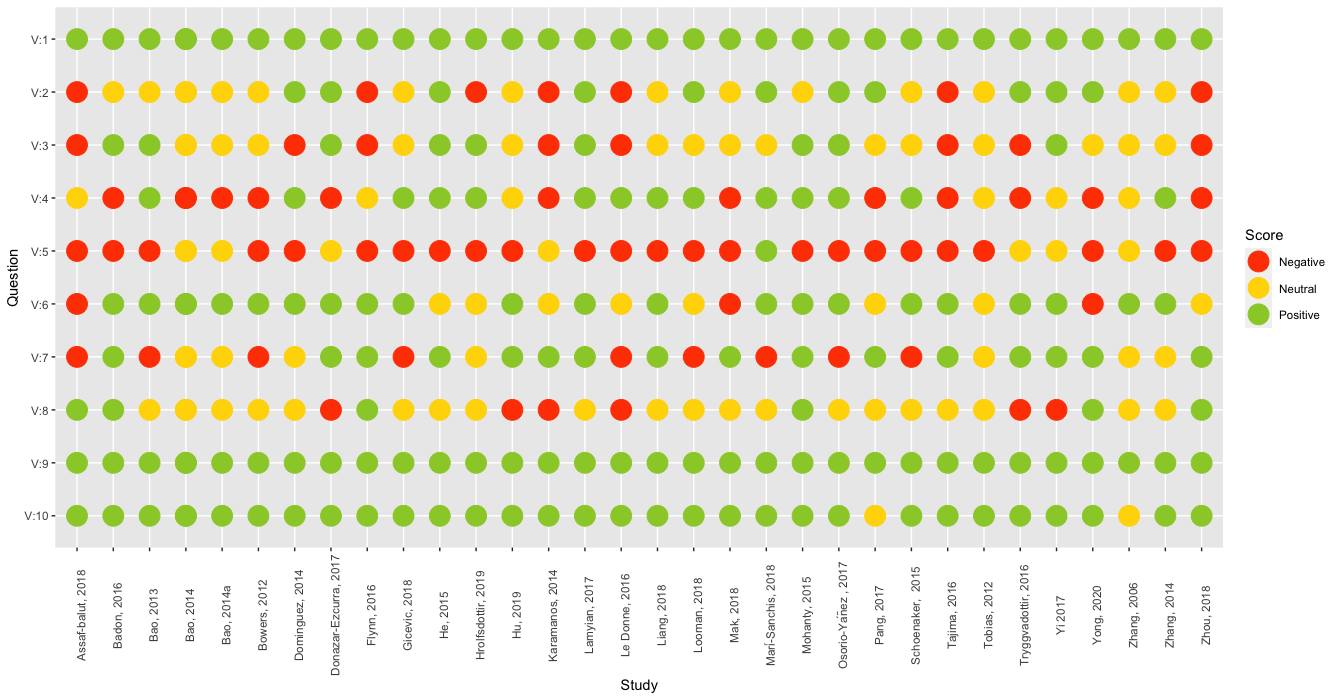


## Fig K: Risk of Bias results by question.

V1-V10 correspond to the questions represented on the ADA tool. In brief, questions can be summarised as follows: V1- Clear research question, V2- Participant selection, V3- Comparable study groups, V4-Managing withdrawals, V5- Blinding, V6- Comparisons and exposures, V7- Outcome measurement, V8- Statistical analyse, V9- Conclusions and limitations, V10- Funding and sponsorship. **A**: Score breakdown for RCTs. **B**: Score breakdown for observational studies. **C:** Score breakdown for combined analyses.


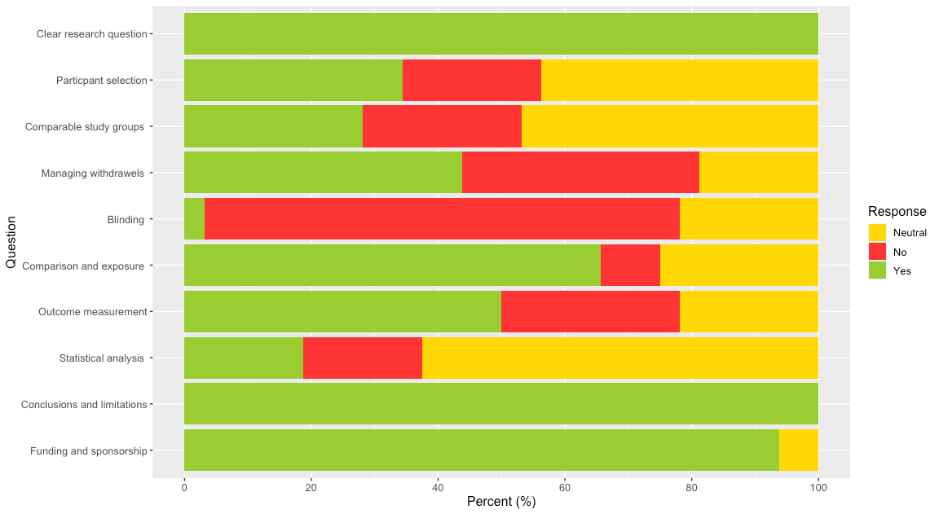


**C**

**A**

C

A


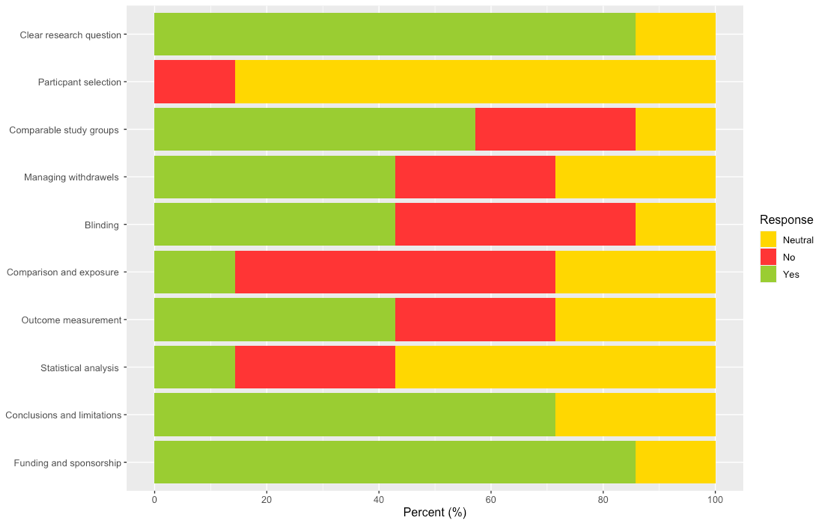


**B**

**B**


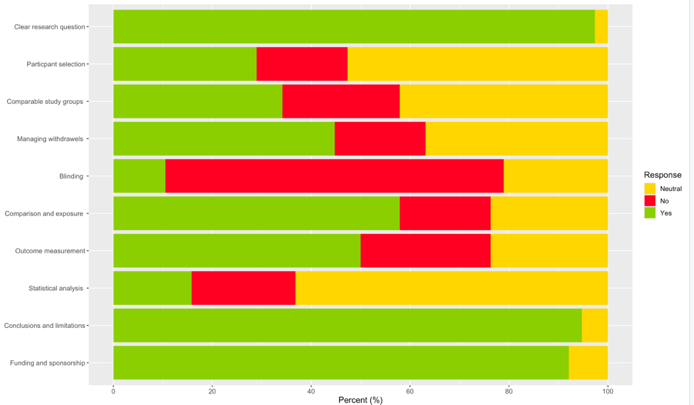


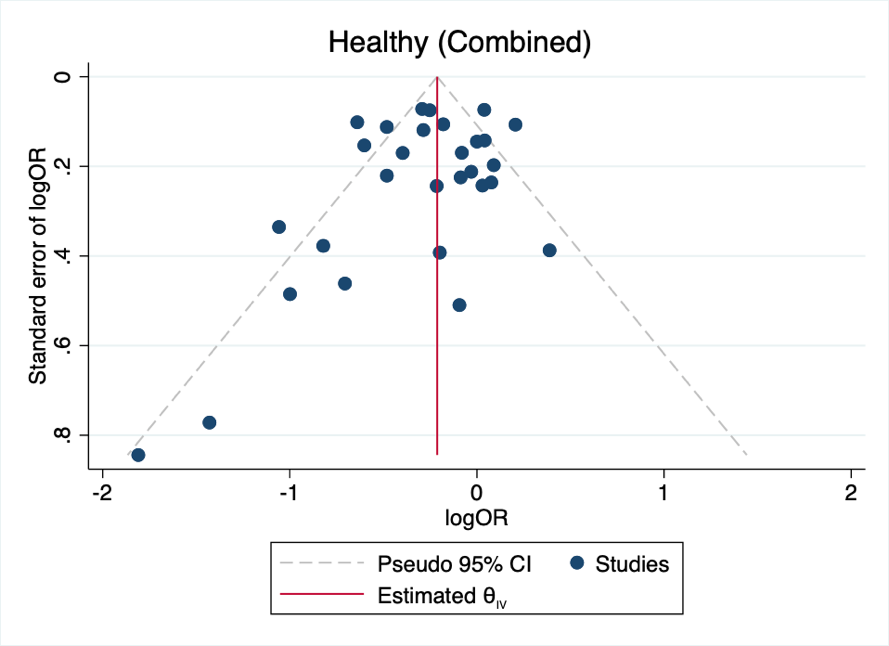

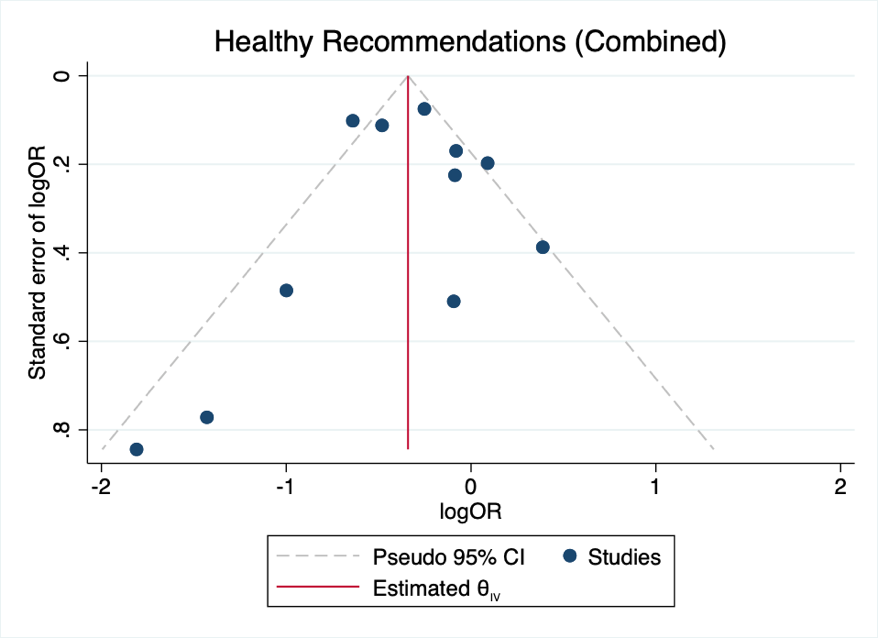

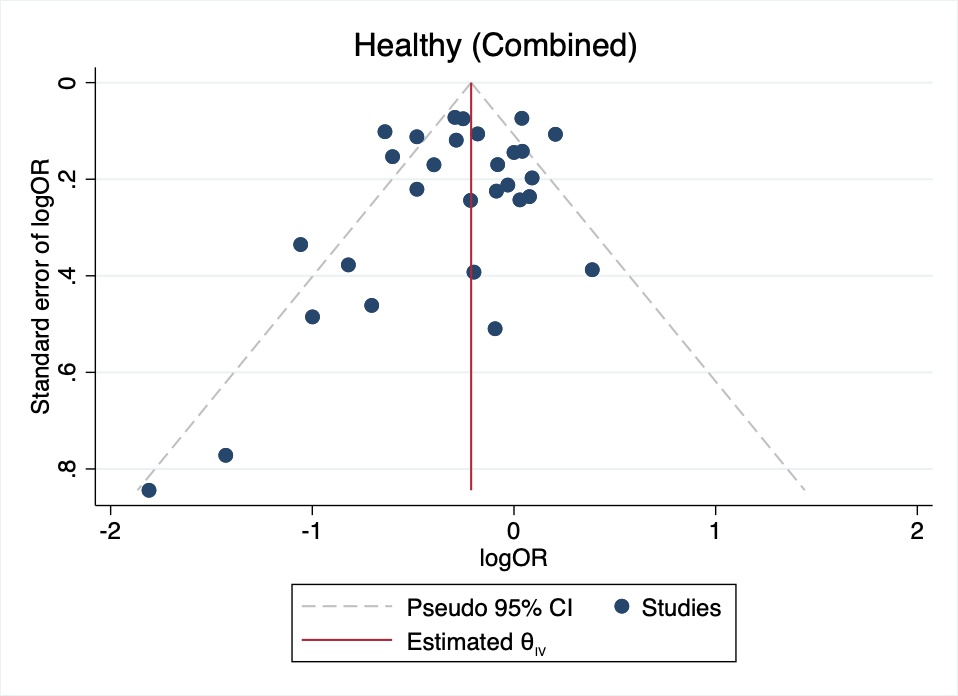

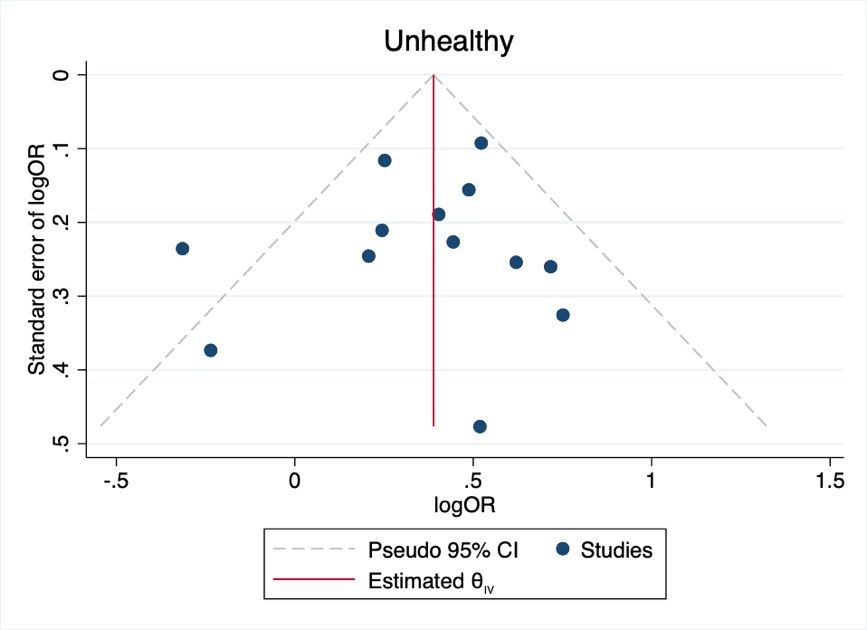

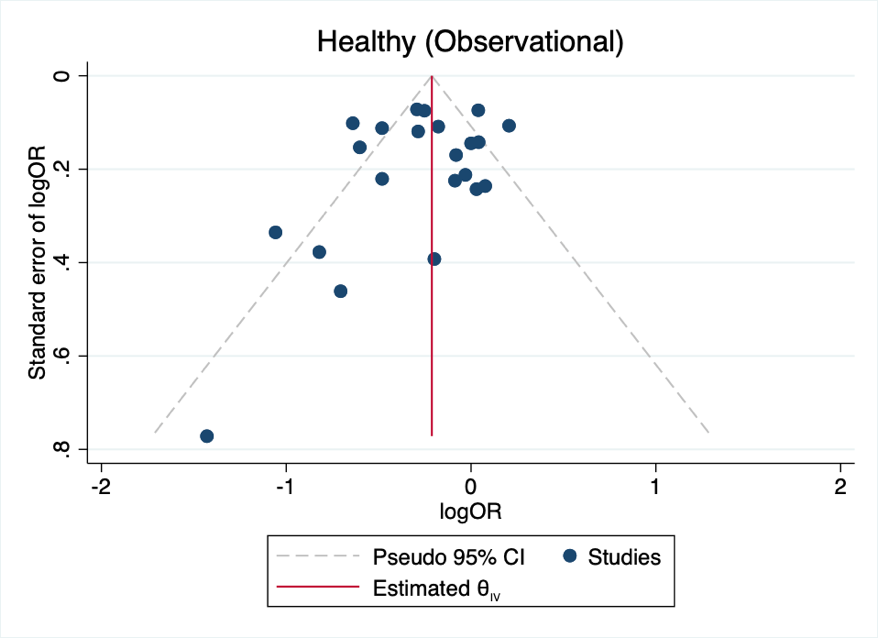
Fig L: Assessment of publication bias.

Publication bias was assessable for an exposure when n >= 10.

# SUPPLEMENTARY TABLES

## Table A - Summary of RCTs included in analysis.

^1^I/C, intervention/control, 2 UK, Ireland, Netherlands, Austria, Poland, Italy, Spain, Denmark, Belgium, GI- Glycaemic Index. OGTT- Oral Glucose Tolerance Test.

| **Study** | **Grouped Exposure** | **Exposure** | **Country** | **Ethnicity of participants** | **GDM**  **Diagnostic**  **Critera** | **Sample**  **size**  **(I/C)**^1^ | **Pre/ During pregnancy** | **Initation (I) and Duration (D)** | **Intervention** | **Control** |
| --- | --- | --- | --- | --- | --- | --- | --- | --- | --- | --- |
| Assaf-balut, 2017 [1] | Healthy | Mediterranean diet | Spain | Mediterranean | 75g OGTT,  ≥1 of the following:  Fasting  ≥5.1 mmol/L  1-hr ≥10 mmol/L  2-hr ≥8.5mmol/L | 874 (434/440) | During Pregnancy | **I:** 12^th^ week gestation  **D:** 24-26 weeks’ gestation | 1. 3 servings/day vegetables, fruit (not including juices), skimmed dairy, and wholegrains 2. 2-3 servings/week legumes and fish consumption 3. Limited red meat, refined sugars, and convenience/ processed foods 4. Supplemented with olive oil and pistachios | Usual standard of care |
| Markovic, 2016 [2] | Healthy | Healthy diet recommendations | Australia | Australian Nationals | 75g OGTT  Fasting ≥ 5.5 mmol/L  1-hr ≥ 10 mmol/L  2-hr ≥ 8 mmol/L | 125  (65/60) | During Pregnancy | **I**: 14-20 weeks’ gestation  **D**: Not reported | 1. Low GI diet | High fibre, moderate-GI diet |
| Opie, 2016 [3] | Healthy | Healthy diet recommendations | Australia | White European | 75g OGTT Fasting  ≥ 5.5mmol/L  2-hr ≥8 mmol/L | 153  (82/71) | During Pregnancy | **I**: 24-28 weeks’ gestation  **D**: Not reported | 1. AGHE modified for pregnancy | Usual standard of care |
|  |  |  |  | Asian |  | 58  (10/48) |  |  |  |  |
| Rono, 2018 [4] | Healthy | Healthy diet recommendations | Finland | White European | 75g OGTT  Fasting  ≥5.3 mmol/L  1-hr ≥10 mmol/L  2-hr ≥8.6 mmol/L | 335  (235/100) | During pregnancy | **I:** <20 weeks’ gestation  **D:** Until OGTT test based upon | 1. Structured and individually modified dietary advice based on the Nordic nutritional recommendations | Usual standard of care |
| Sahariah, 2016 [5] | Healthy | Healthy  Snack | India | Asian | 75g OGTT,≥1 of the following:  Fasting  ≥ 5.1 mmol/L  2-hr > 8.5mmol/L | 1008  (492/516) | Pre and During pregnancy | **I:** ≥ 90 days pre- pregnancy  **D:** Until delivery | 1. Supplementation with leafy green vegetables, fruit and milk | Snacks made of low macro-nutrient vegetables including potatoes and onions |
| Simmons,  2017 [6] | Healthy | Healthy recommendations | European countries | Mediterranean | 75g OGTT,≥1 of the following:  Fasting  ≥ 5.1 mmol/L  2-hr > 8.5mmol/L | 206  (106/100) | During pregnancy | **I:** <20 weeks’ gestation  **D:** Until delivery | 1. Lower simple/complex carbohydrate and fat intake 2. Higher fibre and protein intake. Calorie deficit with an empathies on portion control | Usual standard of care |

Table B - Summary of observational studies included in analysis.

EVOO, extra virgin olive oil; FFQ, food frequency questionnaire; MUFA, monounsaturated fatty acids; PUFA, polyunsaturated fatty acids; SFA, saturated fatty acids; SSB, sugar sweetened beverages; AHEI, alternative healthy eating index; TE, total energy; OGTT, oral glucose tolerance test. ^†^ Number within each tertile (3rd vs 1st) calculated as a third of the included study population.^††^ Number within each quartile (4th vs 1st) calculated as a quarter of the included study population.

| **Study** | **Exposure** | **Grouped exposure** | **Country of study** | **Ethnicity of participants** | **GDM**  **Diagnostic**  **Critera** | **Sample Size**  **(High /low consumers)** | **Diet Pattern** | **Method of dietary assessment** | **Timing of assessment** |
| --- | --- | --- | --- | --- | --- | --- | --- | --- | --- |
| Assaf-Balut, 2018[1] | Mediterranean diet | Healthy | Spain | Mediterranean | Not stated | 759  (623/136) | Frequent consumption of vegetables, legumes, fruits, nuts, EVOO, oily fish, canned fish, wholegrains, cereals, pasta and skimmed dairy products | FFQ | During pregnancy: 12-14 weeks |
| Badon, 2016[7] | Healthy diet recommendations | Healthy | USA | white European | 100g OGTT  ≥2 of the following:  Fasting ≥ 105 mg/dL.  1-hr ≥190 mg/dL  2-hr ≥165 mg/dL  3-hr ≥ 145 mg/dL | 3305  (611/2694) | 1. Modified version of AHEI-2010 2. Increased consumption of vegetables, fruit, whole grains, nuts, long- chain (n-3) fatty acids, and PUFAs 3. Limited intake of SSBs, red/processed meat, trans-fat and sodium | FFQ- Diet over the previous year | During pregnancy:  15 weeks, +/- 3 weeks) |
| Bao, 2013 [8] | High-protein diet | - | USA | white European | Self reported | 5799  (2793/3006) | 1. Individuals total protein intake, split into quintiles based on population (%TE) | FFQ every 4 years | Prior to pregnancy: 2001 |
|  | Vegetable protein | - |  |  |  | 6159  (2871/3288) | 1. Individuals vegetable protein intake, split into quintiles based on population intake (%TE) |  |  |
|  | Meat pattern/ Animal protein | - |  |  |  | 5877  (2862/3015) | 1. Individuals animal protein intake, split into quintiles based on population intake. (%TE) |  |  |
| Bao, 2014 [9] | Plant-based pattern | Healthy | USA | white European | Self reported | 10860  (3685/7175) | 1. Determined on the basis of percentage of energy from carbohydrate, vegetable protein and vegetable fat 2. Higher score reflects a higher intake of vegetable protein/ fat and a lower intake of carbohydrate | FFQ – Diet over the previous day (x4) | Prior to pregnancy:  2001 |
|  | Meat pattern | - |  |  |  | 10301  (4642/5659) | 1. Determined on the basis of percentage of energy from carbohydrate, total protein and total fat 2. Higher score reflects a higher intake of protein/ fat and a lower intake of carbohydrate |  |  |
|  | High Protein | - |  |  |  | 10864  (4591/6273) | 1. Determined on the basis of percentage of energy from carbohydrate, animal protein and animal fat 2. Higher score reflects a higher intake of animal protein/ fat and a lower intake of carbohydrate |  |  |
| Bao, 2014^2^ [10] | Fried/ fast food | Unhealthy | USA | white European | Self reported | 10866  (348/10518) | 1. Frequent consumption of fast food | FFQ every 4 years | Prior to pregnancy:  2001 |
| Bowers, 2012[11] | Fat | - | USA | white European | Self repoted – no information on the critera used | 6020  (2190/3110) | 1. Total fat (%TE) | FFQ every 2 years – Diet over the previous year | Pre-pregnancy:  Most recent questionaries |
| Dominguez, 2014[12] | Fried/ fast food | Unhealthy | Spain | Mediterranean | Self reported and then confirmed via a panel of medical doctors. Most common criterea used National Diabetes Group Criterea and Carpenter and Coustan cut-offs | 1587  (971/616) | 1. Frequent consumption of hamburgers, sausages and pizza | FFQ every 2 years | Pre-pregnancy:  Most recent questionnaire |
| Donazar-Ezcurra, 2017[13] | Mediterranean diet | Healthy | Spain | Mediterranean | Not stated | 1727  (863/864) | 1. High intake of poultry, olive oil, nuts, low-fat dairy products, whole grain bread, fish, fruit and vegetables | FFQ- Diet over the previous 24 hours | Pre-pregnancy:  Most recent questionarie |
|  | Western diet | Unhealthy |  |  |  | 1727  (863/864) | 1. Frequent intake of high-fat processed meats, potatoes, commercial bakery goods, whole dairy products, fast food, sauces, pre-cooked foods, SSB's and confectionary |  |  |
| Flynn, 2016[14] | Western diet | Unhealthy | UK | white European | 75g OGTT  ≥1 of the following:  Fasting ≥ 5.1 mmol/L  1-hr ≥ 10 mmol/L  2-hr > 8.5 mmol/L | 425  (206/219) | 1. High intake of potato, French fries, crisps, processed meat, fizzy drinks (SSB's and sugar free), root vegetables, green vegetables and chocolate | FFQ – Diet over the previous week | During pregnancy:  15-18 weeks until 27-28 weeks |
|  | Plant-based pattern | Healthy |  |  |  | 429  (213/216) | 1. Frequent intake of fresh/citrus/tropical fruits, green/root/salad vegetables, bananas and yoghurt |  |  |
| Gicevic 2018[15] | Healthy diet recommendations | Healthy | USA | white European | Self reported – No information on most common critea used | 7274  (4206/3068) | 1. AHEI 2010 adherence score. 2. Characterised by high intakes of nuts, long chain omega-3 fats, polyunsaturated acids, nuts and low intakes of red meat, refined sugar, refined grains and SSBs | FFQ- Diet over the previous year | Pre-pregnancy:  Most recent dietary assessment  (1991-2001) |
| He, 2015[16] | Prudent diet | Healthy | China | Asian | 75g OGTT  ≥1 of the following:  Fasting ≥ 5.1 mmol/L  1-hr ≥ 10 mmol/L  2-hr > 8.5 mmol/L | 369  (188/181) | 1. Frequent intake of dairy products, nuts, eggs, fish, soups and fruits. 2. Infrequent intake of processed meats, SSBs, and processed vegetables | FFQ- Diet over the previous week (x3) | During pregnancy:  Week 16,  Weeks 24-27,  Weeks 35-38 |
|  | Plant-based pattern | Healthy |  |  |  | 2040  (1019/1021) | 1. Frequent intake of, beans, mushroom, melon vegetables, seaweed, legumes, fruits, leafy/root/cruciferous vegetables, nuts and cooking oil |  |  |
|  | High-protein diet | - |  |  |  | 2046  (1023/1023) | 1. High intake of poultry red/animal/processed/organ meat, grains, fish, soups, leafy/ cruciferous vegetables and eggs |  |  |
|  | Sweets and Seafood pattern | Unhealthy |  |  |  | 2043  (1019/1024) | 1. Frequent intake of Cantonese desserts, molluscs, shellfish and SSBs |  |  |
| Hrolfsdottir, 2020[17] | Unhealthy diet score | Unhealthy | Iceland | white European | 75g OGTT  ≥1 of the following:  Fasting ≥ 5.1 mmol/L  1-hr ≥ 10 mmol/L  2-hr > 8.5 mmol/L | 709  (302/407) | 1. Low intake of fruit, vegetables fish, dairy, wholegrains, beans, nuts, seeds, and vitamin D supplementation 2. High intake of refined sugars and grains, and processed foods, dairy, and low-quality fat (i.e. using butter rather than oil) | FFQ – Diet over the previous month | During pregnancy:  11^th^ -14^th^ week |
| Hu, 2019[18] | Sweets and Seafood pattern | Unhealthy | China | Asian | 75g OGTT  ≥1 of the following:  Fasting ≥ 5.1 mmol/L  1-hr ≥ 10 mmol/L  2-hr > 8.5 mmol/L | 508  (255/253) | 1. Frequent intake of pastries, candid, sweet beverages, shrimps, crabs, fruit, mussels and red meat | FFQ – Diet over the previous 2 months | During pregnancy:  22^nd^ week |
|  | Fish- Seafood | - |  |  |  | 508  (255/253) | 1. Frequent intake of marine fish, shrimp, crabs and mussels, freshwater fish and seaweed 2. Infrequent intake of eggs, dairy products and rice |  |  |
|  | Traditional Asian diet | - |  |  |  | 508  (255/253) | 1. Frequent intake of tubers, vegetables, fruit, rice, red meat, eggs and nuts |  |  |
| Karamanos, 2014 | Mediterranean diet | Healthy^†^ | Mediterranean countries (Algeria, France, Greece, Italy, Lebanon, Malta, Morocco, Serbia, Syria, Tunisia) | Mediterranean | 75g OGTT  ≥2 of the following:  Fasting ≥ 5.3 mmol/L  1-hr ≥ 10 mmol/L  2-hr > 8.6 mmol/L  OR  ≥1 of the following:  Fasting ≥ 5.1 mmol/L  1-hr ≥ 10 mmol/L  2-hr > 8.5 mmol/L | 668  (334/334) | 1. High adherence to the Med Diet pyramid 2. Characterised by frequent intake of bread, cereals, legumes, vegetables, fruits, meat, fish, eggs, potatoes, cheese and dairy products 3. High ratio of olive oil to animal fat | FFQ | During pregnancy:  Before OGTT (24^th^-28 week) |
| Lamyian, 2017[19] | Fried/ fast food | Unhealthy | Iran | Tehranian | 75g OGTT  ≥2 of the following:  Fasting 95≥ mg/dL  1-hr ≥180mg /dL  2-hr ≥155mg /dL  3-hr ≥140mg /dL | 513  (256/257) | 1. High intake of hamburger, bologna, pizza, sausage and French fries | FFQ- Diet over the previous year | During pregnancy:  Before 6^th^ week |
| Le Donne, 2016[20] | Fish | - | Italy | white European | Not stated | 114  (104/10) | 1. Intake of tuna, swordfish, mackerel, salmon, anchovy, garfish, spatula, sardine, sea gilt-head bream, sea bass, cod, sea bream, perch and shellfish | FFQ- Diet over the previous week | During pregnancy:  34^th^ week |
| Liang, 2018[21] | High-protein diet | - | China | Asian | 75g OGTT  ≥1 of the following:  Fasting ≥ 5.1 mmol/L  1-hr ≥ 10 mmol/L  2-hr > 8.5 mmol/L | 714  (342/372) | 1. High intake of meats, fish, shrimps, dairy products, soybeans and nuts | FFQ- Diet over the previous day | Pre-pregnancy:  (Assessed at first routine ultrasound) |
|  | Fish | - |  |  |  | - | 1. Intake of fish and shrimp |  |  |
|  | Vegetable protein | - |  |  |  | 796  (439/357) | 1. Intake of Beans (soybeans and soybean products) and nuts |  |  |
|  | Meat pattern/  Animal protein | - |  |  |  | 5877  (2862/3015) | 1. High intake of animal protein (% TE) 2. Characterised by intake of meats, fish, shrimps and dairy products |  |  |
| Looman, 2018[22] | Healthy diet recommendations | Healthy | Australia | Australian Nationals | Self reported  Diagnostic critierea during study period  75g OGTT  Fasting ≥ 5.5mmol/L  2-hr ≥8.0 mmol/L  OR  75g OGTT  ≥1 of the following:  Fasting ≥ 5.1 mmol/L  1-hr ≥ 10 mmol/L  2-hr > 8.5 mmol/L | 2431  (1529/902) | 1. Low GI diet 2. Lowest group median GI 47.8, highest group median GI 56.7 | FFQ- Diet over the previous year | Pre- pregnancy: Dietary information collected in 2003 used as baseline. Pregnancies reported in years 2006, 2009, 2012 and 2015. |
|  | High protein diet | - |  |  |  | 2483  (1537/901) | 1. Low Carbohydrate Diet score |  |  |
|  | Carbohydrate | - |  |  |  | 3051  (1510/1541) | 1. Carbohydrate intake (%TE) |  |  |
| Mak, 2018[23] | Meat pattern | - | China | Asian | 75g OGTT  ≥1 of the following:  Fasting ≥ 5.1 mmol/L  1-hr ≥ 10 mmol/L  2-hr > 8.5 mmol/L | 892  446/446) | 1. Frequent intake of organ/processed meat, ox tripe, pig blood curd, squid, pork, and mushrooms | FFQ | During pregnancy: Between 15^th^-20^th^ week |
|  | Plant-based pattern | Healthy |  |  |  | 892  (446/446) | 1. High intakes of green leafy vegetable/ cruciferous/ gourd/melon family/red or orange/root/bean vegetables 2. High intake of potatoes, bean products, mushrooms and fruits 3. Low intake of lean pork meat |  |  |
|  | High-protein diet | - |  |  |  | 891  (445/446) | 1. High intake of eggs, milk, lean pork meat and fish 2. Low intake of bread and sea vegetables |  |  |
| Marí-Sanchis, 2018[24] | Meat pattern | - | Spain | Mediterranean | Self reported and then confirmed via an endocrinologist  Most common criterea used National Diabetes Group Criterea and Carpenter and Coustan cut-offs | 1649  (824/825) | 1. Frequent intake of red/processed/ unprocessed meats, poultry and rabbit | FFQ every 2 years | Pre- pregnancy |
| Mohanty, 2015[25] | Fish | - | USA | white European | 100g OGTT  ≥2 of the following:  Fasting ≥ 105 mg/dL  1-hr ≥190 mg/dL  2-hr ≥165 mg/dL  3-hr ≥ 145 mg/dL | 2418  (2116/302) | 1. Intake of shellfish, lean fish and fatty fish | FFQ – Diet 3 months prior to conception and during the first trimester. | During pregnancy:  16^th^ week |
| Osorio-Yáñez, 2017[26] | Fried/ fast food | Unhealthy | USA | white European | 100g OGTT  ≥2 of the following:  Fasting ≥ 95 mg/dL  1-hr ≥180 mg/dL  2-hr ≥155 mg/dL  3-hr ≥ 140 mg/dL | 4207  (3414/793) | 1. Intake of fried potatoes, fried chicken, fried fish, doughnuts and snack crisps | FFQ- Diet over the previous 3 months | During pregnancy:  15^th^ week |
| Pang, 2017[27] | High-protein diet | - | Singapore | Asian | 75g OGTT  ≥1 of the following:  Fasting ≥ 7 mmol/L  2-hr ≥ 7.8 mmol/L | 490  (245/245) | 1. Protein intake from animal and vegetable sources combined | FFQ- Diet over the previous 24 hours/ 3 days | During pregnancy:  26^th^-28^th^ week |
|  | Vegetable protein | - |  |  |  | 490  (245/245) | 1. Protein intake from vegetables, rice, noodles, desserts and beans |  |  |
|  | Meat pattern/ Animal protein | - |  |  |  | 490  (245/245) | 1. High intake of animal protein (% TE) |  |  |
| Schoenaker,  2015[28] | Mediterranean diet | Healthy | Australia | white European | Self reported  During study period following reccomendations used in Australia:  75g OGTT  Fasting ≥5.6 mmol/L  2-hr ≥ 8 mmol/L | 4376  (2249/2127) | 1. High intake of vegetables, legumes, nuts, tofu, rice, pasta, rye bread, wine and fish | FFQ- Diet over the previous 12 months | During pregnancy:  Varied times |
|  | Western diet | Unhealthy |  |  |  | 4365  (2137/2228) | 1. Frequent intake of red/ processed meat, snacks, sweets (including cakes, chocolate and biscuits), pizza and fruit juice |  |  |
|  | Plant-based pattern | Healthy |  |  |  | 4706  (2125/2554) | 1. Frequent intake of carrots, peas, cauliflower, broccoli, potatoes, pumpkin, green beans and cabbage |  |  |
| Tajima, 2017[29] | Fat | - | Japan | Asian | 75g OGTT  ≥1 of the following:  Fasting ≥ 5.1 mmol/L  1-hr ≥ 10 mmol/L  2-hr > 8.5 mmol | 125  (105/110) | 1. Total fat (%TE) | FFQ- Diet over the previous 3 days | During pregnancy:  First prenatal visit |
|  | Carbohydrate | - |  |  |  | 216  (108/108) | 1. Carbohydrate (%TE) |  |  |
| Tobias, 2012[30] | Healthy diet recommendations | Healthy | USA | white European | Self reported, most commonly by the National Diabetes Data Group Criterea | 9637  (6141/3496) | 1. High intake of AHEI diet 2. Increased intake of fruit, vegetables, cereal fibre, nuts and multivitamins 3. A high white: red meat ratio and PUFA:SFA ratio. 4. Moderate alcohol consumption 5. Decreased consumption of trans-fat | FFQ | Pre-pregnancy:  Most recent questionnaire (survey every two years) |
|  | Mediterranean diet |  |  |  |  | 8572  (5275/3297) | 1. Increased intake of fruits, vegetables, nuts, legumes, soy, fish and wholegrains 2. Moderate intake of alcohol and MUFA: SFA servings/d 3. Limited intake of red and processed meat |  |  |
| Tryggvadottir, 2016[31] | Healthy diet recommendations | Healthy | Iceland | white European | 75g OGTT  ≥1 of the following:  Fasting ≥ 5.1 mmol/L  1-hr ≥ 10 mmol/L  2-hr > 8.5 mmol/L | 168  (56/112) | 1. High adherence to the Healthy Eating Index. | FFQ – Diet over the previous 4 Days | During pregnancy:  20 weeks |
|  | Prudent diet | Healthy |  |  |  | 168  (56/112) | 1. Frequent intake of seafood, eggs, fruits, vegetables, vegetable oils, nuts, seeds, pasta, breakfast cereals, coffee, tea and cocoa powder 2. Limited intake of soft drinks and French fries. |  |  |
|  | Fish | - |  |  |  | NA | 1. Intake of fish, seafood and shellfish products |  |  |
| Yi, 2017[32] | Western diet | Unhealthy | China | Asian | 75g OGTT  ≥1 of the following:  Fasting ≥ 5.1 mmol/L  1-hr ≥ 10 mmol/L  2-hr > 8.5 mmol/L | 352  (173/179) | 1. Frequent intake of dairy, baked/fried food and white meat | 2 x FFQ- 24hr recall on 3 occasions | During pregnancy:  1 questionnaire during 5-15-week period, 1 questionnaire during  24-28-week period |
|  | Traditional Asian diet | - |  |  |  | 346  (179/167) | 1. Frequent intake of light-coloured vegetables, fine grain, red meat and tubers |  |  |
|  | Prudent diet | Healthy |  |  |  | 351  (181/170) | 1. Frequent intake of dark coloured vegetables and deep-sea fish |  |  |
| Yong, 2020[33] | Plant-based^†^ | Healthy | Malaysia | Asian | 75g OGTT  ≥1 of the following:  Fasting ≥ 5.6mmol/L  2-hr ≥ 7.8mmol/L | 300  (150/150) | 1. High intake of vegetables, nuts, seeds, legumes, fruits, eggs and dairy products | FFQ – Diet over the previous 6 months | During pregnancy:  ~10 weeks, ~12 weeks, ~27 weeks |
|  | Western diet^†^ | Unhealthy |  |  |  | 300  (150/150) | 1. High intake of poultry, meat, sweet foods, seafoods, oil, fat, rice, noodles and pasta |  |  |
| Zhang, 2006[34] | Prudent diet | Healthy | USA | white European | Self reported following a previous diagnosis. Most common criteria used was National Diabetes Group criterea | 5185  (2519/2666) | 1. High intake of fruit, green leafy vegetables, poultry and fish | FFQ- Diet over the previous year | Pre-pregnancy:  1-7 years |
|  | Western diet | Unhealthy |  |  |  | 5196  (2530/2666) | 1. Highest meat (poultry, red and processed), pizza, dessert, sweet, French fries, dairy products and refined grain intake |  |  |
| Zhang, 2014[35] | Healthy diet recommendations | Healthy | USA | white European | Self reported following a previous diagnosis. Most common criteria used was National Diabetes Group criterea | 8236  (4219/4017) | 1. Modified version of AHEI-2010 (10/11 components, excluding alcohol) 2. Higher intakes of vegetables, fruit, whole grains, nuts, long- chain (n-3) fatty acids, and PUFAs 3. Lower intakes of SSBs, red/processed meat, trans-fat, and sodium | FFQ- Diet over the previous year | Pre-pregnancy:  Most recent questionnaire |
| Zhou, 2018[16] | Plant-based pattern | Healthy | China | Asia | 75g OGTT  ≥1 of the following:  Fasting ≥ 5.1 mmol/L  1-hr ≥ 10 mmol/L  2-hr > 8.5 mmol/L | 131  (60/71) | 1. Frequent intake of root vegetables, melon, solanaceous/leafy/cruciferous vegetables, mushrooms, algae, beans and bean products | FFQ- Diet over the previous month | During pregnancy:  2 weeks before OGTT (OGTT at 24-28 weeks) |
|  | Meat pattern | - |  |  |  | 132  (79/53) | 1. Frequent intake of animal organs and blood, seafood and poultry |  |  |
|  | High-protein diet^††^ | - |  |  |  | 1356  (668/668) | 1. Total protein intake (%TE) |  |  |
|  | Animal protein^††^ |  |  |  |  | 1356  (668/668) | 1. Animal protein (%TE) |  |  |
|  | Fat^††^ | - |  |  |  | 1356  (668/668) | 1. Total fat (%TE) |  |  |
|  | Carbohydrate^††^ | - |  |  |  | 1356  (668/668) | 1. Carbohydrate (%TE) |  |  |
|  | Vegetable protein^††^ | - |  |  |  | 1356  (668/668) | 1. Vegetable protein (%TE) |  |  |

## Table C: DL vs HJSK results comparison.

Table showing the comparison between initial (DL) random effects model compared to the Hartung-Knapp-Sidik-Jonkman (HKSJ) model the main analyses. * represent studies including two relevant exposures. The random exclusion of one exposure from each of these studies was not found to impact the overall effect estimate for all exposures.

|  |  |  |  | Random effects | | | | | |
| --- | --- | --- | --- | --- | --- | --- | --- | --- | --- |
|  |  |  |  | D-L | | | HJSK | | |
| Exposure | Study type | Subclass | N | OR (95% CI) | P value | Tau² | OR (95% CI) | P value | Tau² |
| Healthy | RCT | Overall | 7* | 0.86 (0.69 - 1.08) | 0.19 | 0.01 | 0.88 (0.65-1.18) | 0.32 | 0.06 |
|  |  | Asian | 2 | 0.76 (0.48 - 1.20) | 0.24 | 0 | 0.72 (0.03 – 20.6) | 0.43 | 0.79 |
|  |  | Australian National | 1 | 0.91 (0.34 -2.47) | 0.85 |  | 0.91 (0.34 -2.47) | 0.85 |  |
|  |  | Mediterranean | 2 | 0.92 (0.43 - 1.96) | 0.83 | 0.22 | 0.92 (0.01-123.9) | 0.86 | 0.20 |
|  |  | White European | 2 | 0.69 (0.24 – 1.98) | 0.50 | 0.46 | 0.70 (0.01 - 630) | 0.63 | 0.41 |
|  | Observational | Overall | 22*** | 0.79 (0.70 - 0.89) | ≤0.001 | 0.05 | 0.78 (0.67 - 0.90) | ≤0.01 | 0.09 |
|  |  | Asian | 6* | 0.91 (0.42 - 1.53) | 0.28 | 0.01 | 0.91 (0.73 – 1.11) | 0.26 | 0.03 |
|  |  | Australian National | 1 | 0.92 (0.66 - 1.29) | 0.63 |  | 0.92 (0.66 - 1.29) | 0.63 |  |
|  |  | Mediterranean | 3 | 0.64 (0.35 - 1.15) | 0.14 | 0.20 | 0.64 (0.16 - 2.54) | 0.29 | 0.24 |
|  |  | White European | 12** | 0.76 (0.64 - 0.89) | ≤0.001 | 0.06 | 0.75 (0.61 - 0.93) | 0.01 | 0.12 |
|  | Combined | Overall | 29*** | 0.75 (0.66 -0.86) | ≤0.0001 | 0.05 | 0.80 (0.70 – 0.90) | 0.007 | 0.08 |
|  |  | Asian | 8* | 0.90 (0.78-1.04) | 0.18 | 0.01 | 0.87 (0.73 – 1.05) | 0.13 | 0.04 |
|  |  | Australian National | 2 | 0.92 (0.67 - 1.26) | 0.60 | 0.00 | 0.92 (0.88 – 0.97) | 0.03 | 0 |
|  |  | Mediterranean | 5 | 0.75 (0.50 - 1.07) | 0.11 | 0.12 | 0.74 (0.39 - 1.40) | 0.25 | 0.21 |
|  |  | White European | 14** | 0.77  (0.65 - 0.90) | ≤0.001 | 0.06 | 0.72 (0.58 - 0.90) | ≤0.01 | 0.11 |
| Healthy recommendations | RCT | Overall | 4 | 0.62  (0.29- 1.30) | 0.20 | 0.35 | 0.60 (0.17 - 2.15) | 0.29 | 0.46 |
|  |  | Asian | 1 | 0.16  (0.03 - 0.86) | 0.03 |  | 0.16 (0.03-0.86) | 0.03 |  |
|  |  | White European | 3 | 0.79 (0.42 – 1.50) | 0.47 | 0.18 | 0.78 (0.19 - 3.17) | 0.53 | 0.19 |
|  | Observational | Overall | 7 | 0.72 (0.60 - 0.86) | ≤0.001 | 0.04 | 0.72 (0.55- 0.94) | 0.02 | 0.09 |
|  |  | Australian National | 1 | 0.92 (0.66 - 1.29) | 0.63 |  | 0.92 (0.66 - 1.29) | 0.63 |  |
|  |  | White European | 6 | 0.69 (0.57 - 0.84) | ≤0.001 | 0.04 | 0.69 (0.50 - 0.94) | 0.03 | 0.10 |
|  | Combined | Overall | 11 | 0.73 (0.61 - 0.87) | ≤0.001 | 0.05 | 0.67 (0.52- 0.94) | 0.02 | 0.19 |
|  |  | Australian National | 1 | 0.92 (0.67 - 1.26) | 0.63 |  | 0.92 (0.67 - 1.26) | 0.63 |  |
|  |  | Asian | 1 | 0.16 (0.031-0.86) | 0.03 |  | 0.16 (0.031-0.86) | 0.03 |  |
|  |  | White European | 9 | 0.72 (0.60 - 0.87) | ≤0.001 | 0.04 | 0.71 (0.54 - 0.94) | 0.02 | 0.11 |
| Mediterranean | Observational | Overall | 5 | 0.66 (0.50 - 0.85) | ≤0.01 | 0.05 | 0.65 (0.41 - 1.036) | 0.06 | 0.11 |
|  |  | Mediterranean | 3 | 0.64 (0.35 - 1.15) | 0.14 | 0.20 | 0.64 (0.16 - 2.54) | 0.29 | 0.24 |
|  |  | White European | 2 | 0.66 (0.49 - 0.89) | ≤0.01 | 0.04 | 0.66 (0.10 - 4.55) | 0.22 | 0.03 |
|  | Combined | Overall | 6 | 0.66 (0.54 - 0.82) | ≤0.0001 | 0.0344 | 0.65 (0.46 - 0.92) | 0.03 | 0.08 |
|  |  | Mediterranean | 4 | 0.66 (0.45 - 0.95) | 0.03 | 0.0811 | 0.65 (0.32 - 1.31) | 0.15 | 0.14 |
|  |  | White European | 2 | 0.66 (0.49 - 0.89) | 0.01 | 0.0335 | 0.66 (0.10 - 4.55) | 0.22 | 0.66 |
| Prudent diet | Observational | Overall | 4 | 0.86 (0.59 - 1.26) | 0.39 | 0.0887 | 0.80 (0.36 - 1.77) | 0.44 | 0.18 |
|  |  | Asian | 2 | 0.81 (0.43 - 1.52) | 0.50 | 0.132 | 0.80 (0.01 - 51.50) | 0.63 | 0.81 |
|  |  | White European | 2 | 0.78 (0.29 - 2.10) | 0.61 | 0.441 | 0.77 (0.00 - 486.71) | 0.70 | 0.77 |
| Plant-based diet | Observational | Overall | 6 | 0.93 (0.82 - 1.06) | 0.30 | 0.0086 | 0.93 (0.79 - 1.10) | 0.32 | 0.01 |
|  |  | Asian | 3 | 0.91 (0.73 - 1.15) | 0.45 | 0.0253 | 0.92 (0.58 - 1.45) | 0.50 | 0.02 |
|  |  | White European | 3 | 0.94 (0.79 - 1.11) | 0.46 | 0.0028 | 0.95 (0.65 - 1.39) | 0.60 | 0.01 |
| Unhealthy diets | Observational | Overall | 13 | 1.44 (1.25 - 1.67) | ≤0.0001 | 0.0257 | 1.43 (1.20 - 1.72) | ≤0.001 | 0.06 |
|  |  | Asian | 4 | 1.04 (0.72 - 1.51) | 0.83 | 0.0701 | 1.04 (0.59 - 1.83) | 0.84 | 0.08 |
|  |  | Iranian | 1 | 2.12 (1.12 - 4.01) | 0.02 |  | 2.12 (1.12 - 4.01) | 0.02 |  |
|  |  | Mediterranean | 2 | 1.69 (1.21 - 2.35) | ≤0.01 | 0 | 1.68 (0.54 - 5.24) | 0.11 | 0.002 |
|  |  | White European | 6 | 1.59 (1.41 - 1.81) | ≤0.0001 | 0 | 1.57 (1.33 - 1.84) | ≤0.001 | 0.01 |
| Fried/ fast food | Observational | Overall | 4 | 1.66 (1.42 - 1.93) | ≤0.0001 | 0 | 1.65 (1.24 - 2.19) | ≤0.01 | 0.02 |
|  |  | Iranian | 1 | 2.12 (1.12 - 4.01) | 0.02 |  | 2.12 (1.12 - 4.01) | 0.02 |  |
|  |  | Mediterranean | 1 | 1.86 (1.13 - 3.06) | ≤0.01 |  | 1.86 (1.13 - 3.06) | ≤0.01 |  |
|  |  | White European | 2 | 1.57 (1.23 - 1.99) | 0.0004 | 0.01 | 1.55 (0.31 - 7.82) | 0.18 | 0.02 |
| Western diet | Observational | Overall | 7 | 1.51 (1.23 - 1.86) | ≤0.0001 | 0.0049 | 1.48 (1.09 - 2.01) | 0.02 | 0.05 |
|  |  | Asian | 2 | 1.09 (0.52 - 2.26) | 0.82 | 0.10 | 1.09 (0.01 - 129.82) | 0.86 | 0.13 |
|  |  | Mediterranean | 1 | 1.56 (1.00 - 2.43) | 0.05 |  | 1.55 (1.00 - 2.42) | 0.05 |  |
|  |  | White European | 3 | 1.60 (1.26 - 2.02) | ≤0.0001 | 0.0014 | 1.60 (0.90 - 2.82) | 0.07 | 0.03 |
| Sweets and Seafood pattern | Observational | Asian | 2 | 1.01 (0.58 - 1.74) | 0.98 | 0.13 | 1.01 (0.03 - 34.14) | 0.98 | 0.18 |
| Traditional Asian | Observational | Asian | 2 | 1.05 (0.15 - 7.30) | 0.96 | 1.81 | 1.05 (0 - 301,857) | 0.97 | 1.71 |
| Meat pattern | Observational | Overall | 7 | 1.41(1.22 - 1.63) | ≤0.0001 | 0.01 | 1.41 (1.14 - 1.75) | ≤0.01 | 0.03 |
|  |  | Asian | 4 | 1.34 (0.98 - 1.84) | 0.07 | 0.06 | 1.34 (0.79 - 2.23) | 0.18 | 0.07 |
|  |  | Mediterranean | 1 | 1.68 (1.07 -2.65) | 0.02 |  | 1.68 (1.07 - 2.65) | 0.02 |  |
|  |  | White European | 2 | 1.41(1.18 - 1.68) | ≤0.0001 | 0 | 1.40 (0.88 - 2.24) | 0.07 | 0.0003 |
| High protein diet | Observational | Overall | 7 | 1.19 (0.94 - 1.52) | 0.15 | 0.06 | 1.20 (0.88 - 1.65) | 0.20 | 0.08 |
|  |  | Asian | 5 | 1.13 (0.82 - 1.56) | 0.45 | 0.09 | 1.15 (0.68 - 1.93) | 0.50 | 0.36 |
|  |  | Australian National | 1 | 1.45(1.03 - 2.05) | 0.18 |  | 1.45 (1.03 - 2.04) | 0.18 |  |
|  |  | White European | 1 | 1.28 (0.9 - 1.84) | 0.17 |  | 1.28 (0.90 - 1.84) | 0.17 |  |
| Animal protein | Observational | Overall | 4 | 1.49 (1.25 -1.77) | < 0.0001 | 0 | 1.51 (1.16 – 1.97*)* | 0.02 | 0.01 |
|  |  | Asian | 3 | 1.51 (1.20 -1.89) | ≤0.001 | ≤0.01 | 1.53 (0.91 -2.57) | 0.07 | 0.02 |
|  |  | White European | 1 | 1.51 (1.03 - 2.20) | 0.03 |  | 1.51 (1.03 - 2.20) | 0.03 |  |
| Vegetable protein | Observational | Overall | 4 | 1.11 (0.72 - 1.70) | 0.67 | 0.12 | 1.10 (0.56 - 2.16) | 0.67 | 0.12 |
|  |  | Asian | 3 | 1.30 (0.95 - 1.77) | 0.10 | 0.0021 | 1.33 (0.67 - 2.63) | 0.22 | 0.03 |
|  |  | White European | 1 | 0.69 (0.49 -0.97) | 0.03 |  | 0.69 (0.97-90.49) | 0.03 |  |
| Fat | Observational | Overall | 3 | 1.50 (1.22 - 1.83) | ≤0.0001 | 0 | 1.53 (1.04 - 2.25) | 0.04 | 0.01 |
|  |  | Asian | 1 | 1.85  (1.21 - 2.83) | ≤0.01 |  | 1.86  (1.22 - 2.84) | ≤0.01 |  |
|  |  | White European | 2 | 1.41 (1.12 - 1.77) | ≤0.01 | 0 | 1.42 (0.73 - 2.76) | 0.20 | 0.04 |
| Carbohydrate | Observational | Overall | 3 | 0.49 (0.38 - 0.63) | ≤0.0001 | 0 | 0.49 (0.35 - 0.68) | 0.01 | 0.0026 |
|  |  | Asian | 2 | 0.44 (0.30 - 0.63) | ≤0.0001 | 0 | 0.44 (0.30 - 0.65) | 0.02 | <0.0001 |
|  |  | White European | 1 | 0.54 (0.38 -0.78) | ≤0.001 |  | 0.54 (0.38 - 0.77) | ≤0.001 |  |
| Fish | Observational | Overall | 5 | 0.87 (0.75 - 1.00) | 0.05 | 0 | 0.92 (0.73 - 1.15) | 0.35 | 0.02 |
|  |  | Asian | 2 | 1.09 (0.71 - 1.68) | 0.70 | 0 | 1.11 (0.06 - 19.30) | 0.72 | 0.04 |
|  |  | White European | 3 | 0.85 (0.75 -1.00) | 0.03 | 0 | 0.85 (0.77 - 0.94) | 0.02 | 0.0014 |

## Table D: Power analysis.

Detectable effect size based at an 80% power level for all exposures/ subgroups containing ≥ 2 studies.* Analysis represents power determined under a fixed effect model (tau^2^ of main DL analysis =0) . **A**: Analysis of RCTs. **B**. Observational studies. **C.** Combined Analysis. **D.** Sensitivity Analyses. **E.** Asian subgroups. **F.** Asian subgroups sensitivity analyses.

**A**

A

| Exposure | Subgroup | OR (95% CI) | Detectable ES (80% power) |
| --- | --- | --- | --- |
| Healthy | Overall | 0.86 (0.69 - 1.08) | 0.04 |
|  | Asian | 0.76 (0.48 - 1.20) | 0.17 |
|  | Mediterranean | 0.92 (0.43 – 1.96) | 0.09 |
|  | White European | 0.69 (0.24 – 1.98) | 0.18 |
| Healthy Recommendations | Overall | 0.77 (0.43 – 1.39) | 0.10 |
|  | White European | 0.79 (0.42 – 1.49) | 0.10 |

**B**

| Exposure | Subgroup | OR (95% CI) | Detectable ES (80% power) |
| --- | --- | --- | --- |
| Healthy | Overall | 0.79 (0.70 - 0.89) | <0.01 |
|  | Asian | 0.91 (0.78 - 1.07) | <0.01 |
|  | Mediterranean | 0.64 (0.35 - 1.15) | 0.03 |
|  | White European | 0.76 (0.64 - 0.90) | <0.01 |
| Healthy recommendations | Overall | 0.70 (0.56 - 0.86) | <0.01 |
|  | White European | 0.66 (0.53 - 0.83) | <0.01 |
| Mediterranean diet | Overall | 0.66 (0.50 - 0.85) | <0.01 |
|  | Mediterranean | 0.64 (0.35 - 1.15) | 0.03 |
|  | White European | 0.66 (0.49 - 0.89) | 0.02 |
| Prudent | Overall | 0.86 (0.59 - 1.26) | 0.03 |
|  | Asian | 0.81 (0.43 - 1.52) | 0.02 |
|  | White European | 0.78 (0.29 - 2.10) | 0.03 |
| Plant based | Overall | 0.93 (0.83 - 1.04) | <0.01 |
|  | Asian | 0.91 (0.75 - 1.11) | 0.02 |
|  | White European* | 0.92 (0.78 - 1.08) | 0.06 |
| Unhealthy | Overall | 1.44 (1.25 - 1.67) | <0.01 |
|  | Asian | 1.04 (0.72 - 1.51) | 0.09 |
|  | Mediterranean | 1.69 (1.21 - 2.35) | 0.07 |
|  | White European | 1.59 (1.41 - 1.81) | 0.04 |
| Fried/ fast food | Overall* | 1.66 (1.42 - 1.93) | 0.07 |
|  | White European | 1.57 (1.23 - 1.99) | <0.01 |
| Western | Overall | 1.51 (1.23 - 1.86) | <0.01 |
|  | Asian | 1.09 (0.52 - 2.26) | 0.03 |
|  | White European | 1.60 (1.26 - 2.02) | <0.01 |
| Sweet and Seafood | Asian | 1.01 (0.58 - 1.74) | 0.03 |
| Meat | Overall | 1.41 (1.22 - 1.63) | <0.01 |
|  | Asian | 1.34 (0.98 - 1.84) | 0.01 |
|  | White European | 1.41 ( 1.18 – 1.68) | 0.05 |
| High protein diet | Overall | 1.36 (1.05 - 1.76) | 0.02 |
|  | Asian | 1.42 (0.85 - 2.35) | 0.05 |
|  | White European* | 1.28 (1.06 - 1.55) | 0.05 |
| Asian Traditional | Asian | 1.05 (0.15 - 7.30) | 0.18 |
| Fish | Overall | 0.87 (0.75 - 1.00) | 0.11 |
|  | Asian | 1.09 (0.71 - 1.68) | 0.18 |
|  | White European | 0.85 (0.73 - 0.98) | 0.14 |
| Carbohydrate | Overall | 0.49 (0.38 - 0.63) | 0.11 |
|  | Asian | 0.44 (0.30 - 0.63) | 0.13 |
| Fat | Overall* | 1.50 (1.22 - 1.83) | 0.07 |
|  | White European* | 1.41 (1.12 - 1.77) | 0.08 |
| Animal protein | Overall* | 1.49 (1.25 - 1.77) | 0.05 |
|  | Asian* | 1.51 (1.20 - 1.77) | 0.06 |
| Vegetable protein | Overall | 1.11 (0.72 - 1.70) | 0.02 |
|  | Asian | 1.30 (0.95 - 1.77) | <0.01 |

**C**

| Exposure | Subgroup | OR (95% CI) | Detectable ES (80% power) |
| --- | --- | --- | --- |
| Healthy | Overall | 0.75 (0.66 -0.86) | <0.01 |
|  | Asian | 0.90 (0.78-1.04) | 0.02 |
|  | Mediterranean | 0.73 (0.50 - 1.07) | 0.03 |
|  | White European | 0.76 (0.64 - 0.90) | <0.01 |
| Healthy Recommendations | Overall | 0.74 (0.60 - 0.91) | <0.01 |
|  | White European | 0.70 (0.56 - 0.87) | <0.01 |
| Mediterranean | Overall | 0.66 (0.54 - 082) | <0.01 |
|  | Mediterranean | 0.66 (0.45 - 0.95) | 0.03 |
|  | White European | 0.66 (0.49 - 0.89) | 0.02 |

**D**

| Exposure | Analysis | Subclass | OR (95% CI) | Detectable ES (80% power) |
| --- | --- | --- | --- | --- |
| Healthy recommendations | Dietary assessment during pregnancy | Overall | 0.77 (0.63 – 0.93) | 0.09 |
|  |  | White European | 0.72 (0.58 – 0.89) | 0.11 |
|  | Obstetric adjustments | Overall | 0.67 (0.53 - 0.84) | 0.01 |
|  |  | White European | 0.62 (0.49 - 0.80) | 0.01 |
|  | BMI Healthy/ underweight | Overall | 0.71 (0.58 -0.87) | 0.01 |
|  |  | White European | 0.67 (0.54 - 0.84) | 0.01 |
|  | BMI overweight/ obese | Overall | 0.96 (0.62 – 1.47) | 0.10 |
|  |  | White European | 0.71 (0.26 – 1.93) | 0.15 |
|  | Older mothers | Overall | 0.73 (0.59 -0.90) | 0.01 |
|  |  | White European | 0.72 (0.58 – 0.89) | 0.02 |
| Healthy diets | Dietary assessment during pregnancy | Overall | 0.84 (0.71 – 1.00) | 0.01 |
|  |  | Asian | 0.93(0.75 – 1.10) | 0.02 |
|  |  | Mediterranean | 0.77 (0.47 - 1.28) | 0.05 |
|  |  | White European | 0.78 (0.50 – 1.22) | 0.01 |
|  | Obstetric adjustments | Overall | 0.76 (0.66 - 0.87) | 0.01 |
|  |  | Asian | 0.91 (0.75 - 1.11) | 0.01 |
|  |  | Mediterranean | 0.51 (0.27 -0.97) | 0.04 |
|  |  | White European | 0.73 (0.61 - 0.87) | 0.01 |
|  | BMI underweight/ healthy | Overall | 0.81 (0.71 - 0.93) | 0.01 |
|  |  | Asian | 0.89 (0.75 -1.07) | 0.02 |
|  |  | Mediterranean | 0.82 (0.62 - 1.08) | 0.02 |
|  |  | White European | 0.76 (0.61 - 0.95) | 0.01 |
|  | BMI overweight/ obese | Overall | 0.68 (0.49 -0.95) | 0.05 |
|  |  | Asian | 0.44 (0.09-2.03) | 0.47 |
|  |  | Mediterranean | 0.54 (0.17 -1.69) | 0.15 |
|  |  | White European | 0.66 (0.41 – 1.04) | 0.05 |
|  | Older mothers | Overall | 0.76 (0.68 – 0.84) | 0.01 |
|  |  | Asian* | 0.83 (0.69 – 1.00) | 0.10 |
|  |  | Mediterranean | 0.66 (0.43 – 1.00) | 0.04 |
|  |  | White European | 0.75 (0.66 – 0.86) | 0.01 |
| Mediterranean | Dietary assessment during pregnancy | Mediterranean | 0.51 (0.27 - 0.97) | 0.06 |
|  | Obstetric adjustments | Overall | 0.62 (0.48 - 0.79) | 0.01 |
|  |  | Mediterranean | 0.66 (0.49 - 0.89) | 0.06 |
|  |  | White European | 0.51 (0.27 - 0.97) | 0.02 |
|  | BMI underweight/ healthy | Overall | 0.73 (0.56 -0.96) | 0.01 |
|  |  | Mediterranean | 0.83 (0.63 -1.09) | 0.02 |
|  | BMI overweight/ obese | Overall | 0.48 (0.27-0.83) | 0.02 |
|  |  | Mediterranean | 0.22 (0.02 - 2.46) | NA |
|  |  | White European | 0.51 (0.37 - 0.70) | 0.02 |
|  | Older mothers | Overall | 0.65 (0.52 - 0.82) | 0.02 |
|  |  | Mediterranean | 0.58 (0.42 - 0.80) | 0.01 |
|  |  | White European | 0.75 (0.65 - 0.86) | 0.03 |
| Prudent diet | Dietary assessment during pregnancy | Overall | 0.66 (0.36 - 1.22) | 0.08 |
|  |  | Asian | 0.81 (0.42 - 1.52) | 0.09 |
|  | BMI underweight/ healthy | Overall | 1.04 (0.80 - 1.35) | 0.02 |
|  |  | Asian | 0.78 (0.37 - 1.65) | 0.05 |
|  | Older mothers | Overall | 0.80 (0.63 - 1.03) | 0.01 |
|  |  | Asian | 1.05 (0.72 - 1.53) | 0.21 |
|  |  | White European | 0.72 (0.55 - 0.92) | 0.01 |
| Unhealthy diets | Dietary assessment during pregnancy | Overall | 1.31 (1.03 - 1.67) | 0.02 |
|  |  | Asian | 1.01 (0.71 - 1.71) | 0.04 |
|  |  | White European | 1.52 (1.19 -1.94) | 0.01 |
|  | BMI underweight/ healthy | Overall | 1.73 (1.34 - 2.24) | 0.01 |
|  |  | Asian* | 1.29 (1.21 – 1.38) | 0.07 |
|  |  | Mediterranean* | 1.60 (1.16-2.21) | 0.10 |
|  |  | White European | 2.17 (1.56 -2.95) | 0.02 |
|  | BMI overweight/ obese | Overall | 1.32 (0.80 -1.21) | 0.03 |
|  |  | White European | 1.58 (0.96 -2.60) | 0.03 |
|  | Older mothers | Overall | 1.45 (1.24 – 1.70) | 0.01 |
|  |  | Asian | 1.28 (1.16 – 1.41) | 0.01 |
|  |  | White European | 1.68 (1.34 - 2.11) | 0.01 |
| Western diet | Dietary assessment during pregnancy | Overall* | 1.96 (0.13 - 3.06) | 0.21 |
|  | BMI underweight/ healthy | Overall* | 1.90 (1.55 -2.38) | 0.07 |
|  | BMI overweight/ obese | Overall | 1.32 (0.80 -1.21) | 0.03 |
|  |  | White European | 1.58 (0.96 -2.60) | 0.03 |
|  | Older mothers | Overall | 1.48 (1.23 - 1.78) | 0.01 |
|  |  | Asian | 1.19 (0.50 - 2.87) | 0.11 |
|  |  | White European | 1.55 (1.26 - 1.91) | 0.01 |
| Fried/ fast food | BMI underweight/ healthy | Overall | 2.03 (1.17 - 3.52) | 0.03 |
|  |  | White European | 2.13 (0.88 - 5.15) | 0.05 |
| Plant based pattern | Dietary assessment during pregnancy | Overall | 0.92 (0.72 - 1.17 | 0.02 |
|  |  | Asian | 0.90 (0.65 - 1.23) | 0.03 |
|  | Obstetric adjustments | Overall | 0.92 (0.81 - 1.04) | 0.01 |
|  |  | Asian | 0.91 (0.75 - 1.11) | 0.01 |
|  |  | White European | 0.92 (0.74 - 1.13) | 0.01 |
|  | BMI underweight/ healthy | Overall | 0.88 (0.76 - 1.03) | 0.01 |
|  |  | Asian | 0.88 (0.70 - 1.12) | 0.02 |
|  | BMI overweight/ obese | Overall* | 0.97 (0.65 -1.45) | 0.20 |
|  | Older mothers | Overall* | 0.88 (0.77 - 1.00) | 0.05 |
|  |  | Asian* | 0.79 (0.66 - 0.96) | 0.10 |
|  |  | White European* | 0.96 (0.80 - 1.16) | 0.01 |
| High protein diet | Dietary assessment during pregnancy | Asian | 1.69 (0.92 - 3.09) | 0.05 |
|  | Older mothers | Overall | 1.36 (1.01 - 1.83) | 0.06 |
|  |  | Asian | 1.45 (0.72 -2.90) | 0.06 |
|  |  | White European* | 1.28 (1.09 - 1.52) | 0.05 |
| Vegetable protein | Dietary assessment during pregnancy | Asian | 1.20 (0.84-1.73) | 0.03 |
|  | Older mothers | Overall | 0.79 (0.31 - 2.01) | 0.05 |

**E**

| Exposure | Subgroup | OR (95% CI) | Detectable ES (80% power) |
| --- | --- | --- | --- |
| Healthy | East Asian | 0.87 (0.72 – 1.06) | 0.02 |
|  | South/ South-east Asian | 0.81 (0.54 – 1.22) | 0.13 |
| Plant based | East Asian | 0.91 (0.73 – 1.15) | 0.18 |
| Unhealthy | East Asian | 1.10 (0.71 – 1.71) | 0.04 |
| Animal protein | East Asian | 1.81 (1.25 -2.62) | 0.06 |

| Exposure | Subgroup | Analysis | OR (95% CI) | Detectable ES (80% power) |
| --- | --- | --- | --- | --- |
| Healthy | East Asian | Dietary assessment during pregnancy | 0.94 (0.79 – 1.11) | 0.02 |
|  |  | Studies adjusted/ accounting for obstetric risk factors | 0.94 (0.79 – 1.11) | 0.02 |
|  |  | BMI under | 0.94 (0.78 - 1.13) | 0.02 |
|  |  | Older mothers | 0.84 (0.71 – 1.00) | 0.11 |
| Plant based | East Asian | Older mothers | 0.79 (0.65 - 0.96) | 0.09 |
| Unhealthy | East Asian | Older mothers | 1.29 (1.21 -1.38) | 0.11 |
| High Protein Diet | East Asian | Dietary assessment during pregnancy | 1.28 (0.73 - 2.63) | 0.01 |
| Vegetable protein | East Asian | Dietary assessment during pregnancy | 1.15 (0.80 – 1.64) | 0.06 |

**F**

## Table E: Stratification by Asian region.

Stratification of the Asian ethnic group by Asian region. The East Asian group was comprised of participants from China, Japan and South Korea**.** South/ South-East Asian region was comprised of women from India, Malaysia and Singapore. †Indicates an analysis which is the same as the initial analyses as all Asian studies were from a singular Asian subgroup. I^2^ value NA when N =1. **CI** = Confidence interval. **UI** = Uncertainty interval. **DL**, DerSimonian and Laird. **HKSJ**, Hartung-Knapp-Sidik-Jonkman. *represent studies including two relevant exposures. The random exclusion of one exposure from each of these studies was not found to impact the overall effect estimate for all exposures.

|  |  |  |  | DL | | HKSJ | | Heterogeneity |
| --- | --- | --- | --- | --- | --- | --- | --- | --- |
| Study type | Exposure | Asian region | N | OR (95% CI) | P value | OR (95% CI) | P value | I^2^ % (95% UI) |
| RCT | Healthy | South/South East Asia | 1 | 0.81 (0.50, 1.30) | 0.38 | 0.81 (0.50, 1.30) | 0.38 | NA |
| Observational | Healthy | East Asia | 5 | 0.91 (0.77, 1.09) | 0.32 | 0.90 (0.69 - 1.18) | 0.34 | 47 (0 -81) |
|  |  | South/South East Asia | 1 | 0.82 (0.38, 1.77) | 0.61 | 0.82 (0.38, 1.77) | 0.61 | NA |
|  | Plant based | East Asia | 3 | 0.91 (0.73, 1.15) | 1.10 | 0.92 (0.58 - 1.44) | 0.50 | 63 (63 -63) |
|  |  | South/South East Asia | 1 | 0.82 (0.38, 1.77) | 0.61 | 0.82 (0.38, 1.77) | 0.61 | NA |
|  | Prudent | East Asia ^†^ | 2 | 0.81 (0.43, 1.52) | 0.51 | 0.80 (0.01 - 51.50) | 0.63 | 53 (0 -88) |
|  | Unhealthy | East Asia | 3 | 1.10 (0.71, 1.71) | 0.67 | 1.10 (0.42 - 2.90) | 0.71 | 62 (62-62) |
|  |  | South/South East Asia | 1 | 0.79 (0.38, 1.64) | 0.53 | 0.79 (0.38, 1.64) | 0.53 | NA |
|  | Western diet | East Asia | 1 | 1.68 (0.66, 4.28) | 0.28 | 1.68 (0.66, 4.28) | 0.28 | NA |
|  |  | South/South East Asia | 1 | 0.79 (0.38, 1.64) | 0.53 | 0.79 (0.38, 1.64) | 0.53 | NA |
|  | Sweets and Seafood | East Asia ^†^ | 2 | 1.01 (0.58, 1.74) | 0.98 | 1.01 (0.03 - 34.14) | 0.98 | 79 (7 -950 |
|  | Meat pattern | East Asia | 3 | 1.31 (0.75, 2.30) | 0.34 | 1.31 (0.45 - 3.86) | 0.39 | 71 (1 -96) |
|  |  | South/South East Asia | 1 | 1.38 (1.10, 1.72) | 0.0053 | 1.38 (1.10, 1.72) | 0.01 | NA |
|  | High protein diet | East Asia | 4 | 1.28 (0.73, 2.26) | 0.39 | 1.28 (0.51 - 3.25) | 0.46 | 88 (71-95) |
|  |  | South/South East Asia | 1 | 2.15 (1.27, 3.64) | 0.0044 | 2.15 (1.27, 3.64) | 0.0044 | NA |
|  | Traditional-Asian | East Asia ^†^ | 2 | 1.05 (0.15, 7.30) | 0.96 | 1.05 (0 - 301,857) | 0.97 | 93 (75-98) |
|  | Fish | East Asia ^†^ | 2 | 1.09 (0.71, 1.68) | 0.96 | 1.11 (0.06 - 19.30) | 0.72 | 0 (0 - 0) |
|  | Fat | East Asia | 1 | 1.85 (1.21, 2.83) | 0.0020 | 1.85 (1.21, 2.83) | 0.002 | NA |
|  | Animal protein | East Asia | 2 | 1.81 (1.25, 2.62) | 0.0018 | 1.76 (0.16 - 1.95) | 0.20 | 0 (0-0) |
|  |  | South/South East Asia | 1 | 1.38 (1.10, 1.73) | 0.01 | 1.38 (1.10, 1.73) | 0.01 | NA |
|  | Vegetable protein | East Asia | 2 | 1.15 (0.80, 1.64) | 0.45 | 1.16 (0.22 - 6.20) | 0.47 | 0 (0-0) |
|  |  | South/South East Asia | 1 | 0.81 (0.5, 1.3) | 0.05 | 0.81 (0.5, 1.3) | 0.05 | NA |
|  | Carbohydrate | East Asia ^†^ | 2 | 0.91 (0.77, 1.09) | <0.0001 | 0.90 (0.69 - 1.18) | 0.34 | 0 |
| Combined | Healthy | East Asia | 5* | 0.91 (0.78, 1.07) | 0.28 | 0.90 (0.73 – 1.11) | 0.26 | 45 (0-74) |
|  |  | South/South East Asia | 1 | 0.91 (0.73, 1.15) | 0.61 | 0.91 (0.73, 1.15) | 0.61 | NA |

## Table F: Sensitivity analyses results

**A:** Healthy exposure **B:** Healthy recommendations **C:** Mediterranean diet. **D:** Prudent diet. **E:** Plant-based diet **F:** Unhealthy diets **G:** Fried/ fast food **H:** Western diet **I:** High-protein **J:** Vegetable protein**.** Mothers were classified into ‘BMI under’ or ‘BMI above’ if their BMI was below/above the relevant ethnic specific threshold for being overweight.

**A**

| Healthy diets | | | | | | | | | | | | | |
| --- | --- | --- | --- | --- | --- | --- | --- | --- | --- | --- | --- | --- | --- |
| Analysis | Included studies | N | Overall | | | White European | | Asian | | Mediterranean | | Australian National | |
|  |  |  | OR  (95% CI) | P value | I^2^ (%) | OR  (95% CI) | P value | OR  (95% CI) | P value | OR  (95% CI) | P value | OR  (95% CI) | P value |
| Dietary assessment during pregnancy | Assaf-Balut 2017, Assaf-Balut 2018, Flynn 2016, He 2015, Mak 2018, Markovic 2016, Opie 2016, Rono 2018, 2015, Simmons 2017, Tryggvadottir 2016, Zhou 2018, | 14*** | 0.84  (0.71 – 1.00) | 0.05 | 54  (16 – 75) | 0.78  (0.50 – 1.22) | 0.28 | 0.93  (0.75 – 1.10) | 0.39 | 0.68  (0.35 – 1.32) | 0.26 | 0.91  (0.34 – 2.47) | 0.86 |
| Studies adjusted/  accounting for obstetric risk factors | Assaf-Balut 2017, Assaf-Balut 2018, Bao 2014, Gicevic 2018, He 2015, Looman 2018, Mak 2018, Schoenaker 2015, Tobias 2012, Tryggvadottir 2016, Zhang 2006, Zhang 2014, Zhou 2006, Yong 2020 | 18*** | 0.77  (0.67-0.88) | ≤0.0001 | 78  (66 – 86) | 0.70  (0.58-0.85) | ≤0.001 | 0.94  (0.81 -1.08) | 0.38 | 0.51  (0.27-0.97) | 0.04 | 0.92  (0.66-1.28) | 0.64 |
| BMI under | Assaf-Balut 2017, Assaf-Balut 2018, Badon 2016, Bao 2014, Donazar-Ezcurra 2017, Gicevic 2018, He 2015,  Looman 2018, Mak 2018, Sahariah 2016, Tobias 2012, Zhang 2014, Zhou 2006 | 17* | 0.81  (0.71-0.93) | ≤0.01 | 75  (60 – 84) | 0.76  (0.61-0.95) | ≤0.01 | 0.89  (0.74-1.07) | 0.22 | 0.82  (0.62-1.08) | 0.15 | 0.92  (0.66-1.28) | 0.64 |
| BMI over | Assaf-Balut 2018, Flynn 2016, Karamanos 2014, Markovic 2016, Opie 2016, Rono 2018, Schoenaker 2015, Simmons 2017, Tryggvadottir 2016, Yong 2020 | 12** | 0.68  (0.49 -0.95) | 0.021 | 61  (27 – 79) | 0.66  (0.41 – 1.04) | 0.07 | 0.44  (0.09-2.03) | 0.49 | 0.54  (0.17-1.69) | 0.29 | 0.72  (0.36 -1.44) | 0.35 |
| Analysis of older mothers | Assaf-Balut 2017, Assaf-Balut 2018, Badon 2016, Bao 2014, Gicevic 2018, He 2015, Karamanos 2014  Markovic 2016, Opie 2016, Rono 2016  Simmons 2017, Tobias 2012, Tryggvadottir 2016, Yi 2017, Zhang 2006, Zhou 2006, Yong 2020 | 23*** | 0.76  (0.68 – 0.84) | <0.0001 | 50  (19 -69) | 0.75  (0.66 -0.86) | <0.0001 | 0.83  (0.69 - 1.00) | 0.06 | 0.66  (0.43-1.00) | 0.05 | 0.83  (0.70- 0.99) | 0.03 |

**B**

| Healthy recommendations | | | | | | | | | | | | | |
| --- | --- | --- | --- | --- | --- | --- | --- | --- | --- | --- | --- | --- | --- |
| Analysis | Included studies | N | Overall | | | White European | | Asian | | Australian National | | Mediterranean | |
|  |  |  | OR  (95% CI) | P value | I^2^ (%) | OR  (95% CI) | P value | OR  (95% CI) | P value | OR  (95% CI) | P value | OR  (95% CI) | P value |
| Dietary assessment during pregnancy | Markovic 2016, Opie 2016, Rono 2018, Simmons 2017, Tryggvadottir 2016 | 5 | 0.77  (0.63 – 0.93) | 0.01 | 62  (27 – 80) | 0.72  (0.58 – 0.89) | 0.003 | 0.38 (0.07 – 2.00) | 0.25 |  |  | 1.47  (0.69 - 3.16) | 0.31 |
| Studies adjusted/  accounting for obstetric risk factors | Gicevic 2018, Looman 2018, Tobias 2012, Tryggvadottir 2016, Zhang 2014 | 5 | 0.67  (0.53-0.84) | ≤0.001 | 74  (35 – 89) | 0.62  (0.49-0.80) | ≤0.001 |  |  | 0.92  (0.66- 1.29) | 0.63 |  |  |
| BMI under | Badon 2016, Gicevic 2018, Looman 2018, Tobias 2012, Zhang 2014, | 5 | 0.71  (0.58-0.87) | ≤0.001 | 73  (33 -89) | 0.67  (0.54-0.84) | ≤0.001 |  |  | 0.92  (0.66-1.29) | 0.63 |  |  |
| BMI over | Markovic 2016, Opie 2016, Rono 2018, Simmons 2017, Tryggvadottir 2016 | 5 | 0.96  (0.62 – 1.47) | 0.85 | 17  (0-62) | 0.71  (0.26 – 1.93) | 0.84 | 0.38 (0.07 – 2.00) | 0.25 | 0.91  (0.34-2.47) | 0.85 | 1.47  (0.69 - 3.16) | 0.31 |
| Analysis of older mothers | Badon 2016, Gicevic 2018, Markovic 2016, Opie 2016, Rono 2016, Tobias 2012, Tryggvadottir 2016, Zhang 2014 | 9 | 0.73  (0.59 -0.90) | 00.003 | 60  (23-80) | 0.72 (0.58 – 0.89) | 0.003 | 0.38 (0.07 – 2.00) | 0.25 | 0.91  (0.34-2.47) | 0.85 | 1.48  (0.69 -3.16) | 0.31 |

| Mediterranean diet | | | | | | | | | |
| --- | --- | --- | --- | --- | --- | --- | --- | --- | --- |
| Analysis | Included studies | N | Overall | | | White European | | Mediterranean | |
|  |  |  | OR  (95% CI) | P value | I^2^ (%) | OR  (95% CI) | P value | OR  (95% CI) | P value |
| Dietary assessment during pregnancy | Assaf-Balut 2017, Assaf-Balut 2018, | 2 | 0.51  (0.27 – 0.97) | 0.04 | 68  (0 -93) |  |  | 0.51  (0.27 – 0.97) | 0.04 |
| Studies adjusted/  accounting for obstetric risk factors | Assaf-Balut 2017, Assaf-Balut 2018, Schoenaker 2015, Tobias 2012, | 4 | 0.62  (0.48-0.79) | ≤0.0001 | 61  (0-87) | 0.66  (0.49-0.89) | 0.001 | 0.51  (0.27-0.97) | 0.04 |
| BMI under | Assaf-Balut 2017, Assaf-Balut 2018, Donazar-Ezcurra 2017, Tobias, 2012 | 4 | 0.73  (0.56-0.96) | ≤0.01 | 53  (0-84) | 0.59  (0.47-0.74) | ≤0.00001 | 0.83  (0.63-1.09) | 0.18 |
| BMI over | Assaf-Balut 2018, Karamos 2014, Schoenaker 2015 | 3 | 0.48  (0.27-0.83) | ≤0.01 | 64  (0-90) | 0.51  (0.37-0.7) | ≤0.0001 | 0.22  (0.02 – 2.46) | 0.22 |
| Analysis of older mothers | Assaf-Balut 2017, Assaf-Balut 2018, Karamanos 2014, Tobias 2012 | 4 | 0.65  (0.52- 0.82) | ≤0.001 | 46  (0-82) | 0.75  (0.65-0.86) | ≤0.0001 | 0.58  (0.42-0.8) | ≤0.001 |

**C**

| Prudent diet | | | | | | | | | |
| --- | --- | --- | --- | --- | --- | --- | --- | --- | --- |
| Analysis | Included studies | N | Overall | | | White European | | Asian | |
|  |  |  | OR  (95% CI) | P value | I^2^ (%) | OR  (95% CI) | P value | OR  (95% CI) | P value |
| Dietary assessment during pregnancy | He 2015, Tryggvadottir, 2016, Yi 2017 | 3 | 0.66  (0.36 – 1.22) | 0.18 | 65  (0 – 90) | 0.81  (0.013 – 49.83) | 0.63 | 0.44  (0.21 -0.92) | 0.03 |
| BMI under | He 2015, Yi 2017, Zhang 2006, | 3 | 1.04  (0.8-1.35) | ≤0.01 | 62  (0.52- 0.82) | 1.22  (0.99-1.50) | 0.07 | 0.78  (0.37-1.65) | 0.51 |
| BMI over | Tryggvadottir, 2016 | 1 |  |  |  | 0.31  (0.13-0.74) | ≤0.01 |  |  |
| Analysis of older mothers | He 2015, Tryggvadottir 2016, Yi 2017, Zhang 2006 | 4 | 0.80  (0.63-1.03) | 0.27 | 23  (0-88) | 0.72  (0.55-0.92) | ≤0.01 | 1.05  (0.72-1.53) | 0.80 |

| Plant-based diet | | | | | | | | | |
| --- | --- | --- | --- | --- | --- | --- | --- | --- | --- |
| Analysis | Included studies | N | Overall | | | White European | | Asian | |
|  |  |  | OR  (95% CI) | P value | I^2^ (%) | OR  (95% CI) | P value | OR  (95% CI) | P value |
| Dietary assessment during pregnancy | Flynn 2016, He 2015, Zhou 2018 | 3 | 0.92  (0.72 -1.17) | 0.51 | 64  (0 -90) | 0.90  (0.65 – 1.23) | 0.50 | 1.03  (0.64 –1.66) | 0.90 |
| BMI under | Bao 2014, He 2015, Mak 2018, Zhou 2006 | 4 | 0.88  (0.76-1.03) | 0.10 | 18  (0-87) | 0.91  (0.73-1.13) | 0.37 | 0.88  (0.7-1.12) | 0.31 |
| BMI over | Flynn 2016, Yong 2020 | 2 | 0.97  (0.65-1.45) | 0.87 | 0 | 1.03  (0.84-1.66) | 0.90 | 0.82  (0.38-1.77) | 0.61 |
| Analysis of older mothers | Bao 2014, Flynn 2016, He 2015, Yong 2020, Zhou 2006 | 5 | 0.88  (0.77-1.00) | 0.05 | 0  (0-63) | 0.96  (0.80 -1.16) | 0.70 | 0.79  (0.66-0.96) | 0.02 |

**E**

**D**

| Unhealthy diets | | | | | | | | | | | |
| --- | --- | --- | --- | --- | --- | --- | --- | --- | --- | --- | --- |
| Analysis | Included studies | N | Overall | | | White European | | Asian | | Mediterranean | |
|  |  |  | OR  (95% CI) | P value | I^2^ (%) | OR  (95% CI) | P value | OR  (95% CI) | P value | OR  (95% CI) | P value |
| Dietary assessment during pregnancy | Flynn 2016, Hrolfsdottir 2019, He 2015, Hu 2019, Osorio-Yáñez 2017, Yi 2017 | 6 | 1.31  (1.03 – 1.67) | 0.03 | 50  (0-80) | 1.52 (1.19 -1.94) | ≤0.001 | 1.01  (0.71 - 1.71) | 0.67 |  |  |
| BMI under | Bao 2014, Osorio-Yáñez 2017, Dominguez 2014, Donazar-Ezcurra 2017, Hrolfsdottir 2019, Yi 2017, Zhang 2006, He 2015, Hu | 9 | 1.73  (1.34 – 2.24) | ≤0.0001 | 82  (67 – 90) | 2.17 (1.56-2.95) | ≤0.0001 | 1.29  (1.21 – 1.38) | ≤0.0001 | 1.60  (1.16-2.21) | 0.01 |
| BMI over | Flynn, 2016 Schoenaker 2015, Yong 2020 | 3 | 1.32  (0.80-1.21) | 0.28 | 58  (0-88) | 1.58 (0.96 -2.60) | 0.07 | 0.79  (0.38-1.64) |  |  |  |
| Analysis of older mothers | Donazar-Ezcurra, Flynn 2016 2017, He 2015, Hrolfsdottir 2019, Hu 2019, Schoenaker 2015, Yi 2017, Yong 2020, Zhang 2006 | 9 | 1.45  (1.24 – 1.70) | ≤0.0001 | 51  (0 -77) | 1.68 (1.34 -2.11) | ≤0.0001 | 1.28  (1.16 – 1.41) | ≤0.0001 | 1.37  (0.89 -2.13) | 0.15 |

**F**

*The average age of first pregnancy in Iran was taken as the average age of first pregnancy in married women as this was the only available statistic. As marriage is common in Iran, this age should be representative of the age of first pregnancy in all mothers in the counts

**G**

| Fried/ fast food | | | | | | | | | | | |
| --- | --- | --- | --- | --- | --- | --- | --- | --- | --- | --- | --- |
| Analysis | Included studies | N | Overall | | | White European | | Iranian | | Mediterranean | |
|  |  |  | OR  (95% CI) | P value | I^2^ (%) | OR | P value | OR | P value | OR | P value |
| Dietary assessment during pregnancy | Osorio-Yáñez, 2017 | 1 |  |  |  | 1.27  (0.84 – 1.93) | 0.25 |  |  |  |  |
| BMI under | Bao, 2014, Dominguez, 2014, Osorio-Yáñez, 2017 | 3 | 2.03  (1.17-3.53) | 0.01 | 78 | 2.13  (0.88-5.15) | 0.09 |  |  | 1.80  (1.11-2.92) | 0.02 |

| Western diet | | | | | | | | | | | |
| --- | --- | --- | --- | --- | --- | --- | --- | --- | --- | --- | --- |
| Analysis | Included studies | N | Overall | | | White European | | Asian | | Mediterranean | |
|  |  |  | OR  (95% CI) | P value | I^2^ (%) | OR  (95% CI) | P value | OR  (95% CI) | P | OR  (95% CI) | P value |
| Dietary assessment during pregnancy | Flynn 2016, Yi 2017 | 2 | 1.96  (1.25 – 3.06) | 0.0033 | 0  (0-0) | 2.05  (1.23 -3.41) | 0.01 | 1.68  (0.66 – 4.28) | 0.28 |  |  |
| BMI under | Donazar-Ezcurra 2017, Yi 2017, Zhang 2006 | 3 | 1.90  (1.55-2.38) | <0.0001 | 0  (0-88.8) | 2.05  (1.62-2.61) | < 0.0001 | 1.94  (0.80 – 4.96) | 0.14 | 1.45  (0.94-2.25) | 0.10 |
| BMI over | Flynn 2016, Schoenaker 2015, Yong 2020 | 3 | 1.32  (0.80-1.21) | 0.28 | 58  (0-88) | 1.58  (0.96 -2.60) | 0.07 | 0.79  (0.38-1.64) | 0.53 |  |  |
| Analysis of older mothers | Donazar-Ezcurra 2017, Flynn 2016, Schoenaker 2015 , Yi 2017, Yong 2020, Zhang 2006, | 6 | 1.48  (1.23-1.78) | <0.0001 | 20  (0 - 64) | 1.55  (1.26-1.91) | <0.0001 | 1.19  (0.50-2.87) | 0.53 | 1.37  (0.89 – 2.13) | 0.10 |

**H**

| High protein diet | | | | | | | | | |
| --- | --- | --- | --- | --- | --- | --- | --- | --- | --- |
| Analysis | Included studies | N | Overall | | | White: Caucasian | | Asian | |
|  |  |  | OR  (95% CI) | P | I^2^ statistic  % | OR  (95% CI) | P | OR  (95% CI) | P |
| Dietary assessment during pregnancy | He 2015, Liang 2018, Pang 2017, Zhou 2018 | 4 | 1.69  (0.92 -3.09) | 0.09 | 88  (71 – 95) |  |  | 1.69  (0.92 -3.09) | 0.09 |
| Analysis of older mothers | Bao 2013, Bao 2014, Liang 2018, He 2015, Zhou 2018, | 5 | 1.35  (1.01 – 1.83) | 0.04 | 78  (46 – 91) | 1.28  (1.09 -1.52) | 0.003 | 1.45  (0.72 -2.90) | 0.30 |

| Vegetable protein | | | | | | | | | |
| --- | --- | --- | --- | --- | --- | --- | --- | --- | --- |
| Analysis | Included studies | N | Overall | | | White European | | Asian | |
|  |  |  | OR  (95% CI) | P | I^2^  % | OR  (95% CI) | P | OR  (95% CI) | P |
| Dietary assessment during pregnancy | Liang 2018, Pang 2017, Zhou 2018 | 3 | 1.20  (0.84- 1.73) | 0.32 | 24  (0-92) |  |  | 1.20  (0.84- 1.73) | 0.32 |
| Analysis of older mothers | Bao 2013, Liang 2018, Zhou 2018 | 3 | 0.94  (0.64-1.39) | 0.76 | 60  (0-89) | 0.68  (0.49 – 0.95) | 0.02 | 1.15  (0.81 – 1.61) | 0.43 |

**J**

**I**

## Table G: Sensitivity analyses results – HJSK analyses

Table showing the comparison between initial (DL) random effects model compared to the Hartung-Knapp-Sidik-Jonkman (HKSJ) model the sensitivity analyses. * represent studies including two relevant exposures. The random exclusion of one exposure from each of these studies was not found to impact the overall effect estimate for all exposures.

|  |  |  |  | Random effects | | | | | |
| --- | --- | --- | --- | --- | --- | --- | --- | --- | --- |
|  |  |  |  | DL | | | HJSK | | |
| Exposure | Study type | Subclass | N | OR (95% CI) | P value | Tau² | OR (95% CI) | P value | Tau² |
| Healthy diets | Dietary assessment during pregnancy | Overall | 14*** | 0.84(0.71 – 1.00) | 0.05 | 0.05 | 0.81 (0.63 - 1.03) | 0.08 | 0.14 |
|  |  | Asian | 5* | 0.93 (0.75 – 1.10) | 0.39 | 0.01 | 0.93 (0.78 – 1.10) | 0.39 | 0.14 |
|  |  | Mediterranean | 3 | 0.68 (0.35 - 1.32) | 0.26 | 0.25 | 0.69 (0.12 - 3.88) | 0.45 | 0.38 |
|  |  | White European | 5* | 0.78 (0.50 -1.22) | 0.28 | 0.11 | 0.74 (0.37 – 1.51) | 0.31 | 0.22 |
|  |  | Australian National | 1 | 0.91(0.33 – 2.48) | 0.86 |  | 0.91 (0.33 – 2.48) | 0.86 |  |
|  | Obstetric adjustments | Overall | 17** | 0.76 (0.66 - 0.87) | ≤0.0001 | 0.06 | 0.75 (0.63 - 0.89) | ≤0.01 | 0.10 |
|  |  | Asian | 4 | 0.91 (0.75 - 1.11) | 0.36 | 0.02 | 0.92 (0.70 -1.20) | 0.38 | 0.01 |
|  |  | Mediterranean | 2 | 0.51 (0.27 -0.97) | 0.04 | 0.15 | 0.52 (0.01 - 31.0) | 0.28 | 0.13 |
|  |  | Australian National | 1 | 0.91 (0.34 - 2.48) | 0.86 |  | 0.91 (0.34 - 2.48) | 0.86 |  |
|  |  | White European | 10** | 0.73 (0.61 - 0.87) | ≤0.001 | 0.06 | 0.72 (0.56 -0.92) | 0.02 | 0.12 |
|  | BMI under | Overall | 17* | 0.81 (0.71 - 0.93) | ≤0.01 | 0.05 | 0.81 (0.71 - 0.93) | ≤0.01 | 0.05 |
|  |  | Asian | 6 | 0.89 (0.75 -1.07) | 0.22 | 0.02 | 0.88 (0.68 - 1.14) | 0.26 | 0.05 |
|  |  | Mediterranean | 3 | 0.82 (0.62 - 1.08) | 0.15 | 0.02 | 0.82 (0.46 -1.48) | 0.29 | 0.03 |
|  |  | Australian National | 1 | 0.91 (0.34 - 2.48) | 0.86 |  | 0.91 (0.34 - 2.48) | 0.86 |  |
|  |  | White European | 7* | 0.76 (0.61 - 0.95) | ≤0.01 | 0.07 | 0.76 (0.57 -1.00) | 0.05 | 0.08 |
|  | BMI over | Overall | 12** | 0.68 (0.49 – 0.94) | 0.02 | 0.16 | 0.64 (0.40 -1.02) | 0.06 | 0.46 |
|  |  | Asian | 2 | 0.72 (0.36 – 1.44) | 0.34 | 0 | 0.69 (0.01 – 39.9) | 0.45 | 0.07 |
|  |  | Mediterranean | 3 | 0.54 (0.17 -1.69) | 0.29 | 0.73 | 0.44 (0.01 - 26.25) | 0.48 | 2.28 |
|  |  | Australian National | 1 | 0.91 (0.34 - 2.48) | 0.86 |  | 0.91 (0.34 - 2.48) | 0.86 |  |
|  |  | White European | 6* | 0.66 (0.41 – 1.04) | 0.07 | 0.19 | 0.64 (0.35 -1.20) | 0.13 | 0.25 |
|  | Older mothers | Overall | 23*** | 0.76 (0.68 -0.84) | <0.0001 | 0.02 | 0.76 (0.67 -0.87) | 0.0003 | 0.08 |
|  |  | Asian | 6 | 0.66 (0.43-1.00) | 0.03 | 0 | 0.83 (0.70 – 0.99) | 0.03 | 0 |
|  |  | Mediterranean | 4 | 0.66 (0.43 - 1.00) | 0.05 | 0.11 | 0.66 (0.28 - 1.59) | 0.23 | 0.24 |
|  |  | Australian National | 1 | 0.91 (0.34 - 2.48) | 0.86 |  | 0.91 (0.34 - 2.48) | 0.86 |  |
|  |  | White European | 12** | 0.75 (0.66 -0.86) | <0.0001 | 0.03 | 0.76 (0.63 -0.90) | 0.005 | 0.07 |
| Healthy recommendations | Dietary assessment during pregnancy | Overall | 7** | 0.77 (0.63-0.93) | 0.01 | 0.05 | 0.78 (0.60 – 1.00) | 0.049 | 0.11 |
|  |  | Asian | 1 | 0.38 (0.07 – 2.00) | 0.25 |  | 0.38 (0.07 – 2.00) | 0.25 |  |
|  |  | Mediterranean | 1 | 1.48 (0.69 - 3.16) | 0.31 |  | 1.48 (0.69 - 3.16) | 0.31 |  |
|  |  | White European | 5* | 0.72 (0.58 – 0.89) | 0.03 | 0.04 | 0.73 (0.55 -0.96) | 0.03 | 0.09 |
|  | Obstetric adjustments | Overall | 5 | 0.67 (0.53 - 0.84) | ≤0.001 | 0.04 | 0.66 (0.44 - 1.00) | 0.05 | 0.11 |
|  |  | Australian National | 1 | 0.92 (0.66 - 1.29) | 0.64 |  | 0.92 (0.66 - 1.29) | 0.64 |  |
|  |  | White European | 4 | 0.62 (0.49 - 0.80) | ≤0.001 | 0.04 | 0.65 (0.37 - 0.98) | 0.05 | 0.10 |
|  | BMI Healthy/ underweight | Overall | 5 | 0.71 (0.58 -0.87) | ≤0.001 | 0.04 | 0.70 (0.50 - 0.98) | 0.03 | 0.11 |
|  |  | Australian National | 1 | 0.92 (0.66 - 1.29) | 0.64 |  | 0.92 (0.66 - 1.29) | 0.64 |  |
|  |  | White European | 4 | 0.67 (0.54 - 0.84) | ≤0.001 | 0.04 | 0.67 (0.47 - 0.97) | 0.04 | 0.04 |
|  | BMI over | Overall | 6* | 0.96 (0.65-1.42) | 0.85 | 0.04 | 0.86 (0.46 – 1.63) | 0.58 | 0.23 |
|  |  | Asian | 1 | 0.38 (0.07 - 2.00) | 0.26 |  | 0.38 (0.07 - 2.00) | 0.26 |  |
|  |  | Mediterranean | 1 | 1.48 (0.69 - 3.16) | 0.31 |  | 1.48 (0.69 - 3.16) | 0.31 |  |
|  |  | White European | 4 | 0.90 (0.56 – 1.45) | 0.66 | 0.06 | 0.81 (0.32 – 2.06) | 0.53 | 0.22 |
|  | Older mothers | Overall | 10* | 0.73 (0.59 -0.90) | 0.003 | 0.05 | 0.73 (0.55 -0.97) | 0.03 | 0.13 |
|  |  | Asian | 1 | 0.38 (0.07 - 2.00) | 0.26 |  | 0.38 (0.07 - 2.00) | 0.26 |  |
|  |  | Australian National | 1 | 0.91 (0.34 -2.48) | 0.86 |  | 0.91 (0.34 -2.48) | 0.86 |  |
|  |  | Mediterranean | 1 | 1.48 (0.69 - 3.16) | 0.31 |  | 1.48 (0.69 - 3.16) | 0.31 |  |
|  |  | White European | 7 | 0.72 (0.58 – 0.89) | 0.003 | 0.04 | 0.73 (0.55 - 0.96) | 0.03 | 0.09 |
| Mediterranean diet | Dietary assessment during pregnancy | Mediterranean | 2 | 0.51 (0.27 - 0.97) | 0.04 | 0.15 | 0.517 (0.009 - 31.48) | 0.29 | 0.13 |
|  | Obstetric adjustments | Overall | 4 | 0.62 (0.48 - 0.79) | ≤0.0001 | 0.04 | 0.60 (0.38 - 0.95) | 0.04 | 0.06 |
|  |  | Mediterranean | 2 | 0.66 (0.49 - 0.89) | ≤0.01 | 0.03 | 0.52 (0.01 - 31.22) | 0.29 | 0.36 |
|  |  | White European | 2 | 0.51 (0.27 - 0.97) | 0.04 | 0.15 | 0.66 (0.10 - 4.55) | 0.22 | 0.17 |
|  | BMI under | Overall | 4 | 0.73 (0.56 -0.96) | ≤0.01 | 0.03 | 0.74 (0.49 - 1.12) | 0.10 | 0.04 |
|  |  | Mediterranean | 3 | 0.83 (0.63 -1.09) | 0.18 | 0.01 | 0.82 (0.46 - 1.48) | 0.29 | 0.03 |
|  |  | White European | 1 | 0.59 (0.47 -0.47) | ≤ 0.0001 |  | 0.59 (0.47 -0.47) | ≤0.0001 |  |
|  | BMI over | Overall | 3 | 0.48 (0.27-0.83) | ≤0.01 | 0.13 | 0.33 (0.016 - 6.89) | 0.26 | 1.25 |
|  |  | Mediterranean | 2 | 0.22 (0.02 - 2.46) | 0.22 | 2.59 | 0.22 (0 - >1000) | 0.44 | 2.32 |
|  |  | White European | 1 | 0.51 (0.37 - 0.70) | ≤ 0.0001 |  | 0.51 (0.37 - 0.70) | ≤0.0001 |  |
|  | Older mothers | Overall | 4 | 0.65 (0.52 - 0.82) | ≤ 0.001 | 0.02 | 0.62 (0.40 - 0.99) | 0.05 | 0.06 |
|  |  | Mediterranean | 3 | 0.58 (0.42 - 0.80) | ≤ 0.001 | 0.03 | 0.56 (0.25 - 1.25) | 0.09 | 0.07 |
|  |  | White European | 1 | 0.75 (0.65 - 0.86) | ≤ 0.0001 |  | 0.75 (0.65 - 0.86) | ≤ 0.0001 |  |
| Prudent diet | Dietary assessment during pregnancy | Overall | 3 | 0.66 (0.36 - 1.22) | 0.19 | 0.19 | 0.68 (0.21 - 2.21) | 0.30 | 0.13 |
|  |  | Asian | 2 | 0.81 (0.42 - 1.52) | 0.51 | 0.13 | 0.81 (0.13 - 49.8) | 0.63 | 0.13 |
|  |  | White European | 1 | 0.44 (0.21 - 0.92) | 0.03 |  | 0.44 (0.21 - 0.92) | 0.03 |  |
|  | BMI under | Overall | 3 | 1.04 (0.80 - 1.35) | 0.77 | 0.03 | 0.95 (0.32 - 2.80) | 0.85 | 0.16 |
|  |  | Asian | 2 | 0.78 (0.37 - 1.65) | 0.51 | 0.22 | 0.78 (0.01 - 98.47) | 0.64 | 0.20 |
|  |  | White European | 1 | 1.22 (0.99 -1.50) | 0.07 |  | 1.22 (0.99 -1.50) | 0.07 |  |
|  | BMI over | White European | 1 | 0.31 (0.13 -0.74) | ≤ 0.001 |  | 0.31 (0.13 -0.74) | ≤ 0.001 |  |
|  | Older mothers | Overall | 4 | 0.80 (0.63 - 1.03) | 0.08 | 0.02 | 0.81 (0.52- 1.26) | 0.22 | 0.05 |
|  |  | Asian | 2 | 1.05 (0.72 - 1.53) | 0.80 | 0 | 1.05 (1.02 - 1.09) | 0.03 | ≤ 0.0001 |
|  |  | White European | 2 | 0.72 (0.55 - 0.92) | ≤0.01 | 0.01 | 0.70 (0.11 - 4.48) | 0.25 | 0.02 |
| Plant based pattern | Dietary assessment during pregnancy | Overall | 3 | 0.92 (0.72 - 1.17) | 0.51 | 0.03 | 0.92 (0.58 - 1.48) | 0.54 | 0.02 |
|  |  | Asian | 2 | 0.90 (0.65 - 1.23) | 0.50 | 0.04 | 0.90 (0.11 - 7.03) | 0.62 | 0.04 |
|  |  | White European | 1 | 1.03 (0.64 - 1.66) | 0.90 |  | 1.03 (0.64 - 1.66) | 0.90 |  |
|  | BMI under | Overall | 4 | 0.88 (0.76 - 1.03) | 0.10 | ≤ 0.01 | 0.89 (0.68 -1.15) | 0.25 | 0.02 |
|  |  | Asian | 3 | 0.88 (0.70 - 1.12) | 0.31 | 0.02 | 0.89 (0.52 - 1.51) | 0.44 | 0.03 |
|  |  | White European | 1 | 0.91 (0.73 -1.13) | 0.37 |  | 0.91 (0.73 -1.13) | 0.37 |  |
|  | BMI over | Overall | 2 | 0.97 (0.65 -1.45) | 0.87 | 0 | 0.97 (0.26 -3.56) | 0.79 | ≤ 0.01 |
|  |  | Asian | 1 | 1.03 (0.64 -1.66) | 0.90 |  | 1.03 (0.64 -1.66) | 0.90 |  |
|  |  | White European | 1 | 0.82 (0.38 -1.77) | 0.61 |  | 0.82 (0.38 -1.77) | 0.61 |  |
|  | Older mothers | Overall | 5 | 0.88 (0.77 - 1.00) | 0.05 | 0 | 0.87 (0.76 - 1.00) | 0.06 | ≤ 0.01 |
|  |  | Asian | 3 | 0.79 (0.66 - 0.96) | 0.02 | 0 | 0.79 (0.71 - 0.89) | ≤0.01 | 0 |
|  |  | White European | 2 | 0.96 (0.80 - 1.16) | 0.70 | 0 | 0.98 (0.42 - 2.30) | 0.42 | 0 |
| Unhealthy diets | Dietary assessment during pregnancy | White European | 2 | 0.96 (0.80 - 1.16) | 0.70 | 0 | 0.98 (0.42 - 2.30) | 0.42 | 0 |
|  |  | Asian | 3 | 1.01 (0.71 - 1.71) | 0.67 | 0.09 | 1.10 (0.42 - 2.90) | 0.71 | 0.10 |
|  |  | White European | 3 | 1.52 (1.19 -1.94) | ≤0.001 | ≤0.0001 | 1.54 (0.88 -2.68) | 0.08 | 0.02 |
|  | BMI under | Overall | 9 | 1.73 (1.34 - 2.24) | ≤0.0001 | 0.12 | 1.73 (1.32 - 2.27) | ≤0.01 | 0.09 |
|  |  | Asian | 3 | 1.29 (1.21 - 1.38) | ≤0.0001 | 0 | 1.29 (0.81 - 2.04) | 0.15 | 0.03 |
|  |  | Mediterranean | 2 | 1.60 (1.16 - 2.21) | ≤0.01 | 0 | 1.60 (0.41 - 6.29) | 0.14 | ≤0.01 |
|  |  | White European | 4 | 2.17 (1.59 - 2.96) | ≤0.0001 | 0.07 | 2.16 (1.23 - 3.79) | 0.02 | 0.09 |
|  | BMI over | Overall | 3 | 1.32 (0.80 -1.21) | 0.28 | 0.11 | 1.31 (0.42 - 4.13) | 0.41 | 0.14 |
|  |  | Asian | 1 | 0.79 (0.38 - 1.64) | 0.53 |  | 0.79 (0.38 - 1.64) | 0.53 |  |
|  |  | White European | 2 | 1.58 (0.96 -2.60) | 0.07 | 0.07 | 1.58 (0.062 - 40.16) | 0.34 | 0.07 |
|  | Older mothers | Overall | 9 | 1.45 (1.24 - 1.70) | ≤0.0001 | 0.02 | 1.46 (1.17 - 1.82) | ≤0.01 | 0.06 |
|  |  | Asian | 4 | 1.28 (1.16 - 1.41) | ≤0.0001 | ≤0.01 | 1.20 (0.77 - 1.87) | 0.28 | 0.06 |
|  |  | Mediterranean | 1 | 1.37 (0.89 - 2.13) | 0.15 |  | 1.37 (0.89 - 2.13) | 0.15 |  |
|  |  | White European | 4 | 1.68 (1.34 - 2.11) | ≤0.0001 | 0.02 | 1.69 (1.16 - 2.48) | 0.02 | 0.03 |
| Fried/ fast food | Dietary assessment during pregnancy | White European | 1 | 1.28 (0.84 - 1.93) | 0.25 |  | 1.28 (0.84 - 1.93) | 0.25 |  |
|  | BMI under | Overall | 3 | 2.03 (1.17 - 3.52) | 0.01 | 0.19 | 2.03 (0.64 -6.48) | 0.12 | 0.16 |
|  |  | Mediterranean | 1 | 1.80 (1.11 - 2.91) | 0.02 |  | 1.80 (1.11 - 2.91) | 0.02 |  |
|  |  | White European | 2 | 2.13 (0.88 - 5.15) | 0.10 | 0.36 | 2.13 (0.01 - 657.06) | 0.34 | 0.33 |
| Western diet | Dietary assessment during pregnancy | Overall | 2 | 1.96 (0.13 - 3.06) | ≤0.01 | 0 | 1.96 (0.68 - 5.66) | 0.08 | 0.04 |
|  |  | Asian | 1 | 1.68 (0.66 - 4.27) | 0.28 |  | 1.68 (0.66 - 4.27) | 0.28 |  |
|  |  | White European | 1 | 2.05 (1.23 - 3.41) | ≤0.01 |  | 2.05 (1.23 - 3.41) | ≤0.01 |  |
|  | BMI under | Overall | 3 | 1.90 (1.55 -2.38) | ≤0.0001 | 0 | 1.85 (1.16- 2.97) | 0.03 | 0.01 |
|  |  | Asian | 1 | 1.94 (0.80 - 4.96) | 0.14 |  | 1.94 (0.80 - 4.96) | 0.14 |  |
|  |  | Mediterranean | 1 | 1.45 (0.94 - 2.25) | 0.10 |  | 1.45 (0.94 - 2.25) | 0.10 |  |
|  |  | White European | 1 | 2.05 (1.62 - 2.61) | ≤0.001 |  | 2.05 (1.62 - 2.61) | ≤0.001 |  |
|  | BMI over | Overall | 3 | 1.32 (0.80 -1.21) | 0.28 | 0.11 | 1.31 (0.42 - 4.13) | 0.41 | 0.14 |
|  |  | Asian | 1 | 0.79 (0.38 - 1.64) | 0.53 |  | 0.79 (0.38 - 1.64) | 0.53 |  |
|  |  | White European | 2 | 1.58 (0.96 -2.60) | 0.07 | 0.07 | 1.58 (0.062 - 40.16) | 0.33 | 0.07 |
|  | Older mothers | Overall | 6 | 1.48 (1.23 - 1.78) | ≤0.0001 | 0.01 | 1.47 (1.09 - 1.96) | 0.02 | 0.06 |
|  |  | Asian | 2 | 1.19 (0.50 - 2.87) | 0.53 | 0.23 | 1.19 (0.004 - 352.62) | 0.76 | 0.22 |
|  |  | Mediterranean | 1 | 1.37 (0.89 - 2.13) | 0.10 |  | 1.37 (0.89 - 2.13) | 0.10 |  |
|  |  | White European | 3 | 1.55 (1.26 - 1.91) | ≤0.0001 | ≤0.01 | 1.56 (0.95 - 2.55) | 0.06 | 0.02 |
| High protein diet | Dietary assessment during pregnancy | Asian | 4 | 1.69 (0.92 - 3.09) | 0.09 | 0.32 | 1.20 (0.77 - 1.87) | 0.28 | 0.06 |
|  | Older mothers | Overall | 5 | 1.36 (1.01 - 1.83) | 0.04 | 0.08 | 1.36 (0.85 - 2.19) | 0.14 | 0.11 |
|  |  | Asian | 3 | 1.45 (0.72 -2.90) | 0.30 | 0.32 | 1.45 (0.37 - 5.64) | 0.36 | 0.24 |
|  |  | White European | 2 | 1.28 (1.09 - 1.52) | ≤0.01 | 0 | 1.28 (1.28 - 1.29) | ≤0.001 | ≤0.0001 |
| Vegetable protein | Dietary assessment during pregnancy | Asian | 3 | 1.20 (0.84-1.73) | 0.32 | 0.03 | 1.21 (0.53- 2.76) | 0.43 | 0.06 |
|  | Older mothers | Overall | 3 | 0.94 (0.64 - 1.40) | 0.76 | 0.07 | 0.94 (0.41 - 2.18) | 0.78 | 0.07 |
|  |  | Asian | 2 | 1.15 (0.82 - 1.610 | 0.43 | 0 | 1.15 (0.31 - 4.20) | 0.40 | ≤0.01 |
|  |  | White European | 1 | 0.68 (0.49 - 0.95) | 0.02 |  | 0.68 (0.49 - 0.95) | 0.02 |  |

## Table H: Sensitivity analyses of Asian subgroups – HJSK analyses

Table showing the comparison between initial (DL) random effects model compared to the Hartung-Knapp-Sidik-Jonkman (HKSJ) model the sensitivity analyses of Asian subgroups. * represent studies including two relevant exposures. The random exclusion of one exposure from each of these studies was not found to impact the overall effect estimate for all exposures.

|  |  |  |  | Random effects | | | | | |
| --- | --- | --- | --- | --- | --- | --- | --- | --- | --- |
|  |  |  |  | D-L | | | HJSK | | |
| Exposure | Analysis | Subclass | N | OR (95% CI) | P value | Tau² | OR (95% CI) | P value | Tau² |
| Healthy | Dietary assessment during pregnancy | East Asian | 4* | 0.94 (0.79 - 1.11) | 0.45 | 0.01 | 0.94 (0.73 -1.20) | 0.47 | ≤0.01 |
|  | Studies adjusted/ accounting for obstetric risk factors | East Asian | 4* | 0.94 (0.79 - 1.11) | 0.45 | 0.01 | 0.94 (0.73 - 1.20) | 0.47 | 0.012 |
|  |  | South/ South-east Asian | 1 | 0.82 (0.38 - 1.77) | 0.61 |  | 0.82 (0.38 - 1.77) | 0.61 |  |
|  | BMI under | East Asian | 4 | 0.94 (0.78 - 1.13) | 0.48 | 0.02 | 0.94 (0.70 - 1.26) | 0.52 | 0.02 |
|  |  | South/ South-east Asian | 1 | 0.81 (0.50 - 1.30) | 0.38 |  | 0.81 (0.50 - 1.30) | 0.38 |  |
|  | Older mothers | East Asian | 3* | 0.84 (0.69 - 1.02) | 0.08 | 0 | 0.86 (0.55 - 1.34) | 0.29 | 0.01 |
|  | BMI over | South/ South-east Asian | 1 | 0.82 (0.38 - 1.77) | 0.61 |  | 0.82 (0.38 - 1.77) | 0.61 |  |
| Plant based | Dietary assessment during pregnancy | East Asian ^†^ | 2 | 0.90 (0.65 - 1.23) | 0.50 | 0.04 | 0.90 (0.11 - 7.03) | 0.62 | 0.04 |
|  | Studies adjusted/ accounting for obstetric risk factors | East Asian | 3 | 1.02 (0.90 - 1.17) | 0.72 | 0 | 1.02 (0.89 - 1.19) | 0.61 | ≤0.01 |
|  |  | South/ South-east Asian | 1 | 0.82 (0.38 - 1.77) | 0.61 |  | 0.82 (0.38 - 1.77) | 0.61 |  |
|  | BMI under | East Asian ^†^ | 3 | 0.88 (0.70 - 1.12) | 0.31 | 0.02 | 0.90 (0.69-1.18) | 0.34 | 0.04 |
|  | BMI over | South/ South-east Asian | 1 | 0.82 (0.38 - 1.77) | 0.61 |  | 0.82 (0.38 - 1.77) | 0.61 |  |
|  | Older mothers | East Asian | 2 | 0.84 (0.71 – 1.00) | 0.05 | 0 | 0.85 (0.68 – 1.07) | 0.11 | 0.01 |
|  |  | South/ South-east Asian | 1 | 0.82 (0.38 - 1.77) | 0.61 |  | 0.82 (0.38 - 1.77) | 0.61 |  |
| Prudent | BMI under | East Asian ^†^ | 2 | 0.78 (0.37 - 1.65) | 0.51 | 0.22 | 0.78 (0.01 - 98.47) | 0.64 | 0.19 |
|  | Older mothers | East Asian ^†^ | 2 | 1.05 (0.72 - 1.53) | 0.80 | 0 | 1.05 (1.02 - 1.09) | 0.03 | ≤0.0001 |
| Unhealthy | Dietary assessment during pregnancy | East Asian ^†^ | 3 | 1.01 (0.71 - 1.71) | 0.67 | 0.09 | 1.10 (0.42 - 2.90) | 0.71 | 0.10 |
|  | BMI under | East Asian ^†^ | 3 | 1.29 (1.21 - 1.38) | ≤0.0001 | 0 | 1.29 (0.81 - 2.04) | 0.15 | 0.03 |
|  | BMI over | South/ South-east Asian ^†^ | 1 | 0.79 (0.38 - 1.64) | 0.53 |  | 0.79 (0.38 - 1.64) | 0.53 |  |
|  | Older mothers | East Asian | 3 | 1.29 (1.21 -1.38) | ≤0.0001 | 0 | 1.29 (0.81 - 2.04) | 0.15 | 0.03 |
|  |  | South/ South-east Asian | 1 | 0.79 (0.38 - 1.64) | 0.53 |  | 0.79 (0.38 - 1.64) | 0.53 |  |
| High protein diet | Dietary assessment during pregnancy | East Asian | 4 | 1.28 (0.73 - 2.63) | 0.39 | 0.2813 | 1.28 (0.51 - 3.25) | 0.46 | 0.28 |
|  |  | South/ South-east Asian | 1 | 0.79 (0.38 - 1.64) | 0.53 |  | 0.79 (0.38 - 1.64) | 0.53 |  |
|  | Older mothers | East Asian ^†^ | 3 | 1.45 (0.72 -2.90) | 0.30 | 0.324 | 1.45 (0.37 - 5.64) | 0.36 | 0.24 |
| Vegetable protein | Dietary assessment during pregnancy | East Asian | 2 | 1.15 (0.80 – 1.64) | 0.45 | 0 | 1.16 (0.22 – 6.20) | 0.47 | 0.01 |
|  |  | South/ South-east Asian | 1 | 1.78 (0.99 - 3.20) | 0.05 |  | 1.78 (0.99 - 3.20) | 0.05 |  |
|  | Older mothers | East Asian ^†^ | 2 | 1.15 (0.82 - 1.610 | 0.43 | 0 | 1.15 (0.31 - 4.20) | 0.40 | ≤0.01 |

## Table I: Summary of the cultural sensitivity of dietary interventions and assessment tools utilised within included non-white studies. Table summarising interventions and assessment tools used for non-white studies and evaluation of whether these interventions/tools are culturally sensitive. A RCTS. B. Observational studies. AGHE: Australian Guide to Healthy Eating. DNCT: Diabetes Nutrition and Complications Trial. EVOO: Extra Virgin Olive Oil. FFQ: Food Frequency Questionnaire. USDA: US Department of Agriculture.

**A**

| **Study** | **Ethnicity** | **Was the intervention culturally tailored?** | **Details on how the intervention was ethnically tailored for the individual participants** |
| --- | --- | --- | --- |
| Assaf-balut, 2017 [1] | Mediterranean | Yes | MedDiet recommendations. Treatment group supplemented with 40ml of EVOO and 25-30g of pistachios daily |
| Markovic, 2016 [2] | Australian Nationals | No | Diets were not ethnically tailored but individualised dietary consultations were provided. In the case of noncompliance alternative foods that were suitable for the intervention diets (low GI / high-fiber moderate GI) were suggested. |
| Opie, 2016 [3] | Asian and White European | Yes | Intervention focused on AGHE guidelines. Sustainable, patient driven goals were provided in a culturally sensitive manner (in relation to foods, aversions, allergies/intolerances, preferences, medical needs and food insecurity) |
| Sahariah, 2016 [5] | Asian | Yes | Daily snack resembling local street food including fresh samosas and fritters fried in sunflower oil |
| Simmons, 2017 [6] | Mediterranean populations* | No | Healthy dietary recommendations involving face-to-face coaching sessions. The intervention was not ethnically tailored |

**B**

| **Study** | **Ethnicity** | **Was the intervention culturally sensitive?** | **Details on how the intervention was ethnically tailored for the individual participants** |
| --- | --- | --- | --- |
| Assaf-Balut, 2018[1] | Mediterranean | Yes | DNCT and MEDAS questionnaires validated in older Spanish adults within the PREDIMED to have a moderate correlation |
| Dominguez, 2014[12] | Mediterranean | Yes | Previously validated FFQ. Nutrient intake assessed via Spanish food composition tables |
| Donazar-Ezcurra, 2017[13] | Mediterranean | Yes | Previously validated FFQ. Nutrient intake assessed via Spanish food composition tables |
| He, 2015[16] | Asian | No | FFQ validated within a subsample of study participants and not an external cohort |
| Hu, 2019[18] | Asian | Yes | Food frequency questionnaire contained 25 food items which represented commonly consumed Chinese dishes. Daily food and nutritional intakes calculated using Chinese food composition databases. Tool not previously validated |
| Karamanos, 2014[36] | Mediterranean | Yes | Dietary questionnaire previously validated in 8 Mediterranean countries |
| Lamyian, 2017[19] | Tehranian | Yes | Validated FFQ including food commonly consumed by Iranians. Composition of foods assessed via USDA due to incompleteness of the Iranian Food Composition table. |
| Liang, 2018[21] | Asian | Yes | FFQ and 24-hr dietary recall previously validated in a Chinese population. Total energy and nutrient intake reported to reflect Chinese food |
| Looman, 2018[22] | Australian Nationals | No | Nutrient intake assessed via the use of national government food database for Australian foods validated in women of reproductive age. No information on ethnicity. |
| Mak, 2018[23] | Asian | Yes | FFQ validated in western China, with the exception of 10 oils/ condiments deemed too difficult to estimate |
| Marí-Sanchis, 2018[24] | Mediterranean | Yes | Previously validated FFQ including dietary habits common to those consuming a Mediterranean diet. |
| Pang, 2017[27] | Asian | Yes | 24-hour recall and 3-day food diary performed by trained professionals. Database of locally available foods used to assess nutrient composition of food along with food labels and nutrient software. |
| Tajima, 2017[29] | Asian | Yes | Weighted 3-day food diary. Nutrient intake assessed via standard tables of food composition from Japan. |
| Yi, 2017[32] | Asian | Yes | FFQ validated in pregnant women within Taiwan. Nutrient and energy intake assessed based on estimates from the China Food Composition table |
| Yong, 2020[33] | Asia | Yes | Semi quantitative FFQ with a previously shown good reliability within pregnant Malaysian women |
| Zhou, 2018[16] | Asia | Yes | Previously validated semi-quantitative FFQ with shown reliability within urban-dwelling pregnant women from central China |

## Appendix A: Search Strategy

‘(Gestational diabetes OR "Diabetes, Gestational/diagnosis"[Mesh] OR "Diabetes, Gestational/epidemiology"[Mesh] OR "Diabetes, Gestational/diet therapy"[Mesh]) AND (ethnicit* or ethnic or native or minorit* or “high risk” or race or "Population Groups"[Mesh] OR "Ethnic Groups"[Mesh] OR "Race Factors"[Mesh] OR "Minority Groups"[Mesh]) AND (Diet* OR Dietary OR “Feeding behaviour*” OR “Eating behaviour*” OR Eating OR “Feeding habit*” OR “Nutritional habit*”OR “Eating habit*” OR Food OR “food consumption*” OR “Nutritional consumption” OR Calorie OR “Calorie consumption” OR Caloric intake OR “Diet*pattern*” OR “Eating pattern*” OR “Nutritional intake” OR Cooking OR "Diet"[Mesh] OR "Protective Factors"[Mesh] OR "Drinking"[Mesh] OR "Energy Intake"[Mesh] OR "Diet Therapy"[Mesh] OR "Nutrition Therapy"[Mesh] OR "Feeding Behavior"[Mesh] OR "Nutritional Status"[Mesh] OR "Food"[Mesh] OR "Nutrients"[Mesh] OR "Eating"[Mesh:NoExp] OR "Cooking"[Mesh:NoExp])AND (Prospective OR Retrospective OR Longitudinal OR Cohort OR follow-up OR observational or Randomised Controlled Trial* or controlled trial*, or clinical trial*, or clinical stud* OR "Epidemiologic Studies"[Mesh] OR "Nutrition Surveys"[Mesh] OR AND "Health Surveys"[Mesh:NoExp] OR "Diet Records"[Mesh] OR "Nutritional Sciences"[Mesh] OR "Clinical Studies as Topic"[Mesh] OR "Observational Studies as Topic"[Mesh])AND (“pre-pregnancy” or “pre pregnancy” or “early pregnancy” or “prenatal” or “pre-natal” or “pregnant” or “pregnancy” or "Prenatal Nutritional Physiological Phenomena"[Mesh] OR "Prenatal Care"[Mesh] OR "Preconception Care"[Mesh]). Limited to humans/ English

## Appendix B: ADA Risk Of Bias Tool Guide

Method: The tool was modified to exclude part of question 5, as it is unfeasible to blind participants on their food intake. Studies with ≥ 6 questions assigned as negative, were classified as having high risk of bias. When ≥ 10 studies were meta-analysed, publication bias was assessed by visual inspection of funnel plots (STATA, version 5).[37] When studies reported multiple diets that could be combined as the same exposure classification (such as two variations of the healthy diet) these were grouped as a single exposure. This was done due to the inability to aggregate effect sizes presented on a categorical variable without knowing whether each quartile of the diets contain the same study participants. To assess the impact of this on type II error, all estimates but one were randomly dropped for each study that reported multiple estimates that matched to one exposure, and the impact on the overall effect estimate for that exposure was assessed.

Bolded questions illustrate questions classified as having a high importance by the tool.


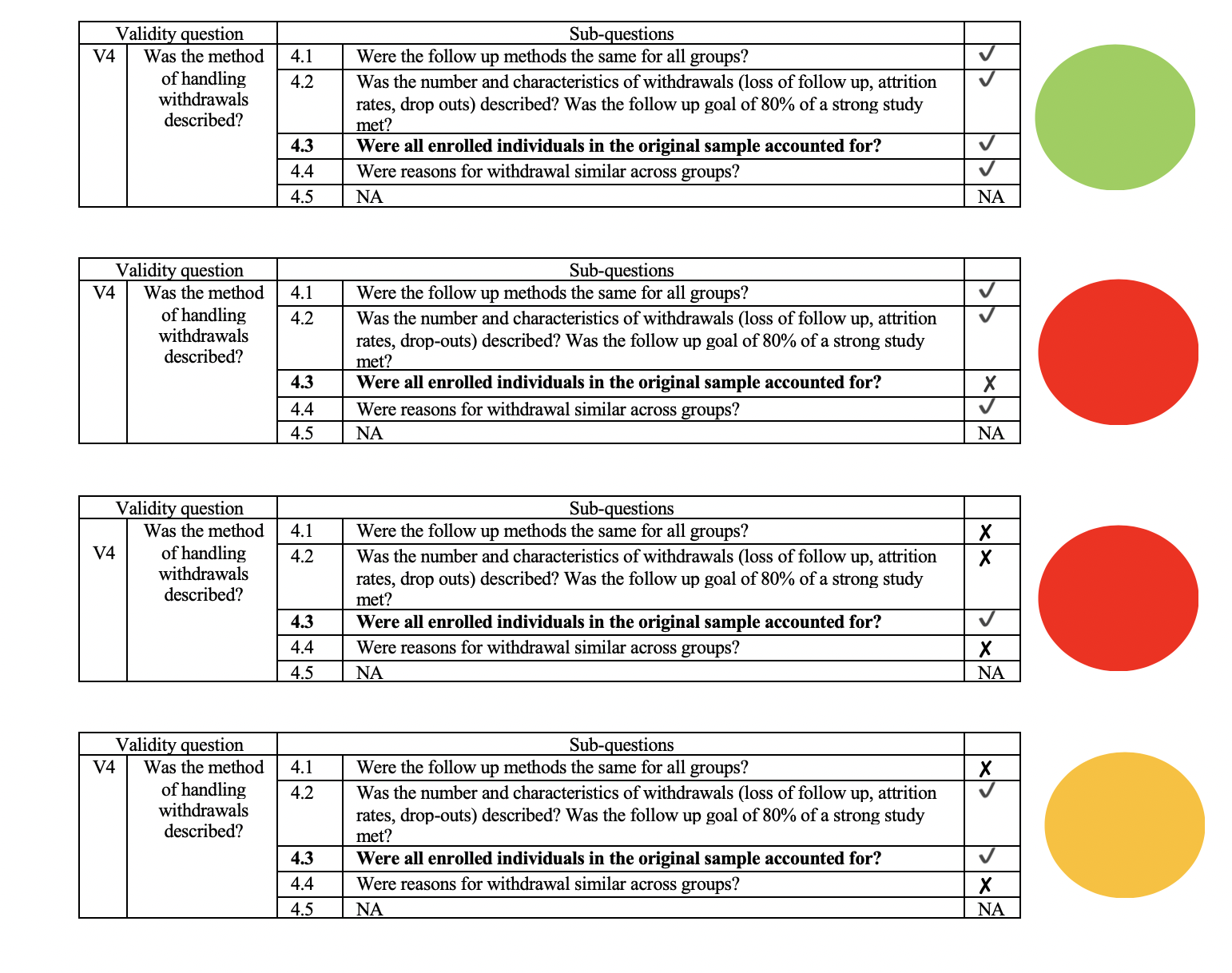


## Appendix C: Grouping of studies into the dietary exposures used within this study.

Exposures were excluded if they were not identified in more than one study or were not measured on the same scale in more than one study. FFQ= Food Frequency Questionnaire, TFD: Three-day food diary

| Study | Included exposures | Matched pattern | Excluded exposures |
| --- | --- | --- | --- |
| Assaf-Balut, 2017 | Med diet supplemented with olive oil and pistachios | Mediterranean diet, ‘Healthy’ diet | - |
| Assaf-Balut, 2018 | High adherence to med diet | Mediterranean diet, ‘Healthy’ diet | Moderate adherence to med diet |
| Badon, 2016 | Healthy diet | ‘Healthy’ diet | - |
| Bao, 2013 | Animal protein, Vegetable protein, Total protein, Protein sources (unprocessed red meat, processed red meat, poultry, fish, eggs, nuts, legumes) | Animal protein, Vegetable protein, High protein, Fish | Protein sources (unprocessed red meat, processed red meat, poultry, fish, eggs, nuts, legumes) |
| Bao, 2014 | Vegetable LCD diet, Animal LCD score  Overall LCD score, | Plant-based pattern, Meat pattern, High protein | - |
| Bao, 2014 | Frequency of fried food consumption (pre-pregnancy) | Fast/Fried food, ‘Unhealthy’ diet | Frequency of fried food consumption (during pregnancy) |
| Bao, 2016 | - | - | Potato consumption |
| Bowers, 2011 | - | - | Dietary iron |
| Bowers, 2012 | Total fat | - | Animal fat, Animal fat adjusted, Vegetable fat, Vegetable fat adjusted, Cholesterol, Cholesterol adjusted |
| Chen, 2009 | - | - | Sugar Sweetened Beverages |
| Dominguez, 2014 | Fast food consumption | Fast/Fried food, ‘Unhealthy’ diet | - |
| Donazar-Ezcurra, 2017 | Western dietary pattern, Mediterranean dietary pattern | Western diet, Mediterranean diet, ‘Healthy’ diet, ‘Unhealthy’ diet | - |
| Farland, 2015 | - | - | Sweet, salt, savoury, starchy |
| Flynn, 2016 | Processed pattern, fruit and veg pattern | Western diet, Plant-based pattern, ‘Healthy’ diet , ‘Unhealthy’ diet | Snacks, African pattern |
| Fulay, 2018 | - | - | Continuous: Dash score, Dash OMNI score |
| Gicevic, 2018 | AHEI-2010 | Healthy Recommendations, ‘Healthy’ diet | MDD-W, FGI, PDQS |
| He, 2015 | Vegetable, Prudent, Sweets and Seafood, Protein rich | Plant-based pattern, Prudent, Sweets and Seafood, High protein , ‘Healthy’ diet, ‘Unhealthy’ diet | - |
| Hu, 2019 | Traditional (TFD), Sweet foods (TFD), Fish-seafood (FFQ), | Traditional, Sweets and Seafood, Fish, ‘Unhealthy’ diet | Fried food-beans (TFD), Whole grain - seafood (TFD), Protein-sweets (FFQ), Traditional (FFQ) |
| Hrolfsdottir, 2019 | Unhealthy diets score (categorical) | ‘Unhealthy’ diet | Unhealthy diet score (continuous) |
| Karamanos, 2014 | High adherence to a MedDiet | Mediterranean diet, ‘Healthy’ diet | - |
| Lamyian, 2017 | Total fast food | Fast/fried food, ‘Unhealthy’ diet | French fries, Hamburger, Bologna, Sausages, Pizza |
| Le-Donne | All fish types | Fish | Large size oily fish, small size oily fish, lean fish, shellfish with low DHA. |
| Ley, 2011 | - | - | Continuous (per 1 SD change): Total energy, protein, carbohydrate, total fat, saturated fat, trans fat, monounsaturated fat, polyunsaturated fat, P:S ratio, energy adjusted intake: cholesterol, energy adjusted intake: total fibre, energy adjusted intake: grain fibre, energy adjusted intake: vegetable and fruit fibre |
| Liang, 2018 | Total protein, animal protein, vegetable protein (during pregnancy), fish and seafood | Animal protein, Vegetable protein, High protein, Fish | Eggs, dairy and dairy products, beans and nuts |
| Looman, 2018 | Carbohydrates, Glycaemic index, LCD score | Carbohydrates, Healthy, High protein | Total sugars, total dietary fibre, glycaemic loads, white bread, high-fibre bread, cereal |
| Osorio-Yáñez, 2017 | Total fried food consumption | Fast/fried food, ‘Unhealthy’ diet | Fried fish, fried chicken, fried potatoes, Snack chips, doughnuts |
| Pang, 2017 | Animal protein, vegetable protein, total protein | Animal protein, Vegetable protein, High protein | Red meat protein, poultry protein, seafood protein, egg protein, dairy protein, rice noodle and desert protein, bean protein |
| Park, 2013 | - | - | Continuous exposure: Saturated fat intake (% of RDA) |
| Radskey, 2008 | - | - | Continuous exposures: Energy (100kcal), Carbohydrate (% energy), Glycaemic load (SD), trans fat (% energy), saturated fat (% energy), P:S ratio (per 1 %), monounsaturated fat  (% energy), n-3 fatty acids (1g), fibre (5g), whole grains (serving), red meat (weekly serving), processed meat (weekly serving) |
| Rifas-shiman, 2009 | - | - | Continuous exposure: AHEIP score |
| Rono, 2018 | Healthy diet recommendation (Nordic) | Healthy diet recommendations, ‘Healthy’ diet |  |
| Marí-Sanchis, 2018 | Meat consumption | Meat pattern | Red meat, Processed meat, Poultry, Ham, Heme iron, Non-heme iron, Total iron, Iron with supplements |
| Mak, 2018 | Plant-based pattern, Meat-based pattern, High protein-low starch pattern | Plant-based pattern, Meat-based pattern, High protein, ‘Healthy’ diet | - |
| Markovic, 2016 | Low GI diet | ‘Healthy’ diet | - |
| Mohanty, 2015 | Total seafood intake | Fish | - |
| Qui, 2011 |  | - | Eggs |
| Sahariah, 2016 | Diet supplement made with leafy green vegetables, fruit and milk | ‘Healthy’ diet | - |
| Saldana, 2004 |  |  | Continuous exposure: Addition/ substitution models |
| Simmons, 2017 | Healthy diet | Healthy Recommendations, ‘Healthy’ diet | - |
| Seymour, 2016 | - | - | Vegetable-fruit-rice-based-diet, Seafood-noodle-based-diet, Pasta-cheese-processed-meat-diet |
| Shin, 2015 | - | - | ‘High nuts, seeds, fat and soybean, low milk and cheese’, ‘High added sugar and organ meats, low fruits, vegetable and seafood pattern’ |
| Tajima, 2017 | Fat, carbohydrates | Fat, Carbohydrates | Fibre, protein |
| Schoenaker, 2015 | ‘Meat, snacks and sweets’, ’Mediterranean-style’, ‘cooked vegetables’ | Western diet, Mediterranean diet, Plant-based pattern, ‘Healthy’ diet, ‘Unhealthy’ diet | ‘Fruit and low fat-dairy’ |
| Schoenaker, 2018 | - | - | Mediterranean diet score (continuous exposure) |
| Tobias, 2012 | aMED, aHEI | Mediterranean diet, ‘Healthy’ diet | DASH |
| Tryggvadottir, 2016 | Prudent dietary pattern, Healthy eating index | Prudent pattern, ‘Healthy’ diet | Seafood, Eggs, Vegetables, Fruit and berries, Vegetable oil, Nuts and seeds, Pasta/couscous, Breakfast cereal, Coffee, Soft drinks, French fries |
| Opie, 2016 | AGHE diet | ‘Healthy’ diet |  |
| Wang, 2015 |  | - | TG/ HDL-C |
| Yi, 2017 | Western pattern score, Traditional pattern, Prudent pattern | Western pattern, Traditional pattern, Prudent pattern, ‘Healthy’ diet, ‘Unhealthy’ diet | Mixed pattern |
| Yong, 2020 | DP1, DP2, DP3 | Plant based (DP1), Western (DP3), ‘Healthy’ diet, ‘Unhealthy’ diet | DP3 (Condiment, spices, sugar, spread and creamer intake() |
| Zhang, 2006 | Western pattern score, Prudent pattern score | Western pattern, Prudent pattern, ‘Healthy’ diet, ‘Unhealthy’ | - |
| Zhang 2014 | AHEI | ‘Healthy’ diet | - |
| Zhou, 2018 | Beans- vegetable, Fish-meat-eggs, Total protein,  Animal protein, Vegetable protein | Plant-based pattern, Meat pattern, High protein, Animal protein, Vegetable protein, ‘Healthy’ diet | Nuts- whole grains, Organs-poultry-seafood, Rice-wheat-fruits, total fat, vegetable protein, total carbohydrate intake, total protein: total carbohydrate intake |

REFERENCES:

1. Assaf-Balut C, Garcia de le Toore N, Fuentes M, Duran A, Bodio E, del Valle L. A High Adherance to Six Food Targets of the Mediterranean Diet in the Late First Trimester is Associated with Reduction in the Risk of Materno-Foetal Outcomes: The St. Carlos Gestational Diabetes Mellitus Prevention Study. Nutrients. 2018;11(66).

2. Markovic TP, Muirhead R, Overs S, Ross GP, Chun J, Louie Y, et al. Randomized Controlled Trial Investigating the Effects of a Low – Glycemic Index Diet on Pregnancy Outcomes in Women at High Risk of Gestational Diabetes Mellitus : The GI Baby 3 Study. Diabetes Care. 2016;39(January):31-8.

3. Opie RS, Neff M, Tierney AC. A behavioural nutrition intervention for obese pregnant women : Effects on diet quality , weight gain and the incidence of gestational diabetes. The Australian and New Zealand Journal of Obsterics and Gynaecology. 2016;56(4):364-73.

4. Rönö K, Elisabeth N, Miira G, Klemetti M, Stach-lempinen B, Huvinen E, et al. Effect of a lifestyle intervention during pregnancy — findings from the Finnish gestational diabetes prevention trial (RADIEL). Journal of Perinatology. 2018;38:1157-64.

5. Sahariah SA, Potdar RD, Gandhi M, Kehoe SH, Brown N, Sane H, et al. A Daily Snack Containing Leafy Green Vegetables, Fruit, and Milk before and during Pregnancy Prevents Gestational Diabetes in a Randomized, Controlled Trial in Mumbai, India. The Journal of Nutrition. 2016;146(7):1453S-60S.

6. Simmons D, Devlieger R, Assche AV, Jans G, Galjaard S, Corcoy R, et al. Effect of Physical Activity and/or Healthy Eating on GDM Risk: The DALI Lifestyle Study. The Journal of clinical endocrinology and metabolism. 2017;102(3):903-13.

7. Badon SE, Enquobahrie DA, Wartko PD, Miller RS, Qiu C, Gelaye B, et al. Healthy Lifestyle During Early Pregnancy and Risk of Gestational Diabetes Mellitus. American Journal of Epidemiology. 2017;186(3):326-33.

8. Bao W, Bowers K, Tobias DK, Hu FBH, Zhang C. Prepregnancy Dietary Protein Intake, Major Dietary Protein Sources, and the Risk of Gestational Diabetes Mellitus. Diabetes Care. 2013;36:2001-8.

9. Bao W, Bowers K, Tobias DK, Olsen SF, Chavarro J, Vaag A, et al. Prepregnancy low-carbohydrate dietary pattern and risk of gestational diabetes mellitus: a prospective cohort study. American Journal of Clinical Nutrition. 2014;99:1378-84.

10. Bao W, Tobias DK, Olsen SF, Zhang C. Pre-pregnancy fried food consumption and the risk of gestational diabetes mellitus: a prospective cohort study. NIH Public Access. 2015;57(12):2485-91.

11. Bowers K, Tobias DK, Yeung E, Hu FB, Zhang C. A prospective study of prepregnancy dietary fat intake and risk of gestational diabetes. American Journal of Clinical Nutrition. 2012;95:446-53.

12. Domingues LJ, Martínez-Gonzàlez MA, Basterra-Gortari FJ, Gea A, Barbagallo M, Bes-Restrollo M. Fast Food Consumption and Gestational Diabetes Incidence in the SUN Project. 2014;9(9):1-7.

13. Donazar-ezcurra M, Burgo CL-d, Martinez-gonzalez MA, Basterra-gortari FJ, Irala JD, Bes-rastrollo M. Pre-pregnancy adherences to empirically derived dietary patterns and gestational diabetes risk in a Mediterranean cohort: the Seguimiento Universidad de Navarra (SUN) project. British Journal of Nutrition. 2017;118(9):715-21.

14. Flynn AC, Seed PT, Patel N, Barr S, Bell R, Briley AL, et al. Dietary patterns in obese pregnant women: Influence of a behavioral intervention of diet and physical activity in the UPBEAT randomized controlled trial. International Journal of Behavioral Nutrition and Physical Activity. 2016;13(124):1-12.

15. Gicevic S, Gaskins AJ, Fung TT, Rosner B, Tobias K, Isanaka S, et al. Evaluating pre-pregnancy dietary diversity vs . dietary quality scores as predictors of gestational diabetes and hypertensive disorders of pregnancy. PLoS ONE. 2018:1-14.

16. Zhou X, Chen R, Zhong C, Wu J, Li X, Li Q, et al. Maternal dietary pattern characterised by high protein and low carbohydrate intake in pregnancy is associated with a higher risk of gestational diabetes mellitus in Chinese women: a prospective cohort study. British Journal of Nutrition. 2018;210:1045-55.

17. Hrolfsdottir L, Gunnarsdottir I, Birgisdottir BE, Hreidarsdottir IT, Kr.Smarason A. Can a Simple Dietary Screening in Early Pregnancy Identify Dietary Habits Associated with Gesational Diabetes. nutrients. 2019;11(1868):1-12.

18. Jiajin H, Oken E, Izzuddin MA, Pi-Id L, Gao M, Wei X, et al. Dietary Patterns during Pregnancy Are Associated with the Risk of Gestational Diabetes Mellitus: Cohort Study. nutrients. 2019;11(405):1-15.

19. Lamyian M, Hosseinpour-Niazi S, Mirmiran P, M. BL, Goshtasebil A, Azizi F. Pre-Pregnancy Fast Food Consumption Is Associated with Gestational Diabetes Mellitus among Tehranian Women. Nutrients. 2017;9(216).

20. Le Donne M, Alibrandi A, Vita R, Zanghı D, Triolo O, Benvenga S. Does eating oily fish improve gestational and neonatal outcomes ? Findings from a Sicilian study. Women and Birth. 2016;29:50-7.

21. Liang Y, Gong Y, Zhang X, Yang D, Zhao D, Quan L. Dietary Protein Intake, Meat Consumption, and Dairy Consumption in the Year Preceding Pregnancy and During Pregnancy and Their Associations With the Risk of Gestational Diabetes Mellitus: A Prospective Cohort Study in Southwest China. Frontiers in Endocrinology. 2018;9(October):1-9.

22. Looman M, Schoenaker DAJM, Soedamah-muthu SS, Geelen A, Feskens EJM, Mishra GD. Pre-pregnancy dietary carbohydrate quantity and quality, and risk of developing gestational diabetes: the Australian Longitudinal Study on Women’s Health. 2018:435-44.

23. Mak JKL, Pham NM, Lee AH, Tang L, Pan X-f, Binns CW, et al. Dietary patterns during pregnancy and risk of gestational diabetes : a prospective cohort study in Western China. Nutrition Journal. 2018;17(107):1-11.

24. Sanchis AM, Díaz G, Javier JF, Gortari B, De C, Arrillaga F, et al. Association between pre-pregnancy consumption of meat , iron intake , and the risk of gestational diabetes : the SUN project. European Journal of Nutrition. 2018;57(3):939-49.

25. Mohanty AF, Siscovick DS, Williams MA, Thompson ML, Burbacher TM, Enquobahrie DA. Periconceptional seafood intake and pregnancy complications. Public Health Nutrition. 2015;19(10):1795-803.

26. Osorio-Yáñez C, Gelaye B, Qiu C, Bao W, Cardenas A, Enquobahrie DE, et al. Maternal Intake of Fried Foods and Risk of Gestational Diabetes Mellitus. Annals of Epidemiology. 2017;27(6):384-90.

27. Pang WW, Colega M, Cai S, Chan YH, Chen L-w, Soh S-e, et al. Higher Maternal Dietary Protein Intake Is Associated with a Higher Risk of Gestational Diabetes Mellitus in a Multiethnic Asian. The Journal of Nutrition. 2017(10):653-60.

28. Schoenaker DAJM, Soedamah-muthu SS, Callaway LK, Mishra GD. Pre-pregnancy dietary patterns and risk of gestational diabetes mellitus: results from an Australian population-based prospective cohort study. Diabetologia. 2015;58:2726-35.

29. Tajima R, Yachi Y, Tanaka Y, Kawasaki YA. Carbohydrate intake during early pregnancy is inverselyassociated with abnormal glucose challenge test results in Japanese pregnant women. Diabtetes Metabolism Research and Reviews. 2017;33.

30. Tobias DK, Zhang C, Chavarro J, Bowers K, Rich-edwards J, Rosner B, et al. Prepregnancy adherence to dietary patterns and lower risk of gestational diabetes mellitus. American Journal of Clinical Nutrition. 2012;96:289-95.

31. Tryggvadottir EA, Medek H, Birgisdottir BE, Geirsson RT, Gunnarsdottir I. Association between healthy maternal dietary pattern and risk for gestational diabetes mellitus. 2015;70(2):237-42.

32. Yi DUH, Hong J, Karmin O, Bo C, Ji XL. Association of Dietary Pattern during Pregnancy and Gestational Diabetes Mellitus : A Prospective Cohort. Biomedical and Environmental scientists. 2017;30(12):887-97.

33. Yong HY, Shariff ZM, Yusof B-nM, Rejali Z. The association between dietary patterns before and in early pregnancy and the risk of gestational diabetes mellitus ( GDM ): Data from the Malaysian SECOST cohort. PLoS ONE. 2020:1-16.

34. Zhang C, Schulze MB, Solomon CG, Hu FB. A prospective study of dietary patterns, meat intake and the risk of gestational diabetes mellitus. Diabetologia. 2006;49:2604-13.

35. Zhang C, Tobias DK, Chavarro JE. Adherence to healthy lifestyle and risk of gestational diabetes mellitus : prospective cohort study. the BMJ. 2014;5450(September):1-11.

36. Karamanos B, Thanopoulou A, Anastasiou E, Assaad-Khalil S, Albache N, Bachaoui M, et al. Relation of the Mediterranean diet with the incidence of gestational diabetes. European journal of clinical nutrition. 2013.

37. Higgins JPTGS. 10.4.3.1 Recommendations on testing for funnel plot asymmetry. 2011. p. 10.4.3.1-.4.3.1.
