# Supplementary figures and images for: Ethnic-specific associations between dietary consumption and gestational diabetes mellitus incidence: A meta-analysis
Source: PLOS Glob Public Health. 2022 May 11;2(5):e0000250. doi: 10.1371/journal.pgph.0000250 (PMC10021780; doi:10.1371/journal.pgph.0000250)

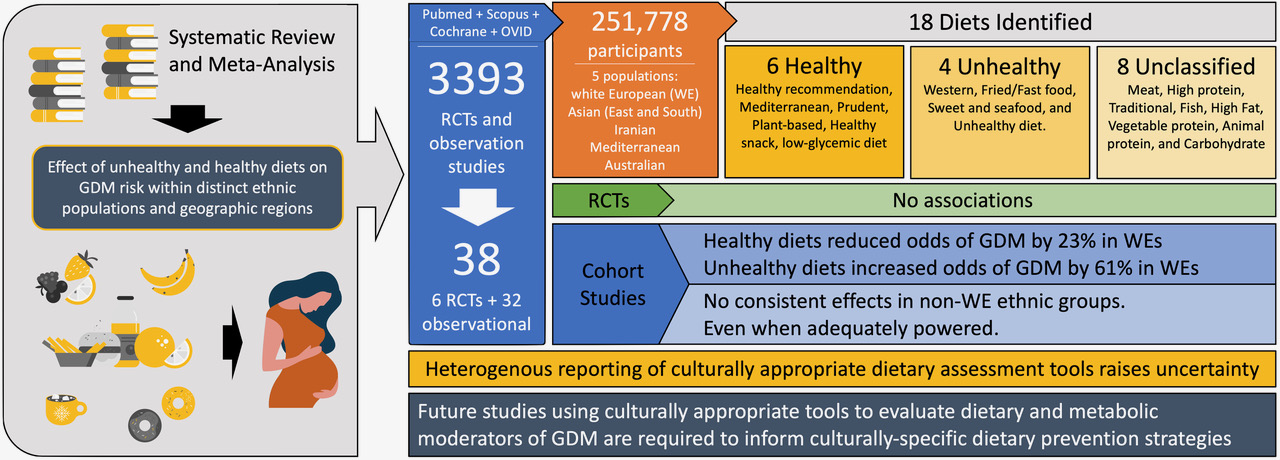

Supplement: S1 Graphical Abstract — (TIF) [file pgph.0000250.s002.tif]
